# Biotic interactions shape the realized niche of toxic cyanobacteria

**DOI:** 10.1038/s41559-026-03131-0

**Published:** 2026-07-20

**Authors:** Pinelopi Ntetsika, Stefanie Eyring, Ewa Merz, Marta Reyes, Benno Käch, Stephan B. Munch, Stuart R. Dennis, Marco Baity-Jesi, Francesco Pomati

**Affiliations:** 1 Department of Aquatic Ecology, Swiss Federal Institute of Aquatic Science and Technology (Eawag), Dübendorf, Switzerland; 2 Integrative Oceanography Division, Scripps Institution of Oceanography, University of California San Diego, La Jolla, CA USA; 3 Department of Applied Mathematics, University of California, Santa Cruz, CA USA; 4 Scientific IT, Swiss Federal Institute of Aquatic Science and Technology (Eawag), Dübendorf, Switzerland; 5 Department of Systems Analysis, Integrated Assessment and Modelling, Swiss Federal Institute of Aquatic Science and Technology (Eawag), Dübendorf, Switzerland

**Keywords:** Microbial ecology, Freshwater ecology, Population dynamics, Limnology

## Abstract

Cyanobacterial blooms increasingly threaten vital freshwater ecosystems, with harmful impacts exacerbated by climate change and eutrophication. Cyanobacteria produce toxic metabolites whose concentrations increase with biomass accumulation, posing direct risks to drinking water, aquatic ecosystems and recreational use while generating substantial economic costs. Despite extensive research on temperature and nutrient effects, our explanatory and predictive capacity remains limited. We propose that this limitation stems from insufficient understanding of how biotic and abiotic factors interact to modify cyanobacterial net growth and, consequently, biomass accumulation. Here, using five years of daily monitoring data from a eutrophic lake and causal inference via state-space reconstruction modelling, we show that interactions with co-occurring plankton taxa fundamentally reshape the realized niche of bloom-forming cyanobacteria over environmental gradients. Biotic interactions shift temperature boundaries for net growth by up to 13 °C and phosphorus requirements by over 20 μg l^−1^ in toxic *Microcystaceae* and *Dolichospermum* spp. Grazing inhibits bloom formation across cyanobacterial taxa, while facilitation by other phytoplankton may allow blooms at unexpectedly low temperatures and phosphate concentrations. These findings address a fundamental research gap—how biotic interactions shape realized niches in natural ecosystems—while offering practical insights for harmful algal bloom management.

## Abstract (web summary)

Using five years of daily lake plankton monitoring data and empirical dynamic modelling, this study shows that grazing, competition and facilitation reshape the temperature and nutrient conditions required for toxic cyanobacterial blooms.

## Main

Cyanobacterial blooms impact freshwater ecosystems, causing substantial economic losses in services like recreation, drinking water and fisheries^1,2^. The global prevalence of these blooms has increased over recent decades, with projections suggesting continued expansion^1,3^. Beyond the economic impacts, cyanobacteria produce bioactive metabolites that are toxic to animals and potentially harmful to humans, particularly when biomass accumulates to high concentrations^3,4^. Despite their ecological and economic importance, predicting bloom occurrence and magnitude remains challenging. Current research on cyanobacterial blooms primarily emphasizes abiotic factors—nutrient availability (phosphorus and nitrogen) and temperature—as key drivers of growth and toxic outbreaks^3,5–7^. However, bloom dynamics result from the balance between cell growth and loss processes, including mortality and physical removal^8^. While we recognize that blooms emerge from complex networks of abiotic factors and interactions with grazers and competitors^9^, the specific role of biotic interactions in natural systems remains poorly understood. This limitation potentially undermines our forecasting models, which may over- or underestimate bloom occurrence and magnitude when biotic factors are neglected^10^. Here we address this critical knowledge gap—the insufficient understanding of how biotic interactions shape cyanobacterial blooms in natural systems—and hypothesize that these interactions provide crucial mechanistic understanding to explain and ultimately predict the complex ecological dynamics driving bloom formation.

While we hypothesize that biotic interactions are needed for understanding bloom formation, current knowledge of these interactions stems primarily from controlled laboratory experiments with limited environmental relevance, taxa diversity and breadth of timescales. Studies have focused predominantly on competitive and predator–prey dynamics, with less attention to facilitative mechanisms. Cyanobacteria possess well-documented competitive advantages in warm, nutrient-rich environments, including CO_2_-concentrating mechanisms, specialized pigmentation, nitrogen fixation and buoyancy control^3^. Their bioactive metabolites may play a role in stress responses^11^ and as mediators of biotic interactions, particularly with zooplankton^12^. Cyanobacteria’s low nutritional value, toxicity and large colony size make them unfavourable prey, with some zooplankton (for example, copepods) promoting cyanobacterial dominance through selective grazing that targets competing algae^13^. Conversely, microzooplankton (especially rotifers) and cladocerans, may control blooms through direct consumption^14,15^. Critically, these biotic interactions are dynamic, shifting with environmental conditions^16,17^ and through zooplankton physiological and behavioural adaptations occurring on short timescales^12,14^. The research gap lies specifically in understanding how dynamic biotic interactions shape cyanobacteria’s realized niche in natural communities, across temperature and phosphorus gradients—the very environmental factors most affected by climate change and eutrophication.

Given the focus of this study on the realized niche, we clarify the specific definitions we employ in this context. The fundamental niche represents the set of environmental conditions under which a species can sustain a positive intrinsic growth rate (*r* > 0) in the absence of biotic interactions, defined by physiological constraints such as metabolic, thermal and resource uptake limits^18,19^. The realized niche is the subset of the fundamental niche where a species maintains a positive net growth rate (nGR)—that is, intrinsic growth minus losses due to biotic interactions such as competition, facilitation, predation or parasitism. It represents the actual ecological conditions in which the species persists in nature, accounting for both physiological limits and ecological processes that affect fitness^18,19^.

Here we investigate whether and how interactions between cyanobacteria and co-occurring plankton taxa influence cyanobacterial realized niches, specifically focusing on their nGR across abiotic gradients^8,9^. This also allows us to study bloom dynamics: instead of defining blooms based on fixed abundance or biomass thresholds, which are often arbitrary and strongly system-specific, we use nGRs (that is, cell division minus loss processes^8,9^) as a process-based metric to study the dynamics underlying biomass accumulation, thereby focusing on the progression towards bloom conditions rather than the bloom state itself. Positive nGR values indicate that biomass gains exceed losses on a daily scale, which can precede the visible onset of bloom conditions. Focusing on nGR allows us to identify the drivers that promote or inhibit cyanobacterial build-up and provides an earlier indication of bloom risk than biomass thresholds alone, thereby offering a useful tool for both researchers and stakeholders.

**Fig. 1:**
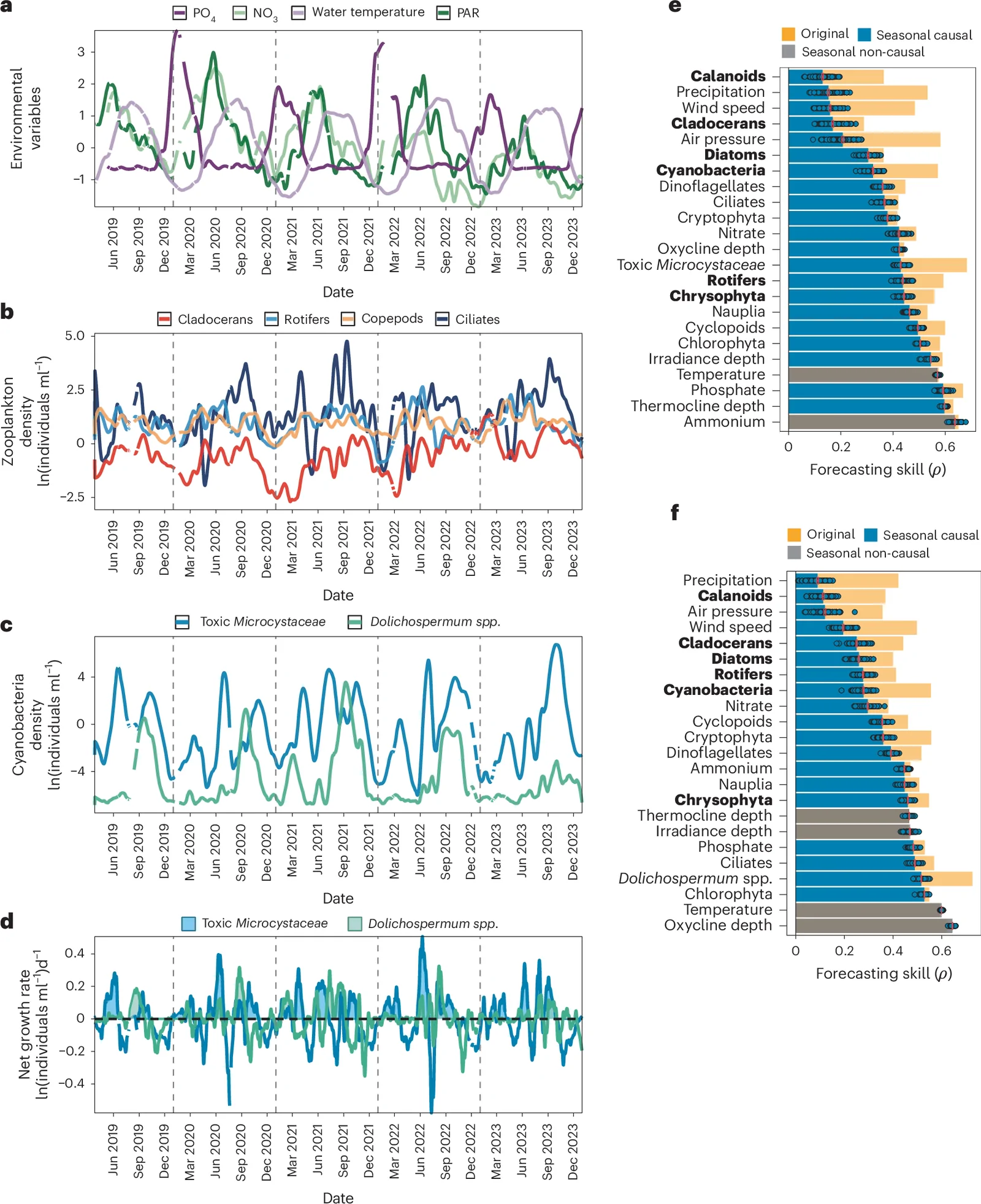
Environmental conditions and cyanobacterial population dynamics in Lake Greifensee (2019–2023). **a**, Seasonal patterns in the temperate eutrophic lake showing scaled values of water temperature (°C), PAR depth (m), phosphate (PO_4_^3−^, μg l^−1^) and nitrate (NO_3_^−^, mg l^−1^). Nutrients peak during winter circulation when temperatures are lowest, and reach a minimum in summer when temperature and irradiance are highest. **b**, Densities of key zooplankton groups. **c**, Densities of target cyanobacterial taxa. **d**, Cyanobacterial nGRs from consecutive daily logged density measurements. **e**,**f**, CCM analysis showing coupling strength between environmental variables and nGRs of toxic *Microcystaceae* (**e**) and *Dolichospermum* spp. (**f**). Orange bars (‘original’) represent coupling strength (convergence skill, *ρ*) between observed and predicted values from the original time series. Blue points show the individual coupling strengths obtained from 100 seasonal surrogate time series. Blue and grey bars show mean coupling strength from the seasonal surrogate time series, with red bars indicating the standard error (*n* = 100 seasonal surrogate time series). Blue bars (‘seasonal causal’) indicate significant causal links (≥95% of surrogate *ρ* < original *ρ*), while grey bars (‘seasonal non-causal’) indicate non-significant links (>5% of surrogate *ρ* ≥ original *ρ*). Biotic variables used in Figs. 3–5 are highlighted in bold. Strong seasonal coupling is marked by similar heights in orange and grey bars (for example, for oxycline depth, temperature, irradiance, thermocline depth, ammonium (NH_4_^+^) and phosphate). In contrast, strong daily-scale coupling is shown by orange bars exceeding blue bars. See Methods for a detailed description of all variables and units used in this analysis. Source data

We analyse daily data from Lake Greifensee (Switzerland) across five consecutive growing seasons (1,742 days), using an automated underwater microscope (Methods) that records organisms from 10 μm to 1 cm—spanning from cyanobacteria to invertebrate predators, herbivores of various sizes, and mixotrophic and photosynthetic protists—encompassing 83 taxa (Fig. 1 and Supplementary Tables 1 and 2). A neural network classifies each detected object to generate a daily time series of taxonomic abundances (Supplementary Figs. 1–3 and Supplementary Table 3). Concurrently, we monitor all major physical and chemical parameters influencing cyanobacterial growth, including water temperature, nutrients and meteorological conditions (Fig. 1 and Supplementary Fig. 4), with all abiotic data quality-controlled and aggregated (or predicted where needed) to daily resolution for time-series-based causal analysis (Methods).

Detecting ecological interactions in natural plankton communities is, however, challenging—dynamics are non-linear, relationships change with environmental conditions and seasonal patterns can create misleading correlations. We address this using empirical dynamic modelling (EDM), a framework that identifies causality in complex ecological time series. We first use convergent cross-mapping (CCM) to determine which plankton groups causally influence cyanobacterial net growth (rather than simply co-occurring seasonally). We then use dynamic models (S-maps) to quantify how these interactions shift across temperature and nutrient gradients, revealing how biotic factors modify the environmental conditions required for cyanobacteria to bloom.

## Results

### Cyanobacterial dynamics

Lake Greifensee is periodically affected by cyanobacterial blooms and associated cyanotoxins^20–22^ (Fig. 1). Our study focuses on two key groups: the toxic *Microcystaceae* group (*Microcystis* spp., *Aphanothece* spp., *Aphanocapsa* spp.) and *Dolichospermum* spp. *Microcystacae* are the most common potentially toxic bloom-forming cyanobacteria worldwide^23^, typically blooming in Greifensee during summer and autumn (Fig. 1c). Previous studies confirm the production of microcystins and numerous other bioactive metabolites from this group in the lake, including during our study period^20,22^. *Dolichospermum*, the second most common potentially toxic bloom-forming cyanobacterium globally, is known to produce both hepatotoxins and neurotoxins^3,24^. While rarely forming surface blooms in Greifensee, it reaches peak abundance in late summer, and bioactive metabolites potentially associated with this taxon are found during the study period^22^. Our high-resolution data reveal significant temporal fluctuations in both cyanobacteria (Fig. 1c). This provides a unique opportunity to examine growth dynamics and their relationships with biotic and abiotic factors at temporal scales directly relevant to bloom formation.

To quantify these dynamics and investigate the influence of biotic interactions, we present an nGR analysis based on high-frequency monitoring data. To estimate daily nGRs, we calculate the difference in the natural logarithm of taxon densities for each taxon between consecutive days, as recorded by the underwater microscope (Fig. 1d). This approach reflects the net daily gains or losses observed for each target taxon, and it is conceptually similar to the per capita growth rate estimates obtained from laboratory studies^8,25^. For each taxon, nGR_*i*_ = ln(*N*_*i*,*t*_) − ln(N_*i*,*t*__−__1_), where *i* is the cyanobacterial taxon, *N*_*t*_ is density at time *t* and *N*_*t*−1_ is density at time *t* − 1. Using tracked density observations from 2019 to 2023, we estimate the daily nGR for both toxic *Microcystaceae* and *Dolichospermum*. While the nGR patterns explain the duration and intensity of cyanobacterial peaks in densities, we observe how the maxima in nGRs and the resulting overall densities varied considerably over the studied years (Fig. 1c,d). These aperiodic fluctuations exemplify the hallmark characteristics of complex ecological systems like plankton communities: non-equilibrium and non-linear dynamics^26–28^.

The presence of these dynamics necessitates analytical approaches beyond conventional methods. Analysing such complex systems, where chaotic dynamics lead to bounded but aperiodic fluctuations with sensitive dependence on initial conditions, demands specialized modelling approaches. These methods must explicitly account for non-linear and state-dependent relationships, including dynamic changes in growth rates^29–32^. For these reasons, we use state-space reconstruction methods from the EDM framework, which do not rely on detailed mechanistic knowledge of the focal system and outperform traditional models in chaotic systems^33,34^, including plankton^28^, to study the high-frequency dynamics of cyanobacterial nGR as a function of their environmental conditions. We choose ecologically important environmental factors that are known to influence the physiology and interactions of planktonic organisms^35–37^, specifically cyanobacteria^3,6,38^.

Importantly, our nGR patterns integrate both intrinsic physiological growth and per capita interaction effects^25^, reflecting the combined influence of bottom-up resource availability and top-down or lateral biotic controls that jointly determine population dynamics. While we cannot fully disentangle direct physiological effects from ecological interactions by studying this metric, we acknowledge that biotic interactions can influence cyanobacterial physiology (for example, through altered colony formation or secondary metabolite production in response to grazing pressure), potentially modifying how these organisms respond to abiotic conditions. However, given the temporal frequency and scope of the study, we interpret the observed changes in nGR as reflecting ecological processes, particularly grazing pressure, competition and facilitation effects. We demonstrate below how they relate to the nGRs of two globally important cyanobacteria taxa.

### Coupling to environmental factors

We first ask how strongly biotic and abiotic environmental factors are coupled to cyanobacterial nGRs in our field dataset. Plankton communities are forced by seasonal changes in temperature and nutrients (Fig. 1a–c) that drive an ecological succession^36^. These abiotic factors, external to the plankton community, may create apparent synchrony (correlation) between non-interacting species that share similar environmental requirements^29^, or drive such interactions^17^. To estimate the strength of the direct coupling between abiotic or biotic factors and the nGR of cyanobacteria, we use CCM, a method that infers causation based on attractor reconstruction and forecasting, rather than from correlation^29^. Specifically, we compare the CCM *ρ* (cross-mapping skill) value calculated using the original time series with 100 seasonal surrogate time series created by randomly sampling days from the original data across different years within the same calendar weeks (Methods). Deterministic links at the daily scale that are significantly different from seasonal forcing are identified when at least 95% of the CCM predictive skill *ρ* of the surrogate time series is smaller than that of the original time series.

**Extended Data Fig. 1:**
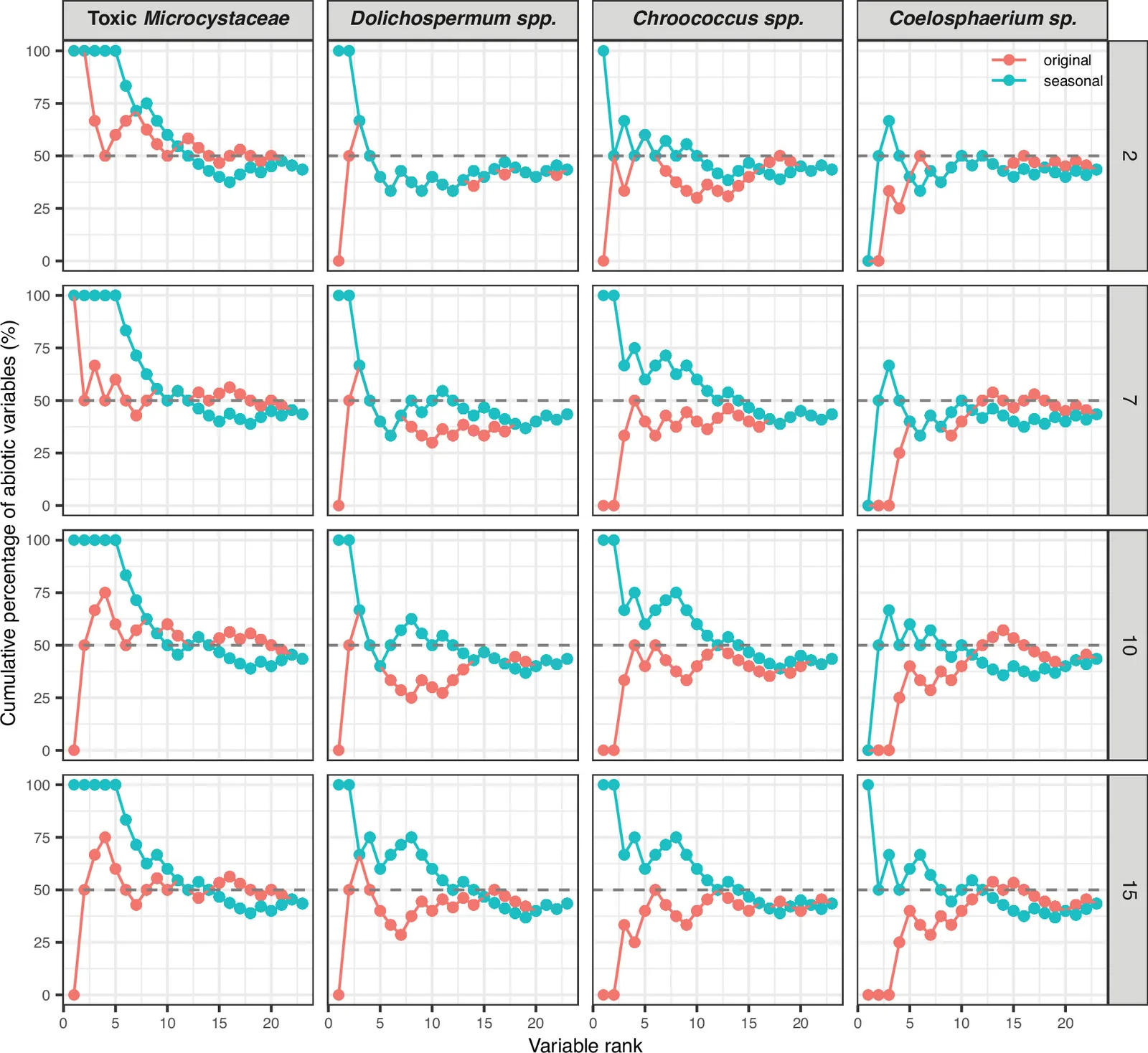
Cumulative contribution of abiotic predictors across ranked CCM variables, with increasing embedding dimensions. Cumulative percentage of abiotic variables among predictors ranked by convergent cross-mapping (CCM) skill (rho) at maximum library size (80%) (**see** Figs. S28–S29). For each cyanobacterial target taxon (columns) and embedding dimension (rows), variables were ordered from highest to lowest rho. The x-axis represents the progressive inclusion of ranked variables, and the y-axis shows the cumulative percentage of abiotic variables among those included ranks. Line colours distinguish CCM results from the original time series and seasonal surrogate analyses. Across embedding dimensions, **seasonal surrogate** CCM results (blue) show a consistently higher proportion of abiotic variables among the highest ranks, reflecting strong seasonal forcing. In contrast, the **original time series** (red) shows a lower cumulative percentage of abiotic predictors at the top ranks, indicating that biotic interactions contribute substantially to daily cyanobacterial growth dynamics. This pattern remains consistent across embedding dimensions, showing the independence of our inference from the choice of embedding dimension. Source data

**Extended Data Fig. 2:**
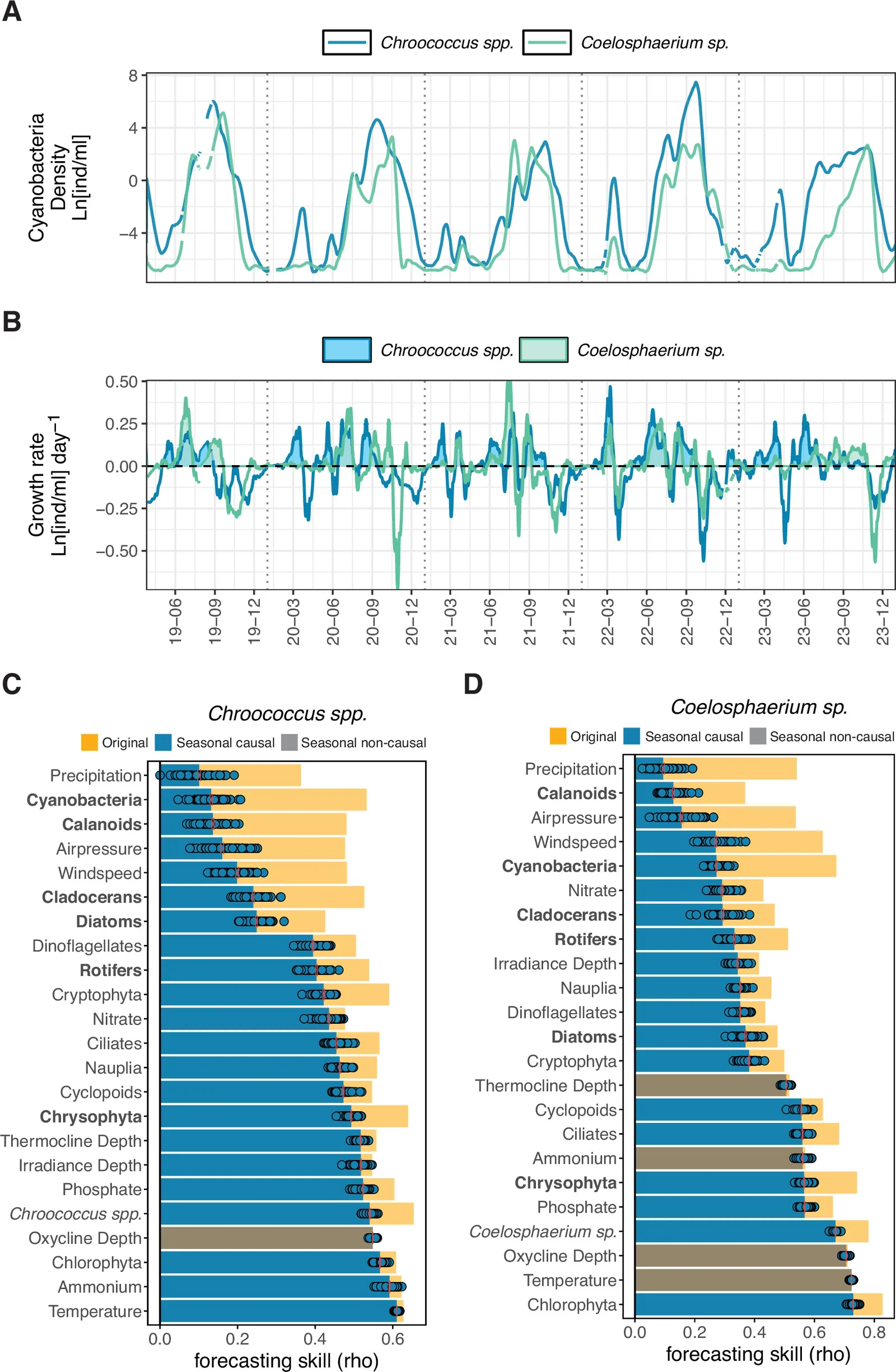
Temporal Dynamics and Biotic Coupling of *Chroococcus spp*. and *Coelosphaerium sp*. in Lake Greifensee. (**A**) Target cyanobacteria densities; (**B**) net growth rates (nGRs) calculated as the difference in logged density between two consecutive days. Convergent cross-mapping (CCM) analysis between environmental variables (biotic and abiotic) and nGR of (**C**) *Chroococcus spp*. and (**D**) *Coelosphaerium sp*. Orange bars (*original*) indicate coupling strength (convergence skill rho) between observed and predicted values from the original time series. Blue points show the individual coupling strengths obtained from 100 seasonal surrogate time series. Blue and grey bars show the mean strength using the seasonal surrogate time series, and red bars show the standard error estimate of the mean (n = 100 seasonal surrogate time series; see Materials and Methods). Blue bars (*seasonal causal*) indicate significant causal links (≥95% of surrogate rho < original rho), while grey bars (*seasonal non-causal*) indicate non-significant links (>5% of surrogate rho ≥ original rho). Biotic variables used for interaction analyses are highlighted in bold. High CCM rho values for oxycline depth, temperature, irradiance, thermocline depth, ammonium (NH_4_^+^), and phosphate (PO_4_^3−^), combined with similar heights of orange and grey bars, indicate strong seasonal coupling rather than daily-scale interactions for these variables. Source data

Abiotic factors (such as NO_3_^−^, NH_4_^+^, PO_4_^3−^, water temperature, oxycline, thermocline and irradiance depth) measured in the upper water column (1–8 m) have a high CCM *ρ*, indicating a strong coupling with cyanobacterial nGRs. Still, their predictive skill is comparable to that of the seasonal surrogates, indicating no substantial difference between the two (Fig. 1e,f). This suggests that their effects on cyanobacterial nGR represent seasonal forcing, as expected from the prominent seasonal fluctuations of the abiotic environment (Fig. 1a). The only abiotic variables that rank high in CCM *ρ* after controlling for seasonal effects are the meteorological conditions (precipitation, wind speed and air pressure), commonly used for modelling cyanobacterial blooms^10^ (Fig. 1e,f). On the other hand, biotic factors, including the density of chrysophytes, diatoms, rotifers, calanoid copepods, cladocerans and overall cyanobacterial abundances, deviate substantially from the seasonal surrogates (Fig. 1e,f). Biotic factors are strongly coupled at the daily scale with changes in cyanobacterial nGRs. This pattern is robust to varying levels of embedding dimensions (Extended Data Fig. 1) and can be observed in two more groups of cyanobacteria from Lake Greifensee—*Chroococcus* spp. and *Coelosphaerium* sp.—which are non-toxic (Extended Data Fig. 2). This suggests that biotic factors are more important than abiotic environmental conditions in explaining bloom dynamics on a daily scale.

### Net-growth rate modelling

After identifying the biotic factors that are more strongly coupled to cyanobacterial nGRs, we ask how the abundance of zooplankton and phytoplankton taxa shape nGR patterns when interacting with seasonal drivers like dissolved inorganic phosphorus (PO_4_^3−^) and water temperature. To investigate this, we analyse the dependence of the nGR of toxic *Microcystaceae* and *Dolichospermum* on the daily time series of 23 explanatory variables (environmental parameters and plankton densities) using the multi-view distance regularized S-map method (MDR S-map)^30,39,40^ (Fig. 1a–d and Supplementary Fig. 4). All explanatory variables were set at a one-day lag relative to the nGR to focus on the biologically relevant scale (day) at which growth (cell division) and loss (grazing, sinking) occur^41^. We use a multi-view distance approach to address the high dimensionality^40^, elastic-net regularization to mitigate collinearity and overfitting^39^, and test 125 S-map models with varying parameters (theta, alpha, lambda) to select the best fit based on sequential leave-future-out cross-validation (Methods).

**Fig. 2:**
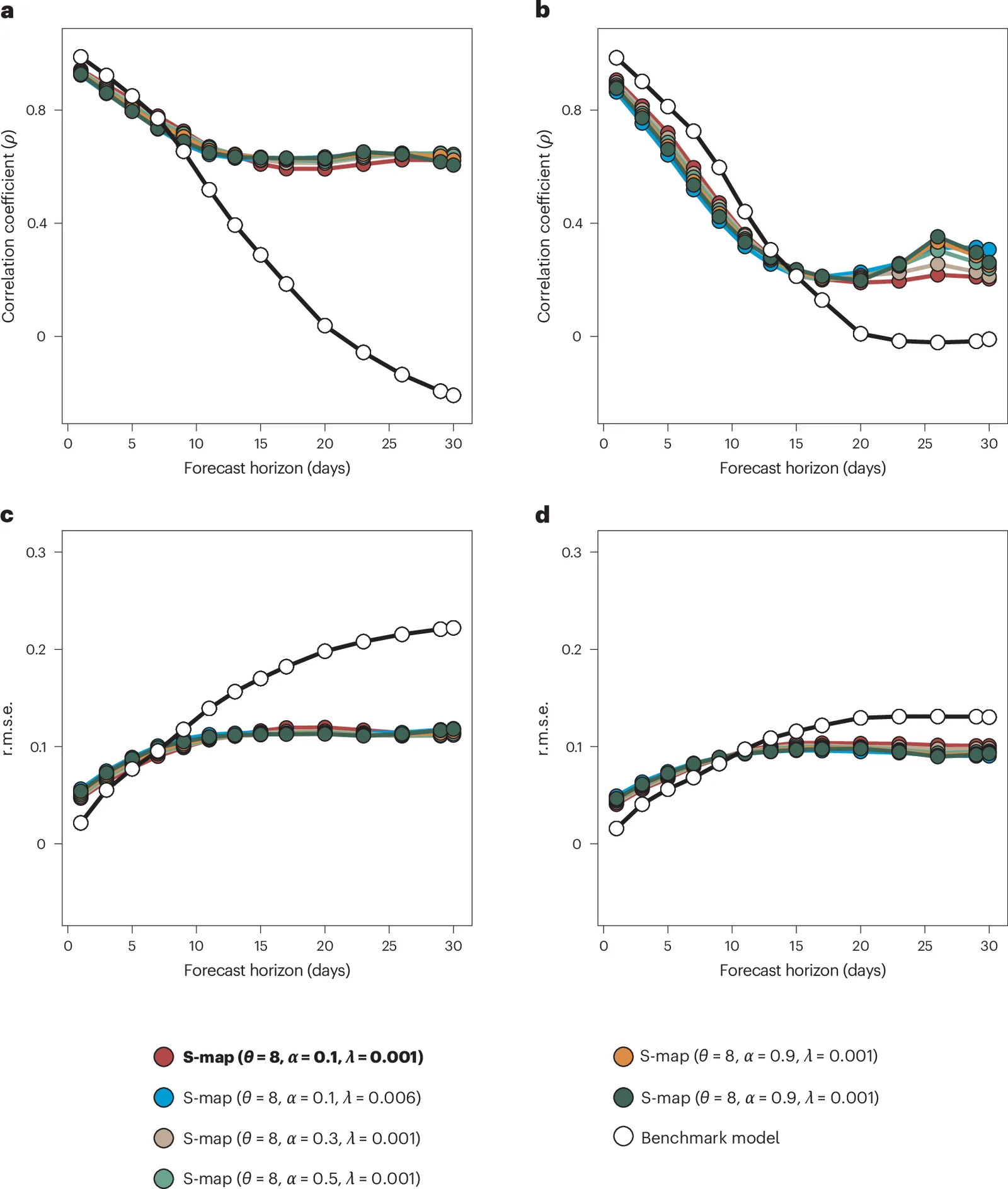
Forecast proficiency of MDR S-maps for toxic *Microcystaceae* and *Dolichospermum* spp. nGRs. **a**–**d**, Predictive performance of S-map models evaluated across increasing forecast horizons (1–30 days). The 5% best-performing parameter sets (*n* = 6), selected via cross-validation at a 1-day horizon, are used for evaluation. Performance is quantified using Pearson’s correlation coefficient (*ρ*) (for toxic *Microcystaceae* (**a**) and *Dolichospermum* spp. (**b**)) and r.m.s.e. (for toxic *Microcystaceae* (**c**) and *Dolichospermum* spp. (**d**)) between observed and predicted values. Coloured lines represent individual S-map models with distinct parameter combinations; the best-performing model (used for interaction analysis) is highlighted in bold. The black line shows the performance of a benchmark persistence model (future values remain unchanged; Methods). S-map models consistently outperform the persistence model across extended forecast horizons, even when temporal autocorrelation is excluded (Extended Data Figs. 3 and 4), indicating the successful capture of underlying system dynamics. Source data

**Fig. 3:**
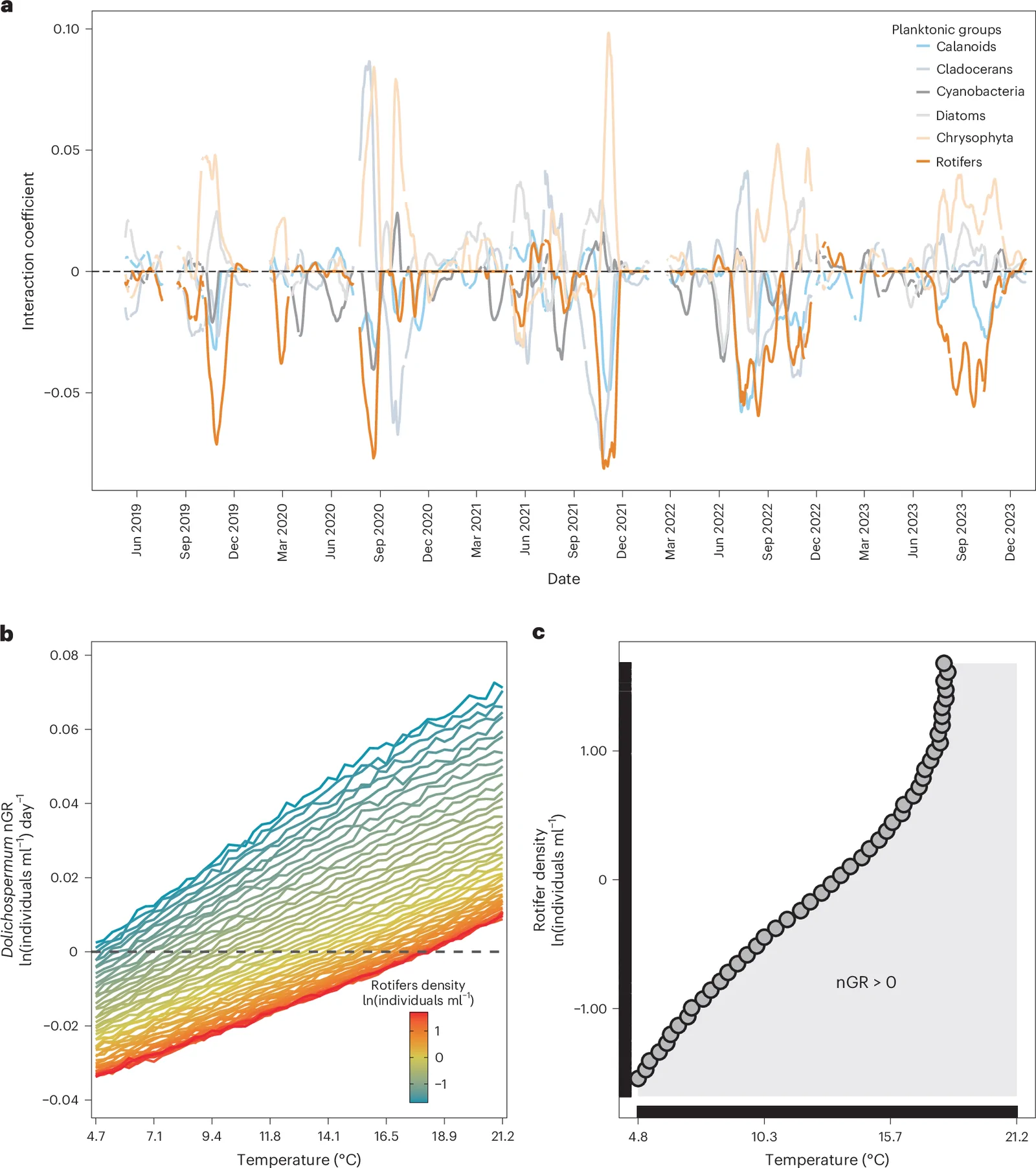
Time-varying biotic interactions and their effects on cyanobacterial blooming. **a**, Temporal dynamics of pairwise interactions between *Dolichospermum* spp. and six key planktonic groups (calanoids, cladocerans, rotifers, cyanobacteria, diatoms, chrysophyta). Interaction coefficients are derived from MDR S-map modelling of *Dolichospermum* nGR (Methods). These biotic variables show high predictive skill (*ρ*) in CCM analysis after seasonal-effect removal (Fig. 1f). Positive coefficients indicate growth facilitation, while negative coefficients suggest grazing pressure (for example, rotifers) or competition. **b**, Partial dependence analysis showing *Dolichospermum* nGR as a function of water temperature (°C) and rotifer scaled density. Scale bars indicate rotifer density in ln(individuals ml^−1^). The grey dashed line represents zero net growth (nGR = 0); values above this line indicate a positive nGR and biomass accumulation (blooming conditions). **c**, Zero net growth analysis showing temperature–rotifer-density combinations at which *Dolichospermum* transitions from negative to positive nGR. The positive relationship demonstrates that higher rotifer densities require correspondingly high water temperatures for *Dolichospermum* to achieve blooming conditions. Grey shading indicates conditions supporting positive nGR (blooming). Tick marks on axes indicate the empirical density of temperature and scaled rotifer densities in Lake Greifensee (2019–2023). Source data

**Extended Data Fig. 3:**
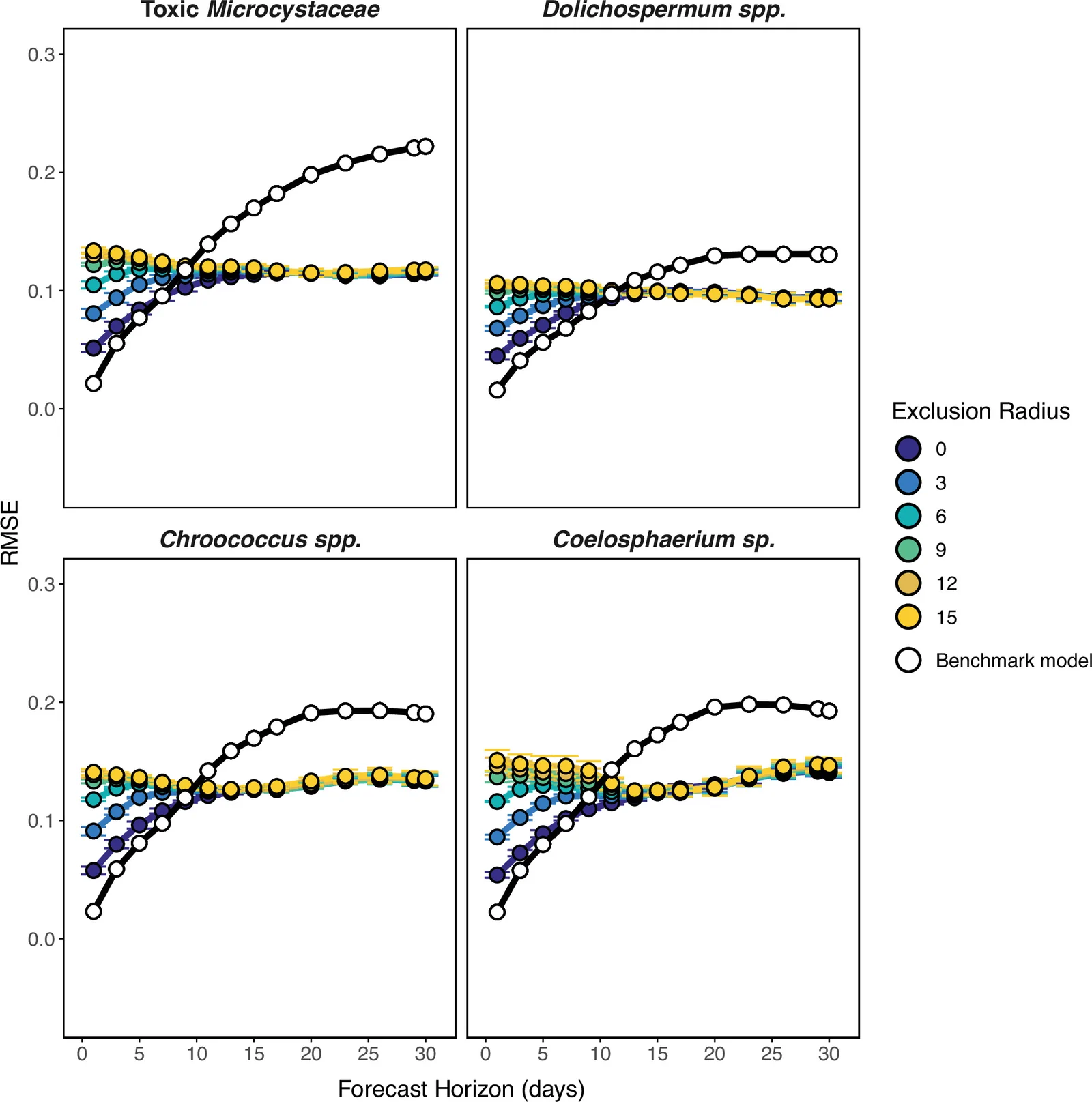
Forecasting error of MDR S-maps for cyanobacterial net growth rates. The predictive accuracy of S-map models was assessed across increasing forecast horizons (TP) and exclusion radii (ER), where ER was defined as TP+n (n = {0,15}) to account for autocorrelation effects, for each target cyanobacterial group. The 5% best-performing parameter sets (n = 6), selected via sequential leave-future-out cross-validation at a 1-day horizon, were used for evaluation. Model performance was quantified as the root mean squared error (RMSE) between observed and predicted values across forecast horizons from 1 to 30 days. Coloured points and lines represent the mean RMSE of the best-performing S-map models (n = 6) and error bars show the standard deviation. The black line represents the performance of a benchmark persistence model, which assumes that future values remain unchanged (see Materials and Methods for details). Notably, the S-map models successfully capture the underlying system dynamics beyond autocorrelation effects, as their predictive performance remains stable even at a 30-day forecast horizon. This robustness is further supported by the predictive capacity indicated by performance metrics such as Pearson’s correlation coefficient, rho (see Extended Data Fig. 4). Source data

**Extended Data Fig. 4:**
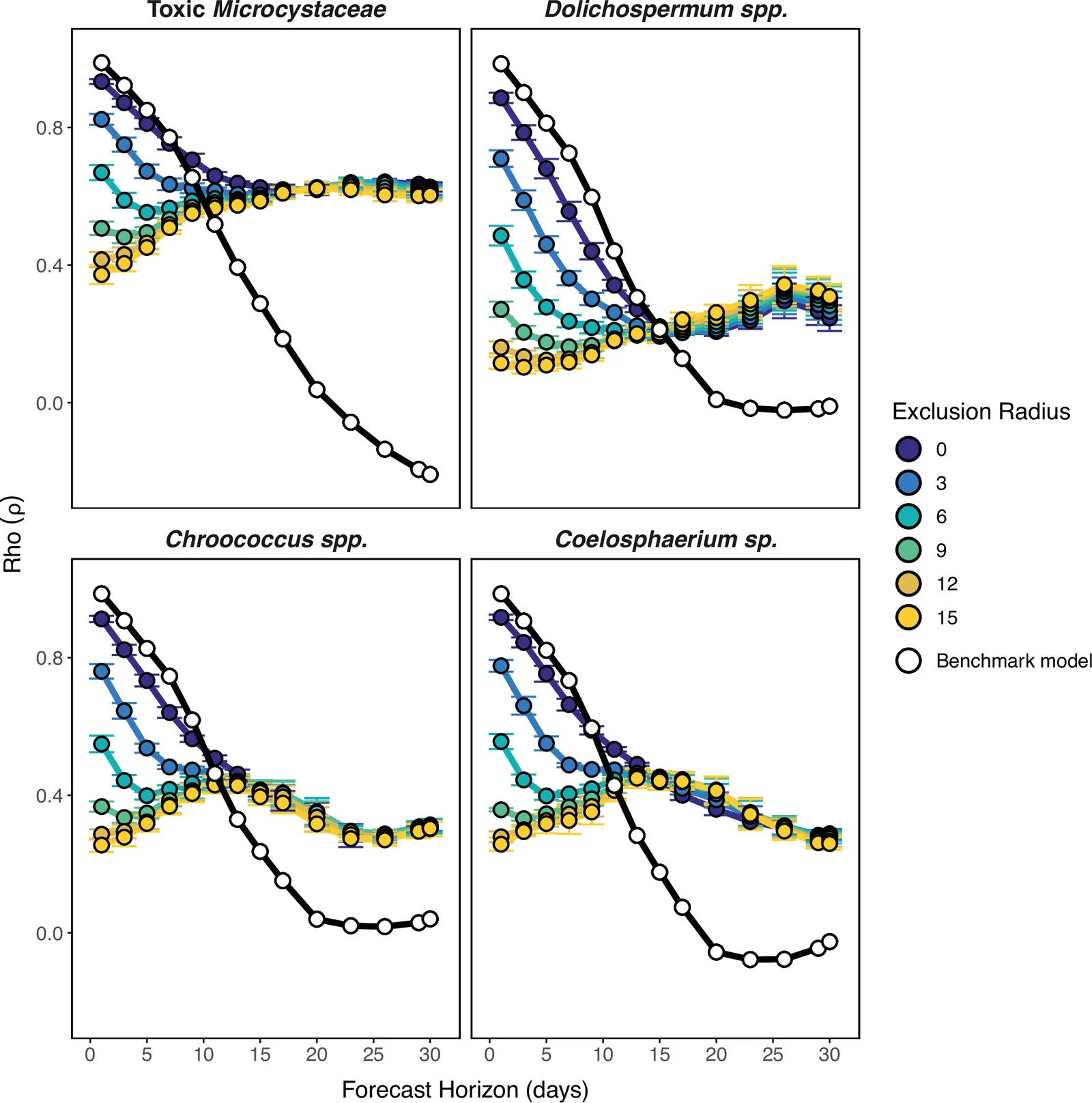
Forecasting skill of MDR S-maps for cyanobacterial net growth rates. The predictive accuracy of S-map models was assessed across increasing forecast horizons (TP) and exclusion radii (ER), where ER was defined as TP+n (n = {0,15}) to account for autocorrelation effects, for each target cyanobacterial group. The 5% best-performing parameter sets (n = 6), selected via sequential leave-future-out cross-validation at a 1-day horizon, were used for evaluation. Model performance was quantified as Pearson’s correlation coefficient rho (ρ) between observed and predicted values across forecast horizons from 1 to 30 days. Coloured points and lines represent the mean ρ of the best-performing S-map models (n = 6) and error bars show the standard deviation. The black line represents the performance of a benchmark persistence model, which assumes that future values remain unchanged (see Materials and Methods for details). Notably, the S-map models successfully capture the underlying system dynamics beyond autocorrelation effects, as their predictive performance remains stable even at a 30-day forecast horizon. Source data

S-map models reveal strong non-linearity (high theta parameter) as a defining feature in all six best models describing cyanobacterial nGRs (Fig. 2, Extended Data Table 1 and Supplementary Fig. 6). Our forecasting models outperform constant-rate baseline models over a 30-day horizon (Fig. 2) and maintain forecasting accuracy, even after controlling for temporal autocorrelation (Extended Data Figs. 3 and 4). Prediction accuracy exceeds baseline performance from day 7 to day 15 (Fig. 2) in our target taxa and increases only by 2–4 days in the most conservative autocorrelation controls across all taxa (Extended Data Figs. 3 and 4). This demonstrates that our models successfully capture temporal processes driving cyanobacterial nGR by incorporating key time-varying biotic and abiotic factors. The S-map coefficients for both biotic and abiotic factors show significant temporal variation, further confirming the dynamic nature of these interactions (Fig. 3a and Supplementary Figs. 6–9). We consistently observe negative density dependence in cyanobacterial nGR, while environmental factors exhibit taxon-specific non-linear effects (Supplementary Figs. 6–9). These findings reveal that cyanobacterial bloom formation results from complex, dynamic interaction networks that shift across environmental gradients^17,25,40,42^—a critical insight for predicting blooms under climate change and eutrophication scenarios.

**Fig. 4:**
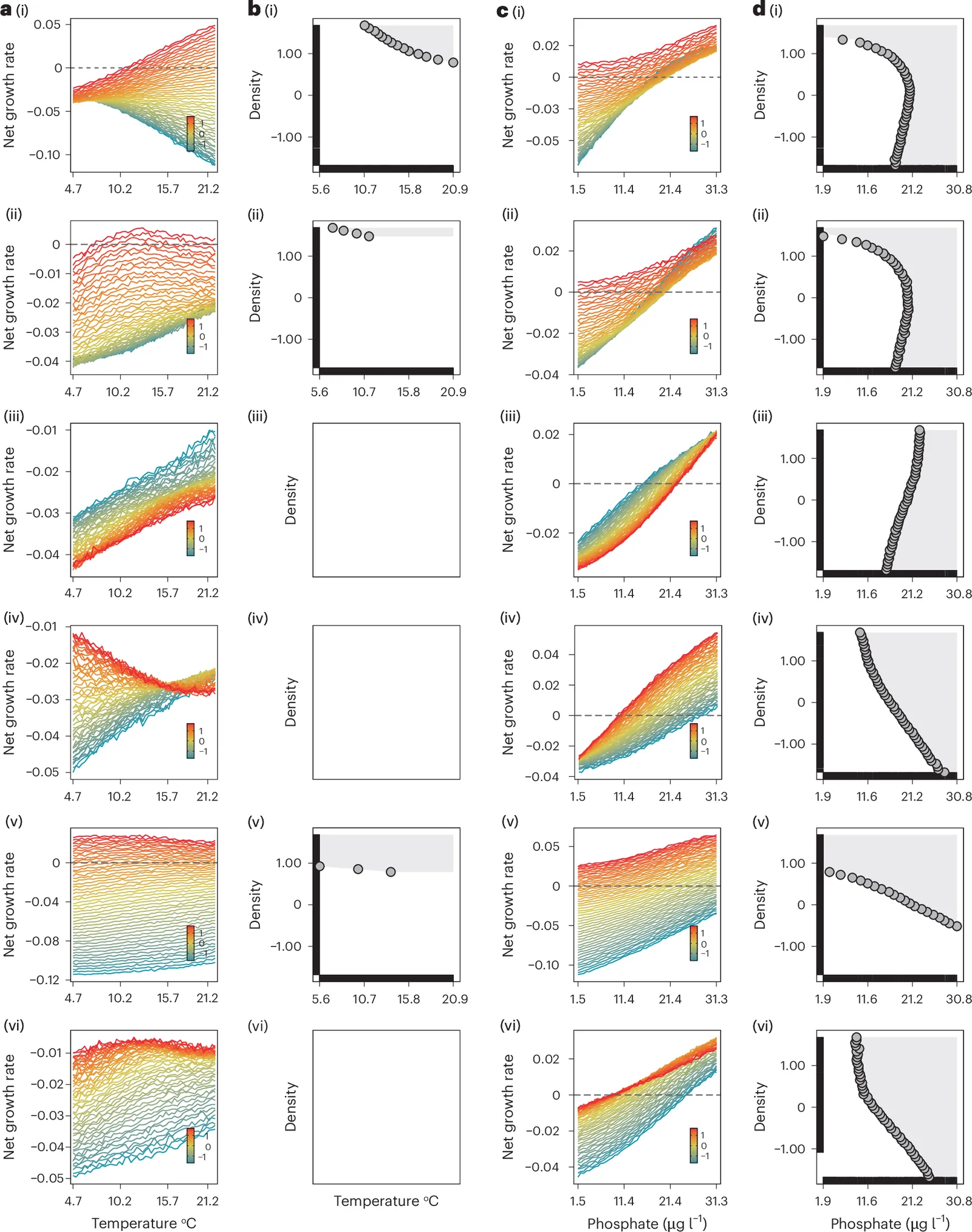
Biotic interactions shape toxic *Microcystaceae* blooming across temperature and phosphate gradients. **a**,**c**, Temperature (°C) dependency (**a**) and phosphate (PO_4_^3−^, μg l^−1^) dependency (**c**) of toxic *Microcystaceae* nGR (ln(individuals ml^−1^) d^−1^) across increasing densities (ln(individuals ml^−1^)) of key planktonic groups: cladocerans (i), calanoids (ii), rotifers (iii), diatoms (iv), chrysophytes (v) and other cyanobacteria (iv). Lines show mean estimated responses from 50 subsamples (Methods), and scale bars indicate the scaled density of the biotic factor in ln(individuals ml^−1^). **b**,**d**, Zero net growth analysis showing blooming conditions along temperature (**b**) and phosphate (**d**) gradients as influenced by increasing densities (ln(individuals ml^−1^)) of interacting organisms for key planktonic groups: cladocerans (i), calanoids (ii), rotifers (iii), diatoms (iv), chrysophytes (v) and other cyanobacteria (vi). Grey shading indicates conditions supporting positive nGR (blooming). Tick marks on axes show empirical distributions of temperature, phosphate and planktonic group densities in Lake Greifensee (2019–2023). All target group densities are scaled to enable comparison across groups with different absolute abundances. The relationship between conditions for positive net growth and organism density reveals either bloom inhibition (positive correlation indicates higher temperature and phosphate requirements for blooming as organism density increases) or bloom facilitation (negative correlation indicates lower temperature and phosphate requirements as organism density increases). White boxes in **b** (iii), **b** (iv) and **b** (vi) indicate cases where zero net growth analysis was not possible because toxic *Microcystaceae* had negative nGR across all tested conditions (see *y*-axis scale in **a** (iii), **a** (iv) and **a** (vi)). Detailed pairwise interaction analyses are provided in Supplementary Figs. 10 and 14. Source data

**Fig. 5:**
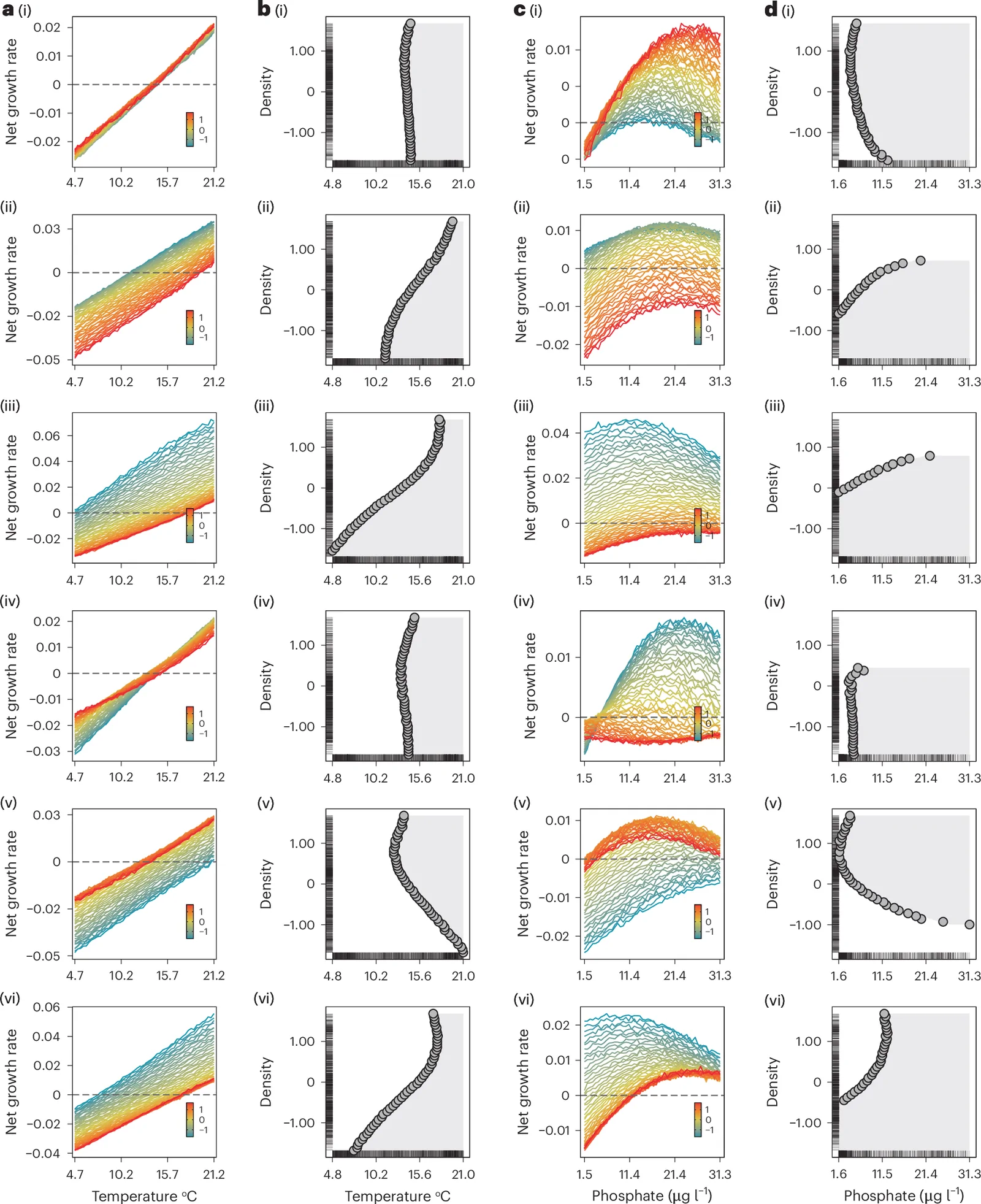
Biotic interactions shape *Dolichospermum* spp. blooming across temperature and phosphate gradients. **a**,**c**, Temperature (°C) dependency (**a**) and phosphate (PO_4_^3−^, μg l^−1^) dependency (**c**) of *Dolichospermum* spp. nGR (ln(individuals ml^−1^) d^−1^) across increasing densities (ln(individuals ml^−1^)) of key planktonic groups: cladocerans (i), calanoids (ii), rotifers (iii), diatoms (iv), chrysophytes (v) and other cyanobacteria (vi). Lines show mean estimated responses from 50 subsamples (Methods), and scale bars indicate the scaled density of the biotic factor in ln(individuals ml^−1^). **b**,**d**, Zero net growth analysis showing blooming conditions along temperature (**b**) and phosphate (**d**) gradients as influenced by increasing densities (ln(individuals ml^−1^)) of interacting organisms for key planktonic groups: cladocerans (i), calanoids (ii), rotifers (iii), diatoms (iv), chrysophytes (v) and other cyanobacteria (vi). Grey shading indicates conditions supporting positive nGR (blooming). Tick marks on axes show empirical distributions of temperature, phosphate and planktonic group densities in Lake Greifensee (2019–2023). All target group densities are scaled to enable comparison across groups with different absolute abundances. See Fig. 4 for a detailed interpretation of the relationships between conditions for positive net growth and organism densities. Detailed pairwise interaction analyses are provided in Supplementary Figs. 11 and 15. Source data

**Extended Data Fig. 5:**
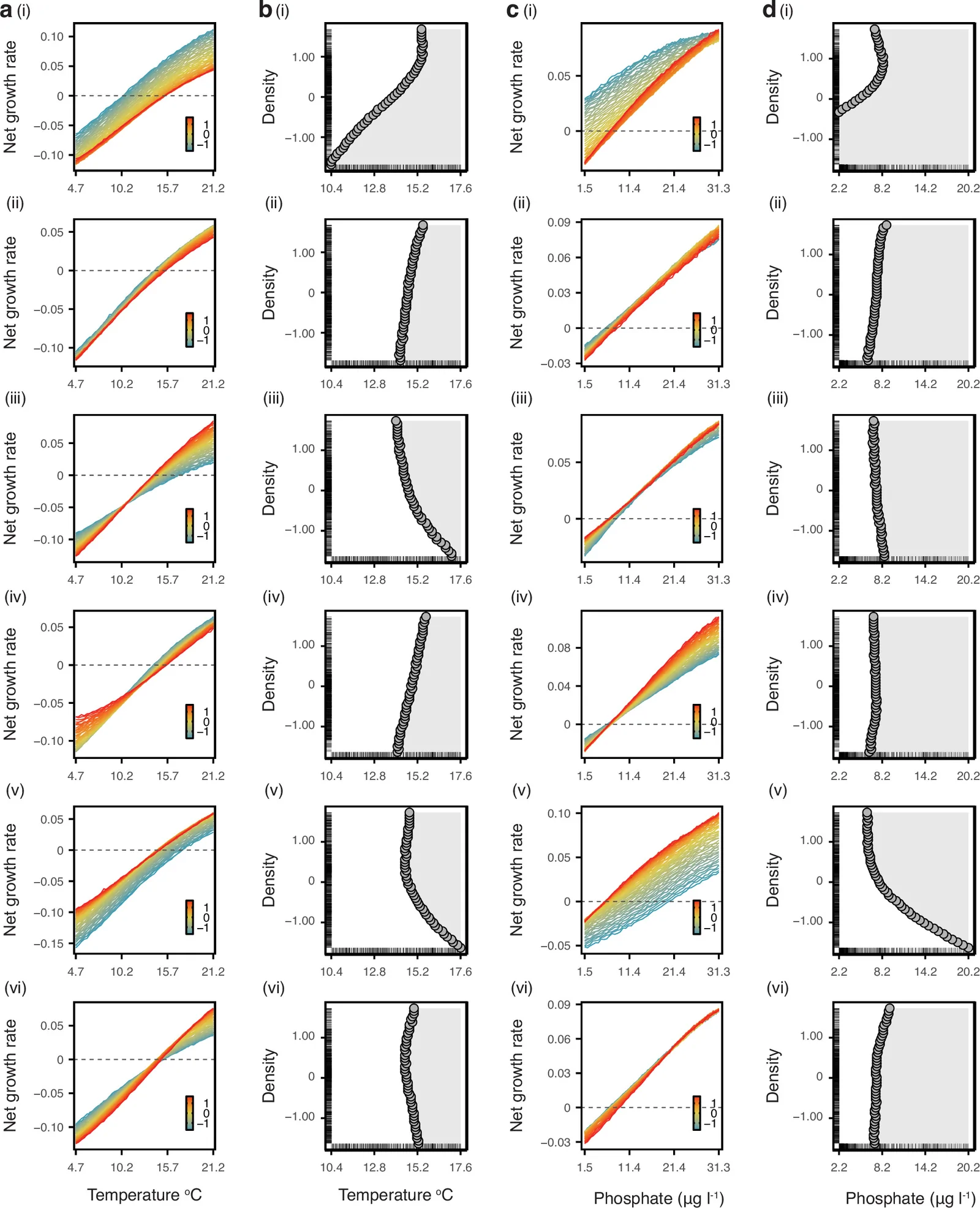
Biotic interactions shape *Chroococcus spp*. blooming across temperature and phosphate gradients. **a**,c, Temperature (°C) dependency (**a**) and phosphate (PO_4_^3−^, μg l^−1^) dependency (**c**) of *Chroococcus spp*. nGR (ln(individuals ml^−1^) d^−1^) across increasing densities (ln(individuals ml^−1^)) of key planktonic groups: cladocerans (i), calanoids (ii), rotifers (iii), diatoms (iv), chrysophytes (v) and other cyanobacteria (iv). Lines show mean estimated responses from 50 subsamples (Methods), and scale bars indicate the scaled density of the biotic factor in ln(individuals ml^−1^). **b**,**d**, Zero net growth analysis showing blooming conditions along temperature (b) and phosphate (d) gradients as influenced by increasing densities (ln(individuals ml^−1^)) of interacting organisms for key planktonic groups: cladocerans (i), calanoids (ii), rotifers (iii), diatoms (iv), chrysophytes (v) and other cyanobacteria (vi). Grey shading indicates conditions supporting positive nGR (blooming). Tick marks on axes show empirical distributions of temperature, phosphate and planktonic group densities in Lake Greifensee (2019–2023). All target group densities are scaled to enable comparison across groups with different absolute abundances. See Fig. 4 for a detailed interpretation of the relationships between conditions for positive net growth and organism densities. Detailed pairwise interaction analyses are provided in Supplementary Figs. S12 and S42. Source data

**Extended Data Fig. 6:**
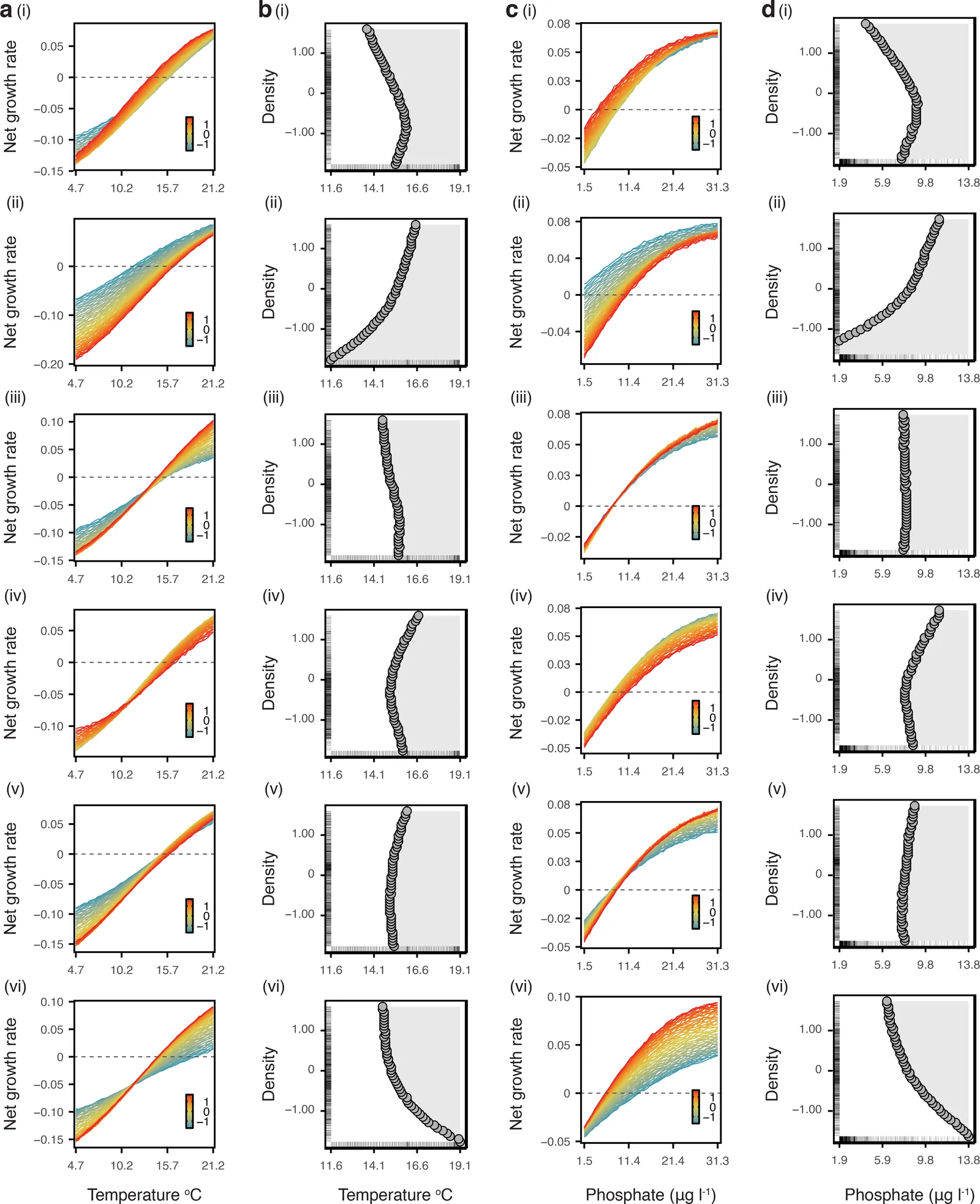
Biotic interactions shape *Coelosphaerium sp*. blooming across temperature and phosphate gradients. **a**,**c**, Temperature (°C) dependency (**a**) and phosphate (PO_4_^3−^, μgl^−1^) dependency (**c**) of *Coelosphaerium sp.* nGR (ln(individuals ml^−1^) d^−1^) across increasing densities (ln(individuals ml^−1^)) of key planktonic groups: cladocerans (i), calanoids (ii), rotifers (iii), diatoms (iv), chrysophytes (v) and other cyanobacteria (iv). Lines show mean estimated responses from 50 subsamples (Methods), and scale bars indicate the scaled density of the biotic factor in (ln(individuals ml^−1^)). **b**,**d**, Zero net growth analysis showing blooming conditions along temperature (**b**) and phosphate (**d**) gradients as influenced by increasing densities ln(individuals ml^−1^) of interacting organisms for key planktonic groups: cladocerans (i), calanoids (ii), rotifers (iii), diatoms (iv), chrysophytes (v) and other cyanobacteria (vi). Grey shading indicates conditions supporting positive nGR (blooming). Tick marks on axes show empirical distributions of temperature, phosphate and planktonic group densities in Lake Greifensee (2019–2023). All target group densities are scaled to enable comparison across groups with different absolute abundances. See Fig. 4 for a detailed interpretation of the relationships between conditions for positive net growth and organism densities. Detailed pairwise interaction analyses are provided in Supplementary Figs. S13 and S43. Source data

Having established the dynamic nature of these interactions, we next examine how specific biotic factors modulate cyanobacterial net growth to key environmental factors. To unravel how biotic factors influence cyanobacterial nGR over gradients of water temperature and phosphate, we use MDR S-maps to predict the nGR of each focal cyanobacterium across observed conditions while holding other variables at their median values. In Fig. 3b, water temperature consistently promotes *Dolichospermum*’s nGR, while increasing rotifer density shifts the entire temperature response downward (as expected from a grazer), maintaining the same shape of the response curve but requiring higher temperatures for positive net growth. We apply this approach systematically, examining how different biotic factors—such as the abundance of zooplankton or other phytoplankton—modify cyanobacterial responses to both water temperature and phosphate gradients. While we observe the expected positive net growth responses to increasing temperature and phosphate (Figs. 4 and 5 and Extended Data Figs. 5 and 6), confirming their established role in bloom formation^3,5^, our analyses reveal how biotic interactions fundamentally alter these responses, effectively showing the environmental conditions that can trigger blooms.

Building on our analytical approach of mapping nGR response patterns across environmental gradients, we further show that focal biotic factors (those deviating significantly from seasonal surrogates; Fig. 1e,f and Extended Data Fig. 2c,d) can fundamentally alter both the magnitude and shape of cyanobacterial nGR responses to temperature and phosphate (Figs. 4 and 5 and Extended Data Figs. 5 and 6). Remarkably, we observe cases where established positive nGR responses to temperature and phosphate completely reverse direction under specific biotic conditions. For instance, increasing cladoceran abundance shifts toxic *Microcystaceae*’s temperature response from negative to positive, while increasing diatom abundance causes the opposite shift (Fig. 4a(i)–(iv)). Similar biotic effects on nGR response patterns occur across phosphate gradients (Figs. 4 and 5) and all other measured abiotic conditions (ammonium, nitrate, oxycline depth, air pressure; Supplementary Figs. 10–13). While laboratory studies previously demonstrated changes in phytoplankton response curves with varying abiotic conditions^43,44^, our study provides field evidence of biotic factors reshaping these nGR patterns. These findings suggest that ignoring dynamic biotic interactions fundamentally mischaracterizes bloom formation, potentially leading to errors in understanding and predicting both the timing and magnitude of cyanobacterial blooms.

### Cyanobacterial realized niche

To assess how plankton taxa influence cyanobacteria’s ability to achieve net-positive growth (their realized niche^45,46^), we identify the temperature and phosphorus conditions for positive net growth where cyanobacteria cross the zero net growth isocline (nGR = 0) and begin accumulating biomass (Fig. 3b). We then plot these zero nGR boundaries against the abundance of biotic factors (Fig. 3c). For example, Fig. 3c shows how increasing rotifer abundance (a potential grazer) elevates the temperature requirements for *Dolichospermum* blooming. Our results reveal that both zooplankton and phytoplankton abundances significantly alter the temperature and phosphate requirements for positive nGR in both focal cyanobacteria (Figs. 4 and 5 and Extended Data Figs. 5 and 6). The magnitude of these biotic effects is substantial—shifting blooming requirements by several degrees Celsius and micrograms per litre of phosphate—especially considering Lake Greifensee’s mean annual temperature of 12.9 °C (s.d. = 5.8 °C) and mean phosphate of 9.0 μg l^−1^ (s.d. = 11.2 μg l^−1^) in the upper water column. We find consistent evidence that biotic interactions shape the realized niche of toxic *Microcystaceae* and *Dolichospermum* (Figs. 4 and 5), as well as *Chroococcus* and *Coelosphaerium* (Extended Data Figs. 5 and 6). Importantly, the effects of biotic factors on cyanobacterial realized niches vary in a taxon-specific manner across temperature and phosphate gradients (Figs. 4 and 5 and Extended Data Figs. 5 and 6). These findings highlight how species-specific biotic interactions fundamentally structure cyanobacterial ecology, suggesting that models that neglect these interactions will likely misrepresent population dynamics and fail to accurately predict the environmental conditions favouring blooms.

*Microcystaceae* thrive in eutrophic environments and warm, stable waters^6^ and are primarily favoured by increasing temperature and phosphate levels^11,47,48^. Our field observations confirm these abiotic drivers’ positive effects on toxic *Microcystaceae* accumulation (Fig. 4 and Supplementary Figs. 10 and 14). Beyond these abiotic factors, our analysis reveals complex biotic interactions significantly influencing toxic *Microcystaceae* nGR. Most zooplankton groups—including calanoids, cyclopoids, cladocerans, ciliates and copepod nauplii—enhanced toxic *Microcystaceae* nGR across water temperature and phosphate gradients, effectively shifting response curves upward and expanding the realized niche (Fig. 4 and Supplementary Fig. 10). This facilitation likely occurs through indirect mechanisms, such as selective predation on more palatable species^13^. Rotifers, however, emerged as a notable exception, consistently suppressing toxic *Microcystaceae* nGR across all temperature and phosphate levels, suggesting direct grazing pressure^15^.

Phytoplankton interactions display temperature-dependent patterns. Diatoms and chlorophytes exhibit a shift from facilitation at low temperatures to competition at high temperatures, while chrysophytes show particularly strong facilitation effects, enabling toxic *Microcystaceae* growth at phosphate concentrations as low as 3.3 μg l^−1^ (Fig. 4c(v),d(v)). Quantitatively, high densities of facilitating groups (chrysophytes, calanoids, cladocerans, cyanobacteria and diatoms) reduced the phosphate requirements for toxic *Microcystaceae* blooming, on average, by 19.68 μg l^−1^. In contrast, high rotifer densities increased phosphate requirements from 15.56 μg l^−1^ to 22.9 μg l^−1^, further confirming their inhibitory effect (Fig. 4d(iii)) (Methods). Notably, facilitation—one of the least studied biotic interactions in bloom dynamics—appears critically important for toxic *Microcystaceae* response to phosphate. We interpret these facilitation mechanisms through multiple pathways: directly via mutualistic interactions between cyanobacteria and photosynthetic protists^49^, and indirectly either by diverting grazers towards more palatable organisms (for example, diatoms and chlorophytes)^13^ or through selective mixotrophic suppression of competing protists (for example, by chrysophytes)^50^. These findings highlight how positive biotic interactions may substantially expand *Microcystis* bloom potential beyond the limitations predicted by abiotic factors alone.

In contrast to toxic *Microcystaceae*, *Dolichospermum* prefers moderate nutrient levels and shows more variable responses to environmental conditions in Greifensee^6^. Our analysis reveals that biotic interactions predominantly constrain *Dolichospermum*’s realized niche, shifting response curves downwards and inhibiting blooming (Fig. 5 and Supplementary Figs. 11 and 15)—a marked difference from the generally facilitative interactions observed with toxic *Microcystaceae*. Most plankton groups exhibit inhibitory effects on *Dolichospermum* accumulation. Rotifers demonstrate the most dramatic impact, substantially increasing environmental conditions for positive net growth by raising temperature requirements from 4.78 °C to 18.22 °C and phosphate requirements from 1.64 μg l^−1^ to 22.3 μg l^−1^ (Methods). Copepods similarly exhibit strong negative effects.

Despite this predominantly inhibitory pattern, two groups emerge as notable facilitators: cladocerans and, particularly, chrysophytes. Consistent with their effects on toxic *Microcystaceae*, chrysophytes show remarkable positive effects on *Dolichospermum*, substantially lowering conditions for positive net growth. In their presence, temperature requirements decrease from 21 °C to 12.41 °C, while phosphate requirements drop from 31.28 μg l^−1^ to 1.79 μg L^−1^ (Fig. 5b(v),d(v)). This facilitation extends beyond *Dolichospermum* and toxic *Microcystaceae*, as similar patterns were observed for *Chroococcus* (Extended Data Fig. 5). Several mechanisms may be involved in this pattern. Chrysophytes such as *Dinobryon* and *Ochromonas* are mixotrophs capable of grazing on cyanobacteria, which can induce heterocyst production and filament elongation in *Dolichospermum*—traits that enhance resistance to stress and competition^51^. They may also reduce grazing pressure indirectly—for example, when predation by generalist feeders as cladocerans (note their facilitative effect in Figs. 4a(i),c(i) and 5c(i) and Extended Data Fig. 6a(1),c(1)) targets chrysophytes rather than colony-forming cyanobacteria. The strong facilitation by chrysophytes appears particularly crucial for *Dolichospermum*, as their abundance could determine whether environmental conditions support blooming events (Fig. 5). These findings highlight chrysophytes as potential key taxa in cyanobacterial bloom dynamics, suggesting that monitoring them may significantly improve bloom forecasting models beyond traditional abiotic predictors.

## Discussion

### Implications

Understanding how biotic interactions shape species’ realized niches remains a fundamental challenge in ecology^52^. Our work provides an empirical demonstration of how realized niches emerge from species interactions—a fundamental concept applicable across ecological systems from microbes to animals and forests with significant implications for both ecological theory and management practice. Biotic interactions in plankton communities can dramatically alter blooming requirements across multiple environmental dimensions (Extended Data Table 2)—facilitative relationships may enable blooms at unexpectedly low nutrient levels, explaining occasional bloom occurrences under seemingly unfavourable conditions, while temperature conditions for positive net growth can shift by 10–13 °C, potentially advancing or extending seasonal bloom windows by months in temperate lakes. Conversely, antagonistic interactions through grazing or competition can prevent blooms entirely, even when abiotic conditions appear favourable. This evidence contributes to the understanding of long-standing paradoxes in bloom prediction failures. Critically, we identified specific planktonic groups with consistent effects across cyanobacterial taxa—particularly rotifers, copepods and cladocerans as natural bloom regulators, and chrysophytes as potential bloom facilitators—suggesting that monitoring programmes should incorporate these plankton groups as early warning indicators of cyanobacterial blooms, and that targeted food web manipulations^53,54^ could complement traditional nutrient management approaches for more effective bloom control strategies. While our empirical data come from Lake Greifensee, the fundamental mechanisms we identify—biotic modification of environmental conditions for positive net growth—represent a universal ecological process applicable to freshwater systems globally.

### Limitations

Our study reveals key biotic interactions shaping cyanobacterial nGR, though some aspects of microbial ecology remain unexplored.

First, while we capture major planktonic interactions, the roles of parasites and heterotrophic bacteria on nGRs warrant further investigation. For instance, the cyanobacterial microbiome likely influences nutrient availability and growth dynamics^55^, and viruses or fungi may significantly impact loss processes^54^. Additionally, our automated underwater microscope enables high-frequency tracking of planktonic taxa from 5–10 µm to 1 cm but excludes most picophytoplankton (<10 µm) and higher trophic levels that may indirectly influence cyanobacterial dynamics. However, the EDM framework we apply (S-maps and CCM) is designed to reconstruct system dynamics from time series even with unmeasured variables. Because our dataset spans long, continuous time series and includes more variables than the estimated optimal embedding dimension—the number of variables needed to reconstruct the underlying dynamics of the system—the reconstructed system states should implicitly capture information transfer from unobserved drivers^29^. Nevertheless, the specific identity and direct quantitative effects of these unmeasured variables on cyanobacterial nGR cannot be determined.

Second, our findings are based on data from a single lake and monitoring site. This limits the generalizability of the specific interaction patterns identified and they should be interpreted as context dependent. As expected in complex ecological systems, our findings demonstrate context dependency in cyanobacterial responses, even in one lake. While specific interaction patterns may vary across lakes and under future conditions—particularly with novel perturbations like species invasions—the mechanistic understanding of how biotic factors modify cyanobacterial niches represents fundamental knowledge. The empirical approach we developed to quantify how biotic interactions shape the conditions for positive net growth provides a framework that could be adapted to other freshwater ecosystems, recognizing that specific species interactions will vary across systems.

Third, Greifensee is a relatively small and shallow lake that is typically well mixed; however, episodic physical processes such as wind-driven mixing and short-term water movement may still influence plankton dynamics. Although we did not directly measure water circulation or wave activity, we included wind speed and thermocline depth as explanatory variables to serve as proxies for physical forcing and mixing. Future studies that integrate direct measurements of physical processes, as well as data from multiple locations, would allow explicit evaluation of spatial heterogeneity and advective transport, which are likely to be particularly important in larger and more dynamic systems. More broadly, extending EDM to explicitly incorporate spatiotemporal information represents a promising direction for future research. Recent developments in spatial EDM provide a framework for integrating spatial structure with non-linear dynamical inference and may substantially improve prediction and mechanistic understanding of harmful algal blooms across heterogeneous ecosystems^56^.

Finally, the toxic *Microcystaceae* used in our analysis include several morphologically similar and taxonomically problematic colony-forming genera (*Microcystis*, *Aphanocapsa* and *Aphanothece*). This reflects both the limitations of automated image-based identification and the long-standing taxonomic challenges of the Chroococcales order, which show overlapping morphology and high phylogenetic similarity^57^. *Aphanocapsa* or *Aphanothece* contributed to the observed and modelled nGR patterns, and this should be considered when interpreting our results, although it does not affect our general conclusions about the role of biotic interactions in shaping cyanobacterial dynamics.

## Conclusions

Climate change and eutrophication are driving increasingly frequent and severe cyanobacterial blooms, demanding improved prediction and management. Our study reveals that biotic interactions fundamentally shape bloom formation conditions—a critical insight missing from current conceptual models and forecasting approaches. Current models of cyanobacterial blooms are built on a fundamental assumption: they treat environmental conditions for positive net growth as fixed properties of species when our evidence demonstrates these boundaries are emergent properties of complex biotic interactions. By collecting and analysing high-frequency data of cyanobacteria and co-occurring plankton species, we demonstrate that these interactions significantly modify bloom requirements across environmental gradients. Neglecting these biotic effects in predictive models risks underestimating bloom expansion under climate change, particularly for toxic *Microcystaceae*, whose niche seems to expand through facilitative mechanisms.

Our findings suggest potential directions for future monitoring and management. High-frequency plankton data could complement traditional water quality measurements by revealing dynamic biotic interactions that influence bloom development. Understanding how key planktonic taxa affect cyanobacterial net growth—such as the roles we identified for rotifers and chrysophytes—may eventually inform management strategies that extend beyond nutrient control alone. However, translating these mechanistic insights into operational management tools will require further validation across diverse systems and explicit testing of the hypotheses that emerged from our study.

Beyond cyanobacterial blooms, our results validate a general ecological principle with real-world data: species’ realized niches are not fixed but emerge dynamically from biotic interactions within natural communities. Facilitation, grazing pressure and competitive interactions represent fundamental ecological processes operating across freshwater ecosystems globally. These interactions can shift the environmental boundaries where species persist, modulate the timing and intensity of population peaks, and mediate responses to changing abiotic conditions, like warming and eutrophication. This has direct relevance for understanding not only the current range of species distribution but also predicting responses to global change, as biotic interactions can expand or restrict the conditions under which species persist, affecting community composition and ecosystem function.

## Methods

### Data acquisition

#### Study system (Lake Greifensee)

We used high-frequency data collected from a monitoring platform in Lake Greifensee, Switzerland, from March 2019 to December 2023. Lake Greifensee has a surface area of 8.45 km^2^, a maximum depth of 32 m and an average depth of 18 m. Due to its relatively deep profile and temperate climate, Lake Greifensee exhibits a monomictic mixing regime, with complete water turnover occurring annually in winter. The lake is classified as eutrophic (with an average annual level of 0.04 mg l^−1^ of total phosphorus), and has a long history of nutrient pollution and toxic cyanobacterial blooms^20–22^.

#### Measured parameters

The physical parameters of the lake—water temperature, photosynthetically active radiation (PAR, wavelengths 400–700 nm) and dissolved O_2_—were measured using an Ocean Seven conductivity–temperature–dissolved oxygen probe (https://www.idronaut.it/) over the water column at 0.1-m intervals (from 1 m to 17 m) with a sampling frequency of 3–6 hours (wintertime). Weather conditions, such as maximum wind speed, precipitation and air pressure data, came from a weather station installed on the lake monitoring platform (Vaisala Oyj WXT520 and OTT HydroMet WS700-UMB) every 10 min. To assess nutrient concentrations (PO_4_^3−^, NO_3_^−^ and NH_4_^+^), we collected water samples at the monitoring station from a depth of 3 m once per week and analysed them with standard methods for nutrient chemistry^58^. Plankton community data are described in ‘Plankton data’.

### Data processing

#### Water physics

Water temperature data were restricted to the photic zone (0–8 m), above the thermocline (Supplementary Fig. 4), where plankton thrive and image acquisition occurs. Due to potential shading from the monitoring platform, we used PAR light penetration depth as a proxy for light intensity. This was the depth at which PAR reached the detection limit (5 μE m^−2^ s^−1^)^59^. Oxycline depth was defined as the depth at which dissolved oxygen reached 4 mg l^−1^. Thermocline depth was calculated using the R package rLakeAnalyzer (v.1.11.4.1). All conductivity–temperature–depth data were averaged to obtain daily values.

#### Lake meteorological conditions

We averaged measurements of maximum wind speed, precipitation and air pressure from the weather station installed on the lake monitoring platform to obtain daily estimates.

#### Water chemistry

Measurements of the inorganic nutrients nitrate (NO_3_^−^), ammonium (NH_4_^+^) and phosphate (PO_4_^3−^) were performed weekly with water samples collected at 3 m depth. To obtain nutrient levels at a daily frequency, we predicted daily nutrient values using a random forest (RF) model trained and validated for each nutrient^59^. Nutrient levels fluctuate daily, mostly due to variations in water’s physical parameters and meteorological conditions^37^. The interpolation of nutrient values based on autocorrelation of the time series would not account for such a daily variation. Therefore, we trained RF models on weekly nutrient data (*n* = 201) based on conductivity–temperature–dissolved oxygen and meteorological information from the same date, tested and validated these nutrient models on unseen nutrient values (10-times cross-validation where 80% of the data were used for training and 20% for testing), and then used RF models to predict (as opposed to interpolate) daily nutrient concentrations based on daily data of water physics and meteorological conditions (see ref. ^59^ and Supplementary Table 4 for details). RF model training, validation and testing were performed using the Caret package in R (v.6.0-94).

#### Plankton data

##### Automated image analysis and image classification

Plankton was monitored with a dual-magnification dark-field imaging microscope that captures all particles in the size range between ~10 μm and ~ 1 cm at 3-m depth using two magnification objectives: one for phytoplankton and small zooplankton (×5.0) and one for larger zooplankton (×0.5)^60,61^. The instrument is manufactured by Guatek (www.guatek.com). The choice of deployment depth in Greifensee is motivated by the average structure of the lake water column during the growing season: the thermocline is generally around 8 m (Supplementary Fig. 4) and the epilimnion is generally well mixed by daily thermal winds. The 3 m depth is a representative sample of the phytoplankton community of the photic zone^59^. Images were collected at a rate of 1 frame per second for 10 min every hour, and regions of interest (ROIs) were automatically identified by the instrument using a Canny edge detector^61^. A Python image processing script performed colour conversion, edge-detection and morphological feature extraction (for example, object dimensions) of detected raw ROIs (https://github.com/tooploox/SPCConvert). These objects were manually annotated and classified by two trained taxonomists (authors M.R. and S.E.), and served as input for the image classifier, after being resized to 224 × 224 pixels (Imagenet standardization)^62,63^.

A hierarchical classification structure was employed, consisting of two classifiers for the ×5.0 magnification: the former distinguishes between different cyanobacteria species and a background class, while the latter further classifies samples predicted to be of the background class into additional phyto- and microzooplankton classes. This improves the accuracy of cyanobacterial classification by reducing the false-positive predictions. A separate classifier was trained to distinguish images in the ×0.5 magnification. All three classifiers shared the same pre-trained BeITv2 architecture^64^ but differed in the last layer. The classifiers were trained in two stages, in the first stage only the weights of the input and output layers of the neural network are optimized. In the second stage, all weights are unfrozen and optimized. The AdamW optimizer with a weight decay of 0.01 was used, and the learning rate was scheduled with linear warmup cosine annealing^65^. A validation set (15%) was used for checkpoint selection, and the training set (70%) was augmented to improve generalization. The remaining 15% are used as a test set on which the metrics given in the following are computed (Supplementary Figs. 1–3 and Supplementary Table 3). The first classifier was trained using focal loss with *γ* = 2 and equal weight for both classes^66^ to obtain high precision on cyanobacteria classes, while categorical cross-entropy was used for the other two classifiers^66^. Test-time augmentation was used during inference to improve robustness^64^. The performance of the three classifiers can be seen in Supplementary Figs. 1–3. For further details, see https://github.com/kaechb/lit_ecology_classifier.

##### Transformation of planktonic densities

Classified object counts at the daily scale were log-transformed (base 10) and smoothed using a local polynomial regression to remove random noise, as described in ‘Smoothing of daily data’ (loess function with a span of 0.03). For the aggregation of taxa into higher taxonomic groups across different magnifications and to facilitate comparison, log_10_-transformed ROIs were converted to log_10_ individuals ml^−1^ using calibration functions described in ref. ^61^ (for the x5.0 magnification, log(individuals ml^−1^) = −2.9 + 0.71 log(ROIs); for the x0.5 magnification, log(individuals ml^−1^) = −0.91 + 1.3 log(ROIs)). Following unit transformation, densities were back-transformed (anti-logarithm), and taxa were aggregated within and/or across magnifications to create higher taxonomic groups for our analysis (see ‘Target taxa and planktonic groups’). Finally, these groups were transformed with the natural logarithm for further analysis (for example, estimation of nGRs). A total of 1,742 data points representing taxon and group-specific density estimations were collected over five years (after removing days when the underwater microscope was serviced or there were not enough images), spanning March 2019–December 2023.

##### Target taxa and planktonic groups

Our target cyanobacteria included two potentially toxin-producing taxa—the toxic *Microcystaceae* group (including *Microcystis* spp., *Aphanothece* spp., *Aphanocapsa* spp.) and *Dolichospermum* spp.—and two non-toxic taxa—*Chroococcus* spp. and *Coelosphaerium* sp. The absolute and relative abundances of the genera included in the toxic *Microcystaceae* group is presented in Supplementary Fig. 16. To assess the influence of biotic interactions on cyanobacteria, we aggregated zooplankton and phytoplankton taxa into broad taxonomic groups. From the 34 classified zooplankton taxa, we grouped taxa according to their phylogenetic class into six categories (Supplementary Table 1), capturing distinct zooplankton feeding behaviour. Similarly, the 49 identified phytoplankton taxa were aggregated to the phylum level, resulting in six taxonomic groups (Supplementary Table 2).

#### Smoothing of daily data

The daily data of all variables used in this study were smoothed using a local polynomial regression (loess in Stats, v.3.6.2) function with a span of 0.03 to remove serially uncorrelated (random) noise. The chosen span was verified by examining the autocorrelation in the residuals after smoothing to ensure no removal of deterministic signals from the time series. Supplementary Figs. 17 and 18 show the distribution of residuals after smoothing, and Supplementary Figs. 19 and 20 show their partial autocorrelation function. To explicitly test whether smoothing could bias the inference of interactions, we conducted additional sensitivity analyses using a chaotic three-species model with added measurement noise. We compared model performance and inferred interaction coefficients obtained from smoothed and unsmoothed time series across multiple noise levels. These analyses demonstrate that smoothing reduces measurement noise without altering the underlying deterministic interactions. Full details of the simulation framework and results are provided in the Supplementary Methods and Supplementary Figs. 21–25.

### Data analysis

All analyses and figure production were conducted in R version 4.4.1.

#### Convergent cross-mapping

To assess the strength of coupling between environmental variables and cyanobacterial nGR, we tested for direct links between them using CCM^29^. We applied CCM to test the strength of causal links between environmental variables and cyanobacterial nGR, estimating the forecast skill (*ρ*, Pearson’s correlation coefficient between observations and predictions; ‘Forecasting proficiency of nGR models’) at prediction horizon TP = −1 to focus on direct interactions. To factor out potential seasonal influences on the strength of causal links, we conducted CCM on 100 seasonal surrogates. These surrogates were created by randomly substituting values within each calendar week of the target year with values from the same week in other years (4 or 5 years depending on the chosen month). We applied CCM to both original and seasonal surrogate time series and assessed convergence of *ρ* with increasing library size, following the original CCM framework^29^. Specifically, for each variable pair we estimated *ρ* at a minimum (10%) and maximum (80%) library size using 100 random subsamples and tested whether mean *ρ* increased with library size (Supplementary Fig. 26). Finally, to identify variables exerting causal effects beyond seasonal structure, we compared *ρ* values at maximum library size (80%) between the original and seasonal surrogate series. A variable was considered causally linked at the daily scale when the CCM *ρ* from the original time series exceeded the corresponding surrogate *ρ* in at least 95 out of 100 surrogate comparisons. The three zooplankton and phytoplankton groups that were the strongest coupled to the target cyanobacterial nGRs were selected for further analyses (Fig. 1e,f and Extended Data Fig. 2c,d). The CCM function was used with an embedding dimension (E) of 10 (similar to the S-maps see ‘nGR modelling with S-maps’) and a prediction horizon of −1 (rEDM, v.1.15.4). The CCM results vary only slightly depending on the number of E (Supplementary Figs. 27–29), with a clearer distinction between causal interactions and seasonally driven associations at higher E (Supplementary Figs. 28–31 and Extended Data Fig. 1).

#### nGR modelling with S-maps

We modelled the nGR of the four target cyanobacterial taxa using the MDR S-map method^17,30,39,40^. S-maps belong to the EDM framework and were chosen because of their capacity to infer interactions in highly non-linear, high-dimensional and noisy data describing natural plankton communities. S-maps are locally linear multiple regression models, where data points are assigned varying weights based on their position in state-space. State-space dependence allows interactions between variables to change over time as the system changes states, avoiding the assumption of stationary interactions. The interactions between the target and explanatory variables are estimated through the coefficients of the S-maps model at each time point. All explanatory variables were scaled (with the scale function in R) before fitting regularized MDR S-maps (rEDM, v.0.4.7).

#### Dealing with high dimensionality

For modelling, we used 23 explanatory variables (or embedding dimensions), including 10 ecologically relevant physical, chemical and meteorological parameters and 13 biotic variables representing the densities of observed planktonic groups that could potentially interact with cyanobacteria (6 zooplankton, 6 phytoplankton groups and the density of each target taxon). The full set of explanatory variables is also presented in Fig. 1e,f, Supplementary Fig. 4 and in sections ‘Water physics’–’Plankton data’ of Methods.

To mitigate the challenges posed by high dimensionality and avoid inaccuracies in nearest-neighbour distance calculations within state-space, we employed a multi-view approach^30,40^ to generate weighted average distance estimates from the top 100 out of 1,000 multi-view embeddings. Each multi-view embedding was constructed by randomly selecting 10 variables from the available 23. To evaluate these 1,000 multi-view embeddings, we utilized simplex projections with leave-one-out cross-validation and an exclusion radius (ER) of 14 points. The 100 configurations that yielded the lowest root mean squared error (r.m.s.e.) in forecast skill were selected and the highest-performing multi-view embeddings were assigned greater weight in the distance calculation^30^. Distances between points in state-space were computed using Euclidean distance with the dist function in R (vegan, v.2.6-8). In addition, to address potential issues arising from the use of numerous explanatory variables (collinearity) and noise in the data, which could compromise our estimates of interaction coefficients and lead to overfitting, we implemented elastic-net regularization^39^. This technique penalizes the model coefficients and improves the generalization of the model (model selection). Elastic-net regularization was applied using the glmnet function in R (glmnet, v.4.1-8).

#### Parameter selection and model validation

The regularized multi-view distance S-maps require the a priori selection of three important tuning parameters—theta, alpha and lambda. Theta is a parameter within S-maps that controls the weights assigned to data points during local linear regression fitting. Alpha and lambda are parameters of the elastic-net regularization function. Alpha regulates the balance between the L1 (Lasso) and L2 (Ridge) penalties (*α* = 1 corresponds to Lasso, and *α* = 0 corresponds to Ridge), while lambda controls the overall strength of the penalty.

To select the optimal combination of these tuning parameters, we tested all possible combinations of the following values: theta (0, 0.1, 1, 3, 8), alpha (0.1, 0.3, 0.5, 0.7, 0.9) and lambda (1.000, 0.178, 0.032, 0.006, 0.001), resulting in 125 models (Supplementary Fig. 5). The predictive performance of each model was evaluated with a sequential leave-future-out cross-validation, whereby starting with an initial library size of 10%, we predicted one time point into the future and progressively added the predicted point to the training set, continuing this process until the end of the time series. The best model parameters were the ones with the lowest r.m.s.e. in forecast skill one-day forward (Extended Data Table 1).

We performed a sensitivity analysis to evaluate how temporal autocorrelation might influence parameter selection. Specifically, we introduced an ER (‘Forecasting proficiency of nGR models’) to progressively remove serially correlated data points at a forecast horizon of a day (TP = 1) and repeated the model selection procedure across all 125 parameter combinations for each cyanobacterial nGR (Supplementary Fig. 32). The best-performing models (lowest r.m.s.e.) consistently cluster in the same region of parameter space, particularly around *θ* = 8, which is a crucial parameter for the S-map models. Even under stricter exclusion of autocorrelated data points (ER ≤ 9), the location of the optimal parameter region remained relatively stable, indicating that our parameter selection is robust to temporal autocorrelation within the range of acceptable model performance (‘Forecasting proficiency of nGR models’ and Extended Data Figs. 3 and 4).

#### Forecasting proficiency of nGR models

To evaluate the predictive power of our best-performing model used to study nGR responses, we compared its forecasting proficiency with that of the next five best-performing models (a total of six models, with different combinations of theta, alpha and lambda parameters, as described in ‘Parameter selection and model validation’). In this forecasting exercise, each model (of the four target cyanobacterial taxa) was used to predict the future nGR across forecast horizons (TP) ranging from 1 to 30 days using the sequential leave-future-out approach (as described in ‘Parameter selection and model validation’). At the same time, we assessed the models’ sensitivity to autocorrelation in the time series by systematically excluding an increasing number of the closest time points when generating predictions (ER). This sensitivity analysis is particularly relevant for short-term forecasts. We set ER = TP + *n*, where *n* ranged from 0 to 15 days: in this way, ER ≥ TP to prevent data leakage into the model fitting. The forecast proficiency of each model was assessed using r.m.s.e. and Pearson’s correlation coefficient *ρ*:

$${\mathrm{r.m.s.e.}} = \sqrt{\frac{1}{n}\sum_{i=1}^{n}(Y_i-\hat{Y}_i)^{2}},\rho = \left(\frac{\sum (X_i-\bar{X})(Y_i-\bar{Y})}{\sqrt{\sum (X_i-\bar{X})^{2}}\sqrt{\sum (Y_i-\bar{Y})^{2}}}\right)$$

Forecast proficiency was evaluated relative to a persistence model, which assumes that the current nGR of each cyanobacterium remains constant over the forecast horizon. In Fig. 2, we present the forecasting results for the six best-performing S-map models with *n* = 0 (ER = TP), while Extended Data Figs. 3 and 4 display the mean and standard deviation of r.m.s.e. and *ρ* of the best six models across the full range of *n* values from 0 to 15.

#### Main and interactive effects of variables on cyanobacterial nGR

To estimate the main effects of all explanatory variables, we extracted the coefficients from the MDR S-maps model fitted to the entire dataset with the optimal parameters. This resulted in 23 time series of coefficients (Supplementary Figs. 5–9) and their summarized averages (Supplementary Fig. 33), which describe how the effect of one variable changes as the system evolves and other variables vary accordingly. To estimate the interactive effects of biotic and abiotic variables on cyanobacterial nGR (multivariate partial dependence), we predicted nGRs allowing only the two interacting variables of interest (one biotic and one abiotic) to vary, while the rest were maintained at their median levels (Supplementary Figs. 10–13). For each variable of interest, we selected a grid of 50 values spanning from the minimum to the maximum observed in the dataset, excluding the 10% of extreme values^17^. This resulted in 250 combinations of biotic and abiotic values for each multivariate partial dependency. To ensure the robustness of our response function patterns, we conducted a sensitivity analysis on the length of the data used to train the model. We extracted the response functions for each of the 250 combinations of values by randomly subsampling 50 times different proportions of the initial data (10%, 20%, 40%, 60%, 70%, 90% and 95%). This additional step confirmed that the observed patterns of response functions were consistent across varying data lengths, enhancing the robustness and generality of our findings (see Supplementary Figs. 34–41 for selected examples of temperature and phosphate). In Figs. 3c, 4 and 5, Extended Data Figs. 5 and 6 and Supplementary Figs. 10–13, we report the average of the 50 resamplings using 70% of the dataset for each of the 250 combinations of biotic and abiotic values.

#### Quantifying the effect of biotic interactions on cyanobacterial blooming

To quantify the effects that biotic factors have on the cyanobacterial zero net-growth isoclines (that is, the blooming point), we focused on the conditions under which each target cyanobacterium achieves a net-positive nGR. Trained S-maps predicted the multivariate partial dependencies of cyanobacterial nGRs (‘Main and interactive effects of variables on cyanobacterial nGR’), as a function of the most common and strongly coupled biotic factors (‘Convergent cross-mapping’), and two abiotic variables—water temperature and phosphate—which are strongly but seasonally coupled to the cyanobacterial nGR and are important drivers of cyanobacterial blooms. The blooming point (intercept of the zero net growth isocline, nGR = 0) is the value of water temperature or phosphate concentration above which cyanobacteria can accumulate biomass under a specific density of the target biotic group (Fig. 3). An increase in temperature or phosphate levels required to attain a positive nGR with increasing biotic density suggests the inhibition of blooming (Fig. 3b,c). This could be interpreted as grazing pressure from zooplankton or competition by phytoplankton. Conversely, a decrease in the required temperature or phosphate level required for growth, with an increasing density of biotic factors, suggests the facilitation of blooming, regardless of the biotic group (Fig. 4c(iv),d(iv)). To compare the effect of biotic factors within and between cyanobacteria, we calculated the range of variation in the blooming point (zero net growth isocline) as the difference between its maximum and minimum estimated values over temperature and phosphate gradients for the target biotic factor (Extended Data Table 2). For the effects of all biotic factors over all the abiotic factors on the blooming point of cyanobacteria, see Supplementary Figs. 14, 25, 42 and 43.

### Reporting summary

Further information on research design is available in the Nature Portfolio Reporting Summary linked to this article.

## Supplementary information

Supplementary Information Supplementary Methods, Figs. 1–43 and Tables 1–3.

Reporting Summary

Peer Review File

## Source data

Source Data Fig. 1 Statistical source data.

Source Data Fig. 2 Statistical source data.

Source Data Fig. 3 Statistical source data.

Source Data Fig. 4 Statistical source data.

Source Data Fig. 5 Statistical source data.

Source Data Extended Data Fig. 1 Statistical source data.

Source Data Extended Data Fig. 2 Statistical source data.

Source Data Extended Data Fig. 3 Statistical source data.

Source Data Extended Data Fig. 4 Statistical source data.

Source Data Extended Data Fig. 5 Statistical source data.

Source Data Extended Data Fig. 6 Statistical source data.

Source Data Extended Data Table 1 Statistical source data.

Source Data Extended Data Table 2 Statistical source data.

## Figures and Tables

**Extended Data Table 1:**
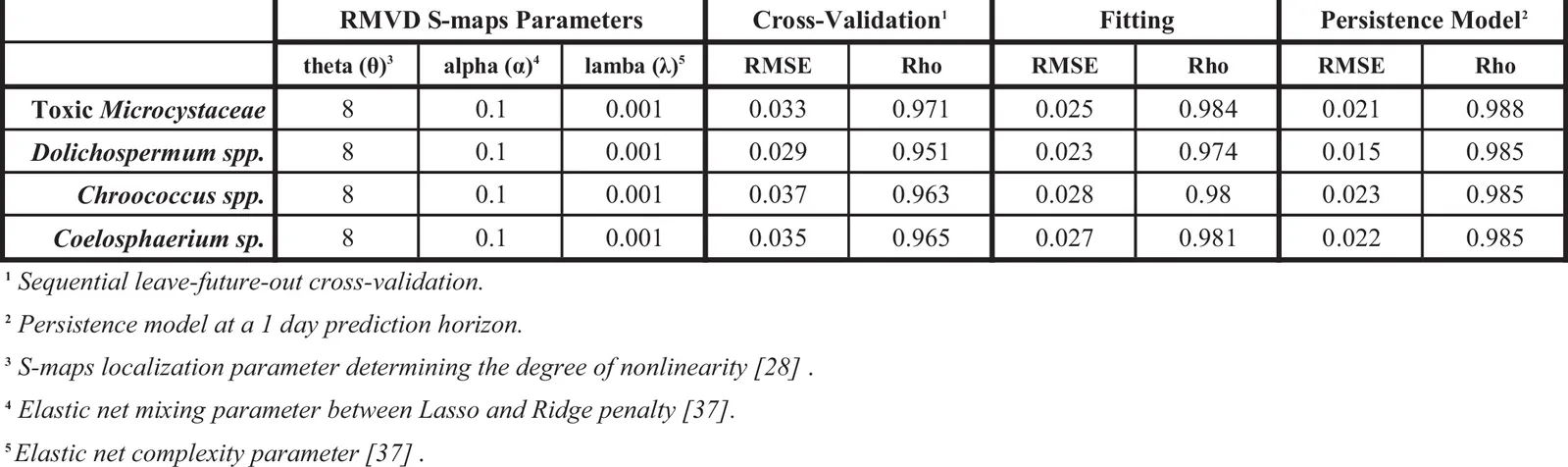
Performance of MDR S-maps from the best cross-validated parameters for each target cyanobacteria

Source data

**Extended Data Table 2:**
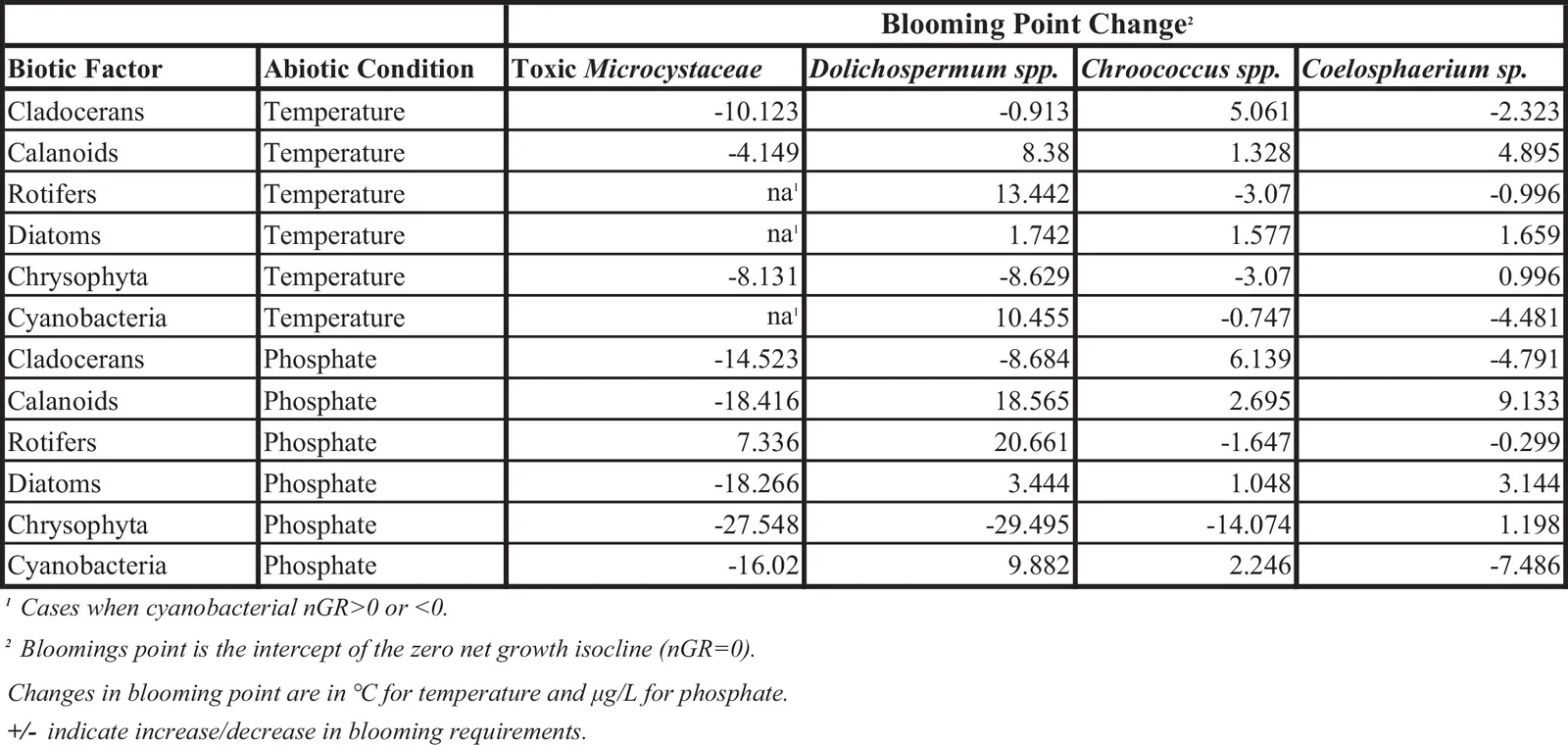
Blooming point change across gradients of water temperature or phosphorus levels for target biotic factors and cyanobacterial taxon

These changes are calculated by varying biotic factors from their minimum to their maximum observed values and taking the resulting variation in temperature (°C) and phosphate (PO_4_^3−^, μg/L) levels.

Source data

## Data Availability

The raw data supporting this study’s findings are openly available via the Eawag Research Data Institutional Repository (ERIC Open) at 10.25678/000GPG (ref. ^67^). Raw abiotic data are available via ERIC Open at 10.25678/000C8P (ref. ^68^) and raw biotic data are available at 10.25678/000C2G (ref. ^69^). Source data are provided with this paper.

## Code availability

The R scripts used to generate the results of this manuscript are available via ERIC Open at 10.25678/000GPG (ref. ^67^), together with the final aggregated dataset. All analyses have been performed in R version 4.5.1, with the use of the packages tidyverse (v.2.0.0), rEDM (v.0.4.7 and v.1.15.4), vegan (v.2.7.2) and glmnet (v.4.1.10). All figures have been generated with the use of the package ggplot2 (v.3.5.2).
