# Author Correction: Biotic interactions shape the realized niche of toxic cyanobacteria

**DOI:** 10.1038/s41559-026-03179-y

**Published:** 2026-08-24

**Authors:** Pinelopi Ntetsika, Stefanie Eyring, Ewa Merz, Marta Reyes, Benno Käch, Stephan B. Munch, Stuart R. Dennis, Marco Baity-Jesi, Francesco Pomati

**Affiliations:** 1 Department of Aquatic Ecology, Swiss Federal Institute of Aquatic Science and Technology (Eawag), Dübendorf, Switzerland; 2 Integrative Oceanography Division, Scripps Institution of Oceanography, University of California San Diego, La Jolla, CA USA; 3 Department of Applied Mathematics, University of California, Santa Cruz, CA USA; 4 Scientific IT, Swiss Federal Institute of Aquatic Science and Technology (Eawag), Dübendorf, Switzerland; 5 Department of Systems Analysis, Integrated Assessment and Modelling, Swiss Federal Institute of Aquatic Science and Technology (Eawag), Dübendorf, Switzerland

**Keywords:** Microbial ecology, Freshwater ecology, Population dynamics, Limnology

Correction to: *Nature Ecology & Evolution* https://doi.org/10.1038/s41559-026-03131-0, published online 20 July 2026.

In the version of this article initially published, in Fig. 1f, the *y*-axis label “Cryptophyta” incorrectly appeared in bold instead of the “Chrysophyta” label; in Fig. 2a, b, the current *y*-axis labels “Correlation coefficient (ρ)” appeared originally as “Coefficient (ρ)”; and in Fig. 3a, the *y*-axis label now reading “Interaction coefficient” appeared originally as “Coefficient (ρ).” In Extended Data Figs. 5 and 6, the images and legends were earlier, incorrect versions and are now updated. The figures have been amended in the HTML and PDF versions of the article.

