# Supplementary material for: Biotic interactions shape the realized niche of toxic cyanobacteria
Source: Nat Ecol Evol. 2026 Jul 20;10(8):1434–48. doi: 10.1038/s41559-026-03131-0 (PMC13441909; doi:10.1038/s41559-026-03131-0)
Supplement: Supplementary file 1 [file 41559_2026_3131_MOESM1_ESM.pdf]

nature ecology & evolution

Supplementary information

https://doi.org/10.1038/s41559-026-03131-0

Biotic interactions shape the realized niche of toxic cyanobacteria

In the format provided by the authors and unedited

Methods

1. Evaluation of smoothing on chaotic time series

To assess whether our loess smoothing procedure affects the inference of interaction coefficients in chaotic dynamics, we performed the following analyses using in-silico time series generated from the three-species Hastings–Powell model (Hastings & Powell, 1991), a canonical chaotic food chain model given by

$$\frac{dx}{dt} = ax(1 - x) - \frac{\beta_1 xy}{1+\pi_1 x}$$

$$\frac{dy}{dt} = \frac{\beta_1 xy}{1+\pi_1 x} - \frac{\beta_2 yz}{1+\pi_2 y} - \mu_1 y$$

$$\frac{dz}{dt} = \frac{\beta_2 yz}{1+\pi_2 y} - \mu_2 z$$

with parameters $a = 1, \beta_1 = 5, \pi_1 = 3, \beta_2 = 0.1, \pi_2 = 2, \mu_1 = 0.4, \mu_2 = 0.01$

1.1 Simulation of artificial time series

The Hastings–Powell model was simulated for 2000 time steps, and the first 1000 steps were discarded to remove transient dynamics, ensuring that the series reflects the stable chaotic attractor. Three target variables (x, y, z) were considered.

1.2 Noise addition based on empirical variance

We calculated the variance of each plankton abundance time series in the manuscript dataset, before and after loess smoothing (span = 0.03), to estimate the amount of excess measurement noise:

$$variance_{Fraction} = (variance_{Raw} - variance_{Smoothed}) - 1$$

We used the 25th, 50th, and 75th percentiles of this distribution as proportionality constants (p) to generate additive Gaussian noise in the simulated time series (**Fig. S21**):

$$x_{noisy} = x + N(0, p \cdot var(x))$$

where x is the original (unprocessed) Hastings–Powell series, and $N(0, p \cdot var(x))$ is a normally distributed random variable with mean 0 and variance $p \cdot var(x)$. The noisy time series were log-transformed, following the same preprocessing as the observational data, before smoothing.

1.3 Smoothing and model fitting

To remove the added noise, we smoothed the noisy time series using loess regression with span = 0.03, identical to the method applied to observational data (**see Methods section 2.5**). For each target variable (x, y, z), we tested all combinations of noise addition level (25th, 50th, 75th percentile) and noise reduction option (smoothed or unsmoothed). This resulted in

18 test conditions, plus three baseline conditions with no added noise and no smoothing, for a total of 21 conditions per target variable.

1.4 Response variable and S-map modeling

We calculated the growth rates of each target variable from the raw simulated data (no added noise and no smoothing) as log differences of abundance, consistent with the manuscript analysis (**see Methods section 3.2**). For each target variable, we fitted regularized S-map models and predicted the growth rate as a function of the densities of all interacting species and the target variable's own density dependence. The predictor time series corresponded to one of the 18 experimental conditions (i.e., a given combination of noise level and noise-reduction treatment).

1.5 Model selection, performance, and coefficient evaluation

We performed model selection via sequential leave-future-out cross-validation, evaluating a range of θ, α, and λ values for the regularized S-map models(**see Methods section 3.4**). From the best-performing models, we extracted: (1) model performance metrics (RMSE and Pearson correlation coefficient), and (2) interaction coefficients corresponding to each predictor variable. The inferred coefficients were then compared to the true coefficients of the original, noise-free Hastings–Powell model to assess estimation accuracy (**Figs. S22 – S24**), while predictive performance was evaluated across noise and smoothing conditions (**Fig. S25**).

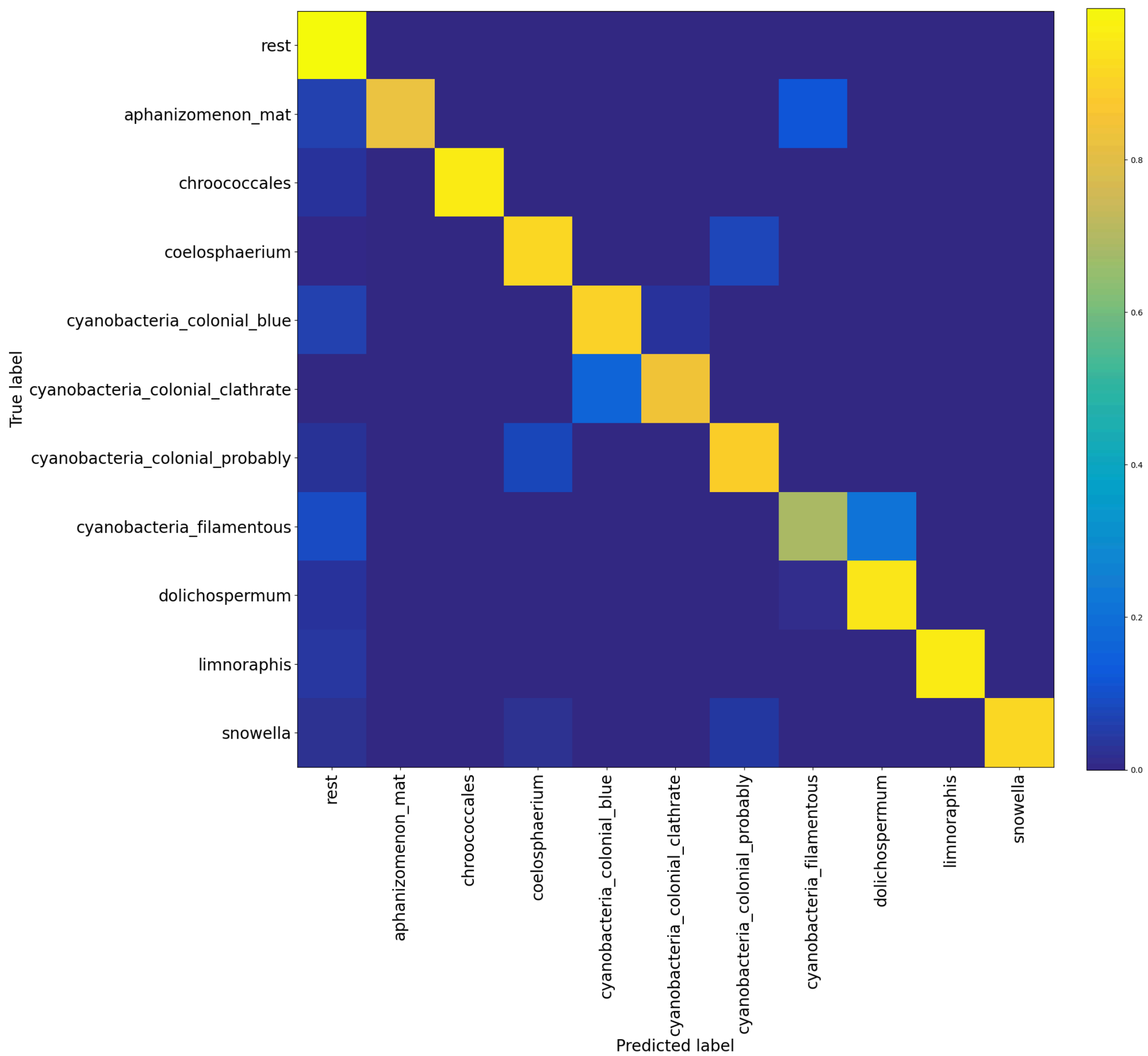

Fig. S1: Confusion matrix of the Cyanobacteria Classifier. Confusion matrix illustrating the performance of the cyanobacteria classifier designed to categorise 10 cyanobacteria classes from the rest in the 5.0x camera images. Training size: 27000 images; Validation size: 5785; Test size: 5787.

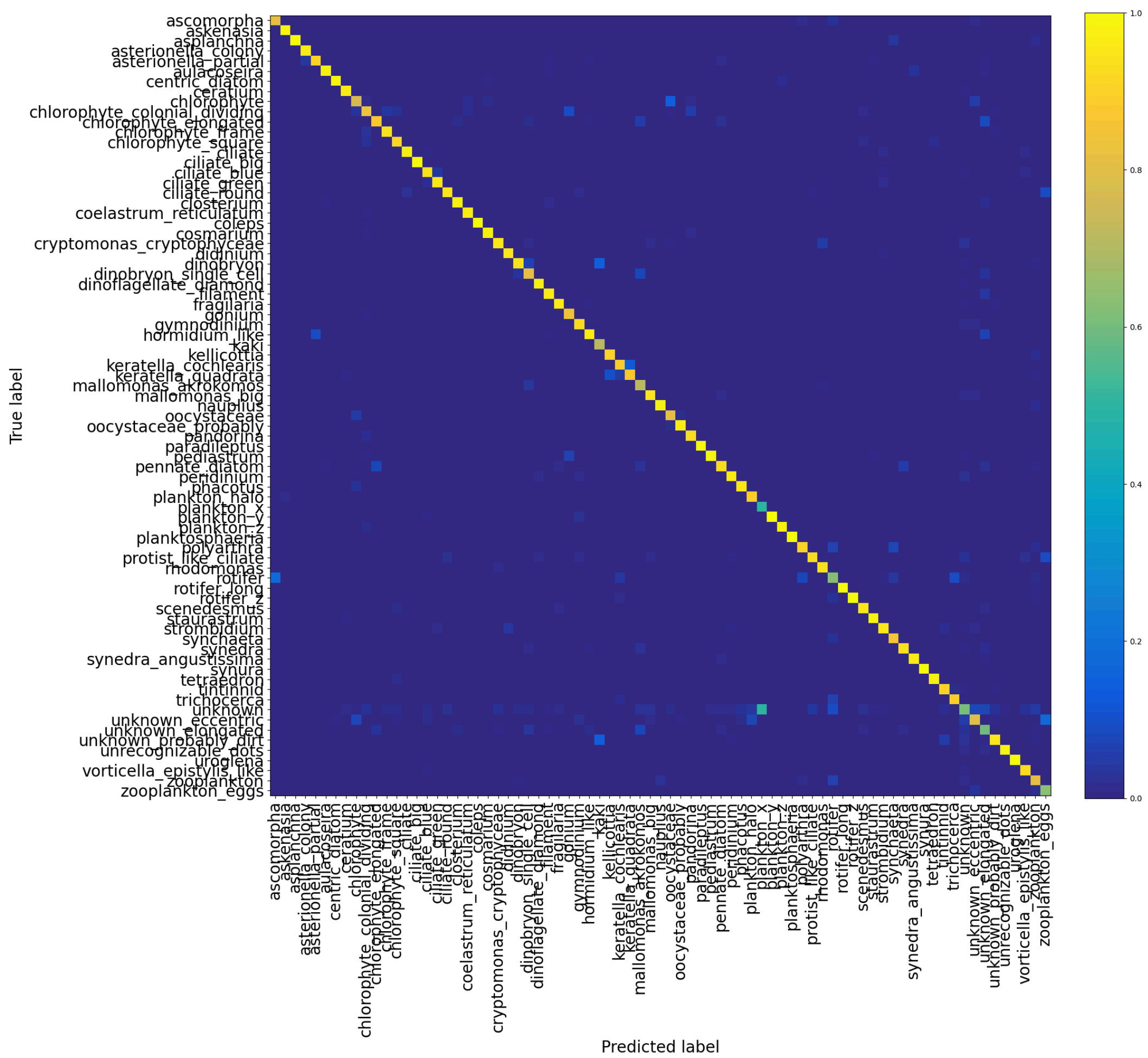

Fig. S2: Confusion matrix of the Phytoplankton Classifier. Confusion matrix illustrating the performance of the phytoplankton classifier designed to categorise the remaining classes in the 5.0x camera images following the implementation of the first Classifier. Training size: 24298 images; Validation size: 5206; Test size: 5208.

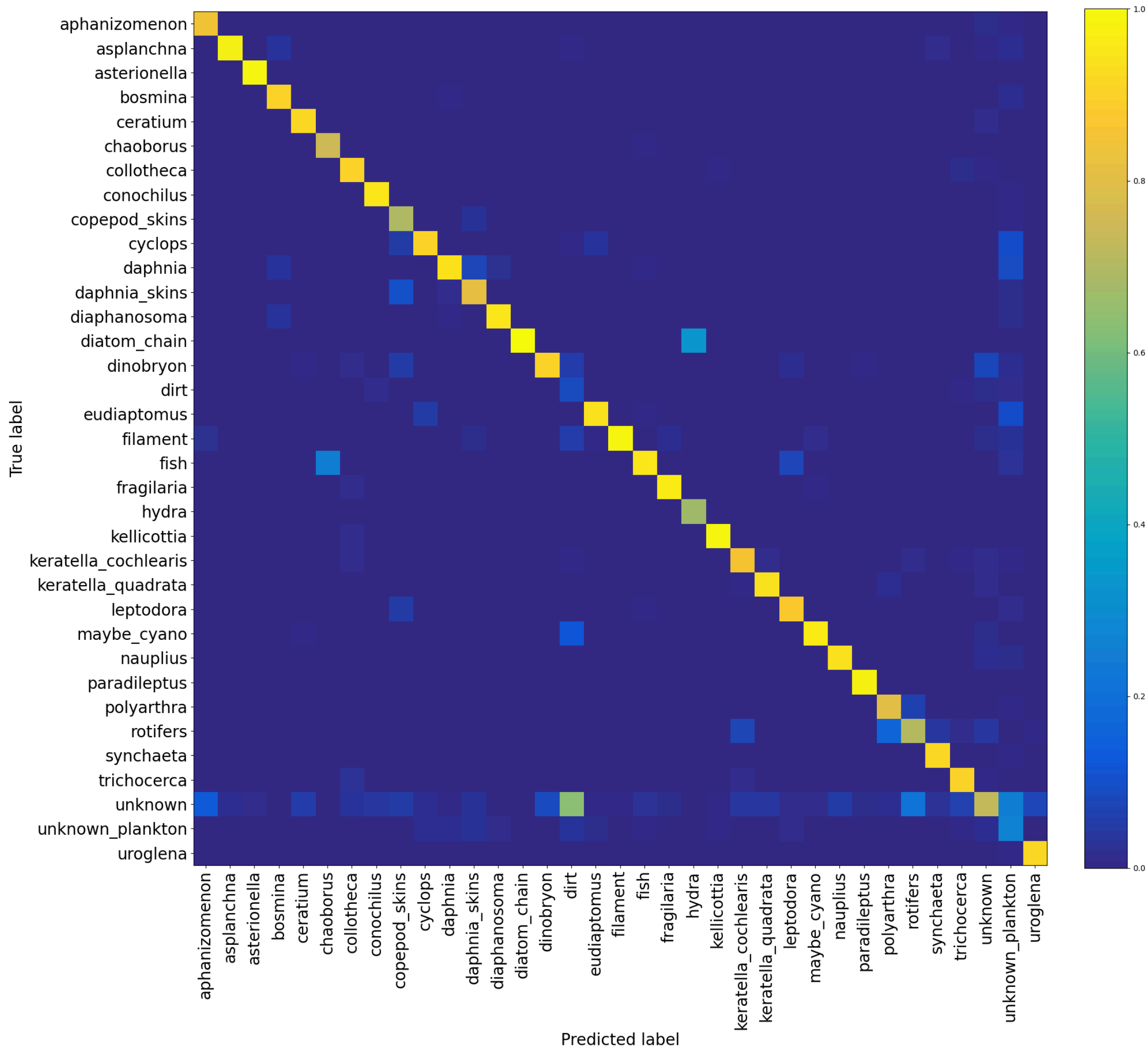

Fig. S3: Confusion matrix of the Zooplankton Classifier Confusion matrix illustrating the performance of the zooplankton classifier designed to categorise images of the 0.5x camera. Train size: 22680 images; Validation size: 4860; Test size: 4861.

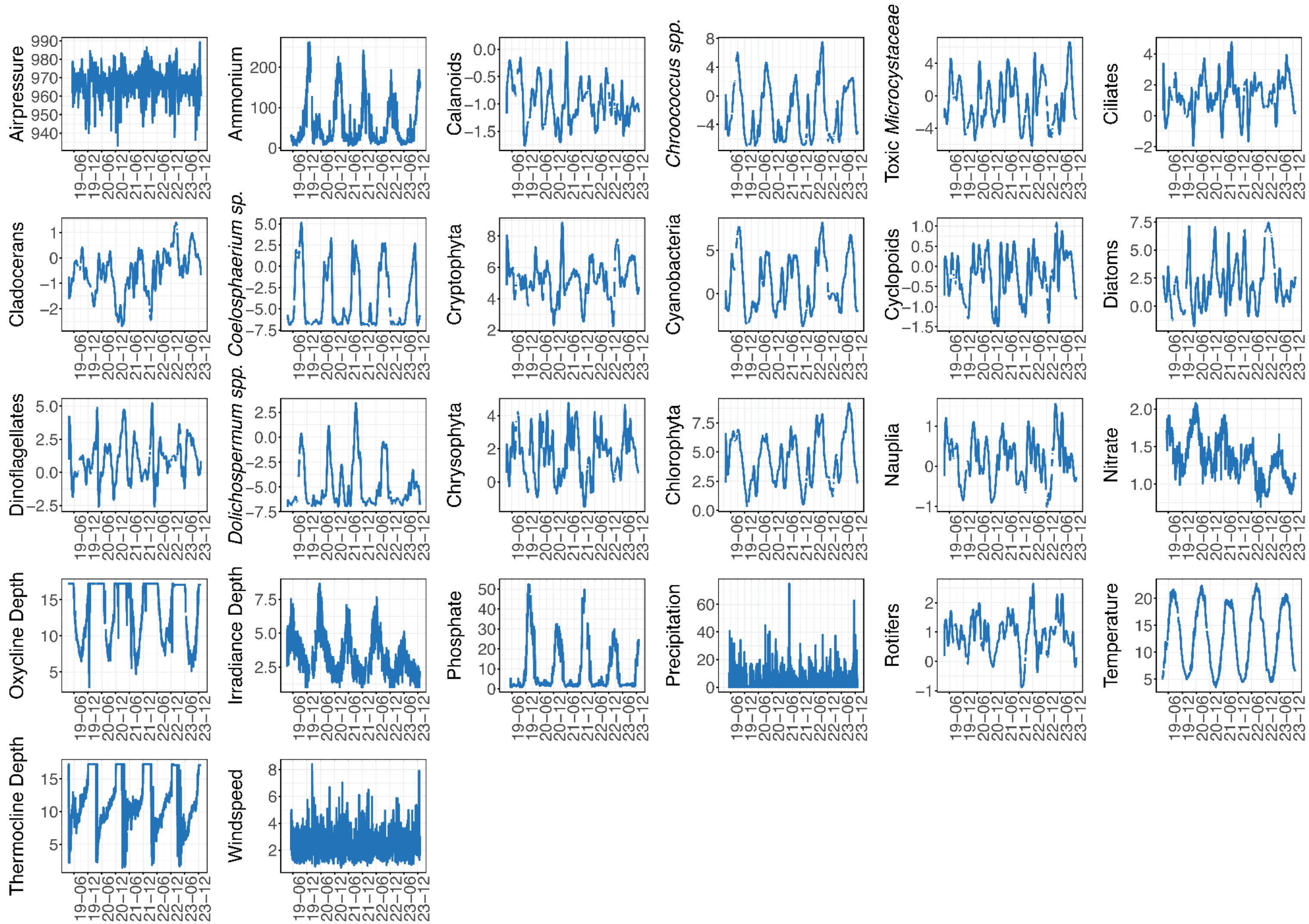

Fig. S4: Time series of biotic and abiotic predictors used in our study. Time series are smoothed with a loess function (span=0.03). Units as in **Fig. S15**.

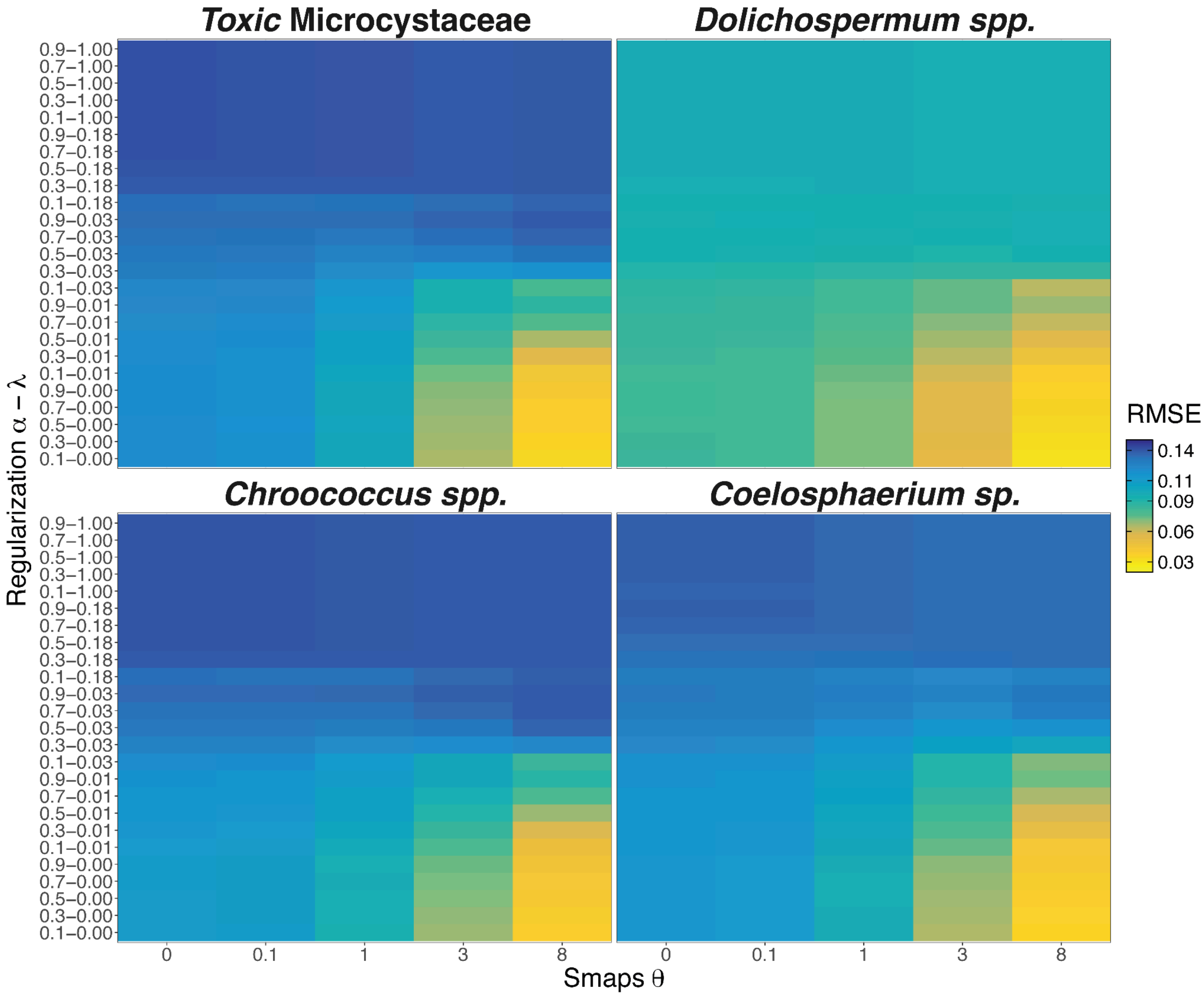

Fig. S5: Performance of MDR S-maps for all parameter combinations tested in the models (θ, α, λ) for all target cyanobacteria net growth rates. The results indicate that MDR S-map models with θ = 8 achieve the highest predictive skill, measured as root mean squared error (RMSE). Colours indicate the predictive skill (RMSE) obtained from leave-future-out cross-validation, with warmer colours (e.g., yellow) representing higher predictive accuracy and cooler colours (e.g., blue) indicating lower accuracy. See **materials and methods** for a detailed explanation of the model parameters and the implementation of the cross-validation analysis.

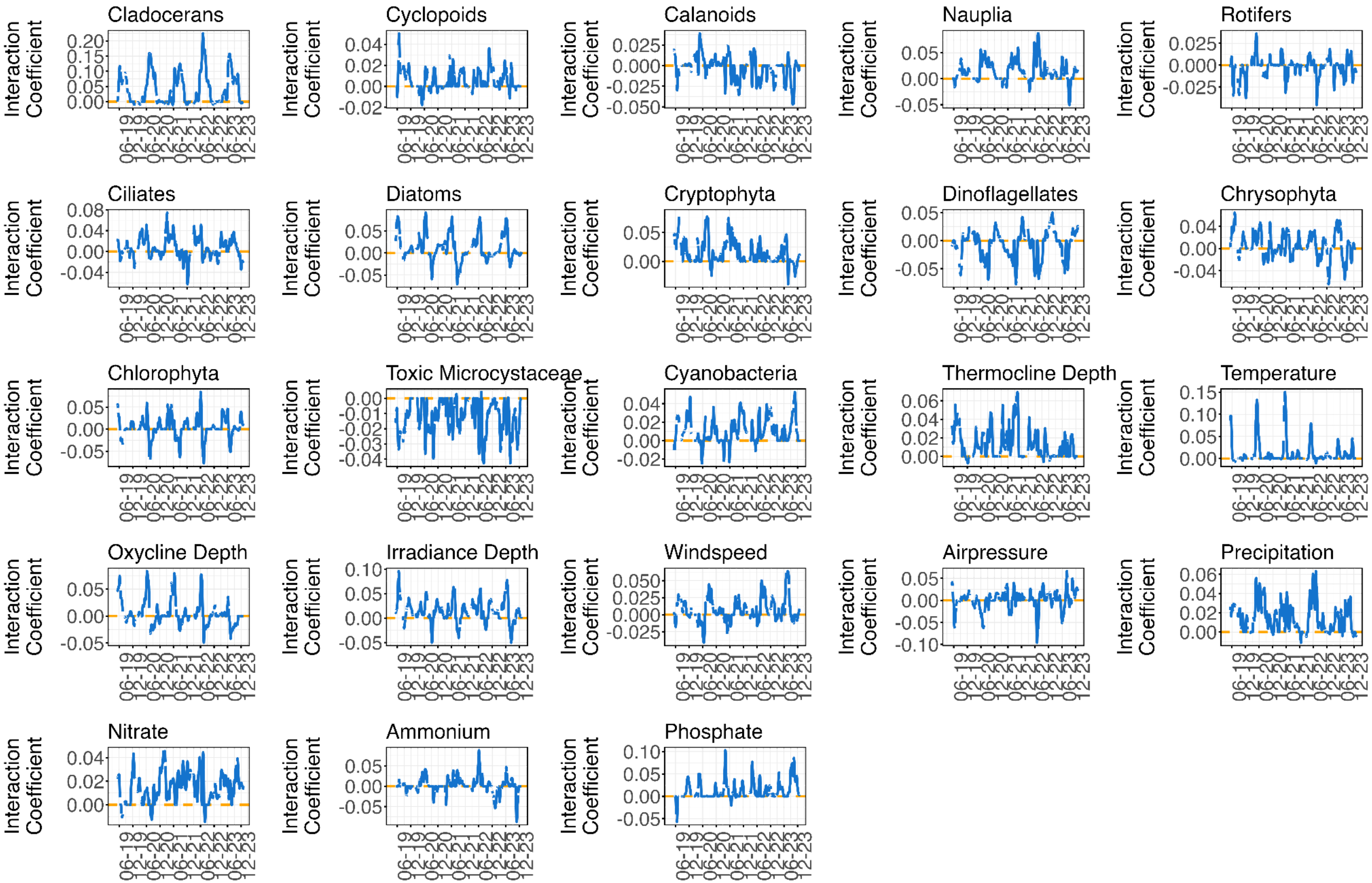

Fig. S6: Time series of coefficients from the regularised multiview-distance S-maps (best model) for Toxic *Microcystaceae*.

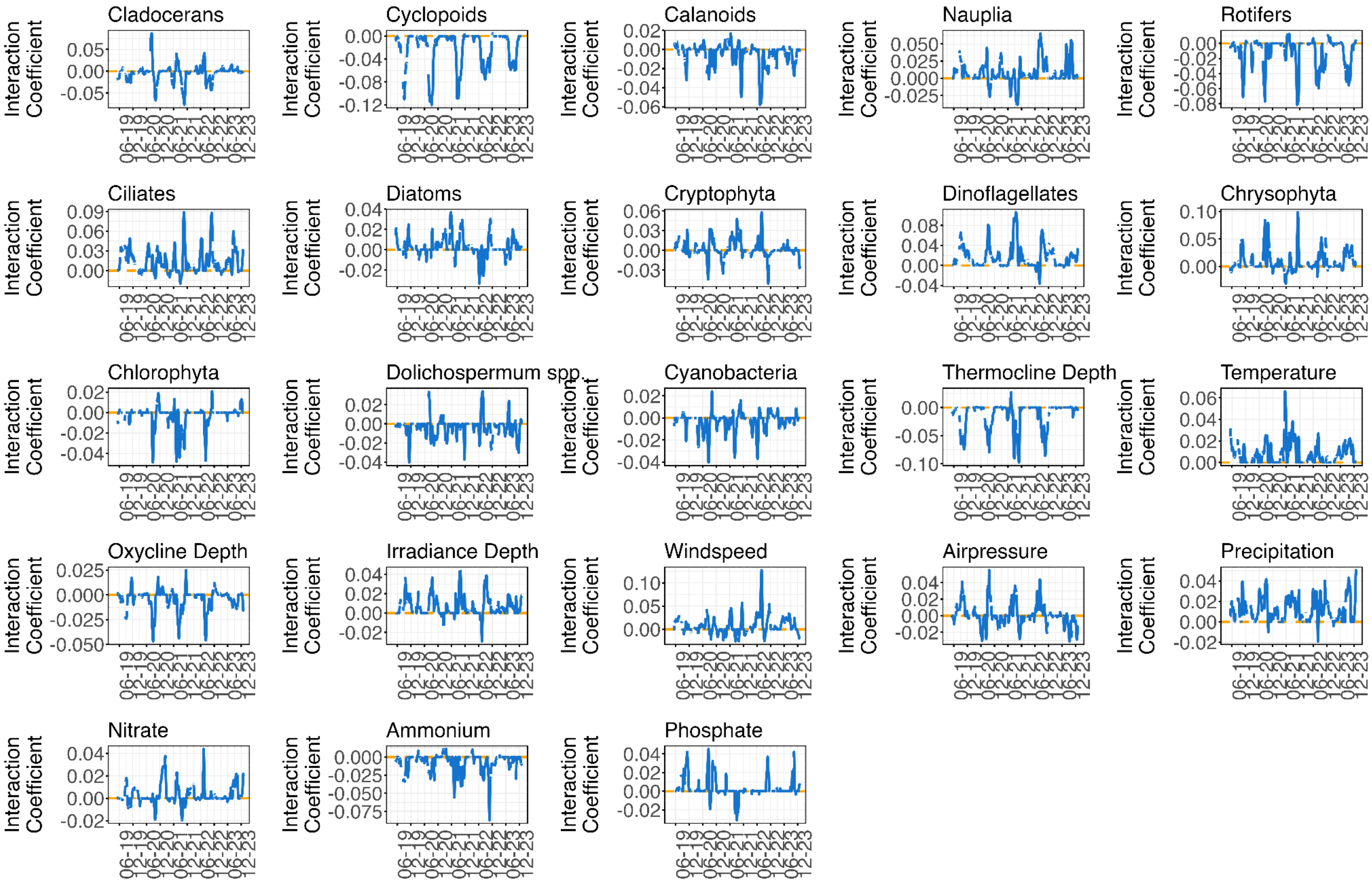

Fig. S7: Time series of coefficients from the regularised multiview-distance S-maps (best model) for *Dolichospermum spp*.

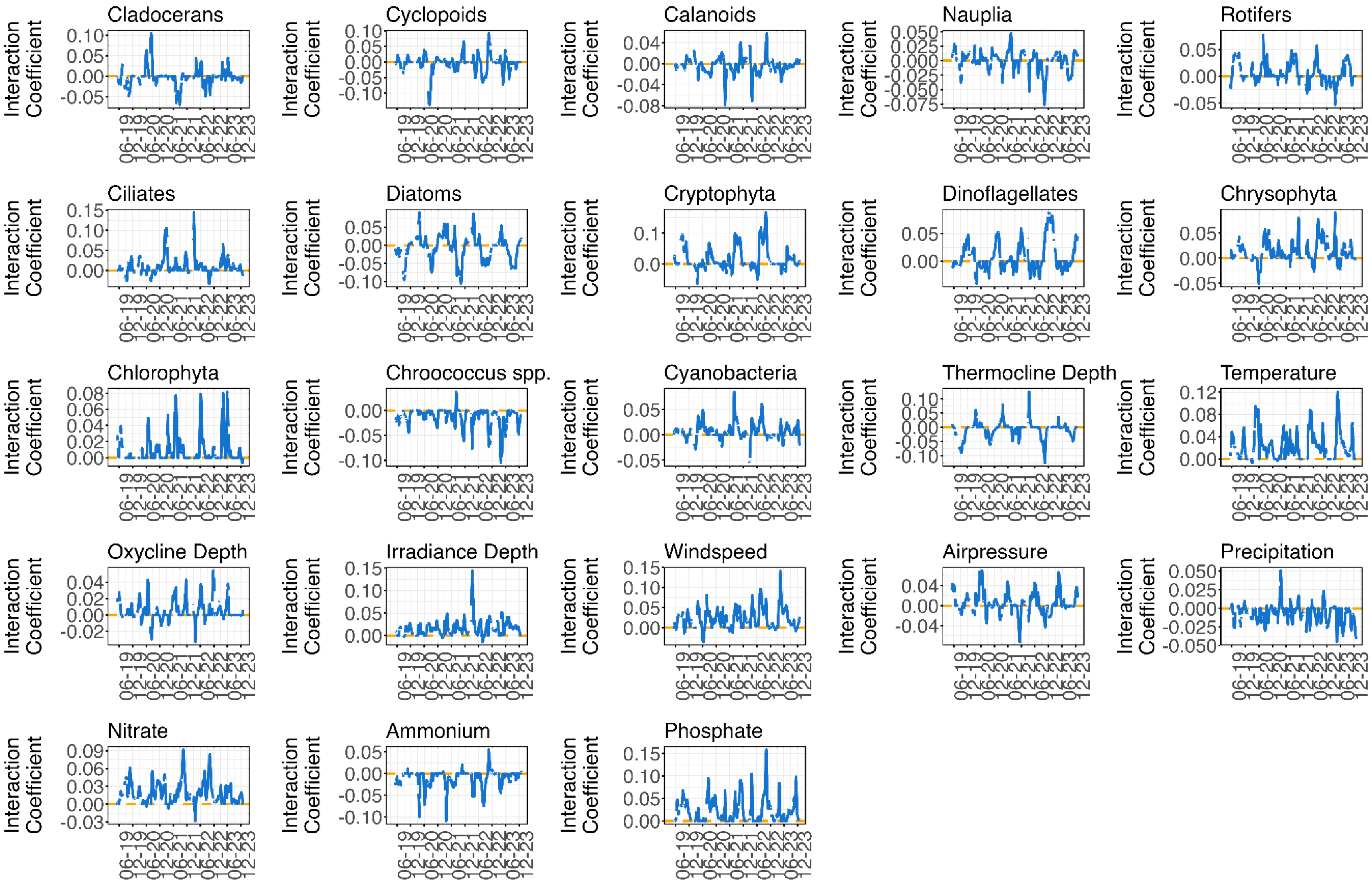

Fig. S8: Time series of coefficients from the regularised multiview-distance S-maps (best model) for *Chroococcus spp*.

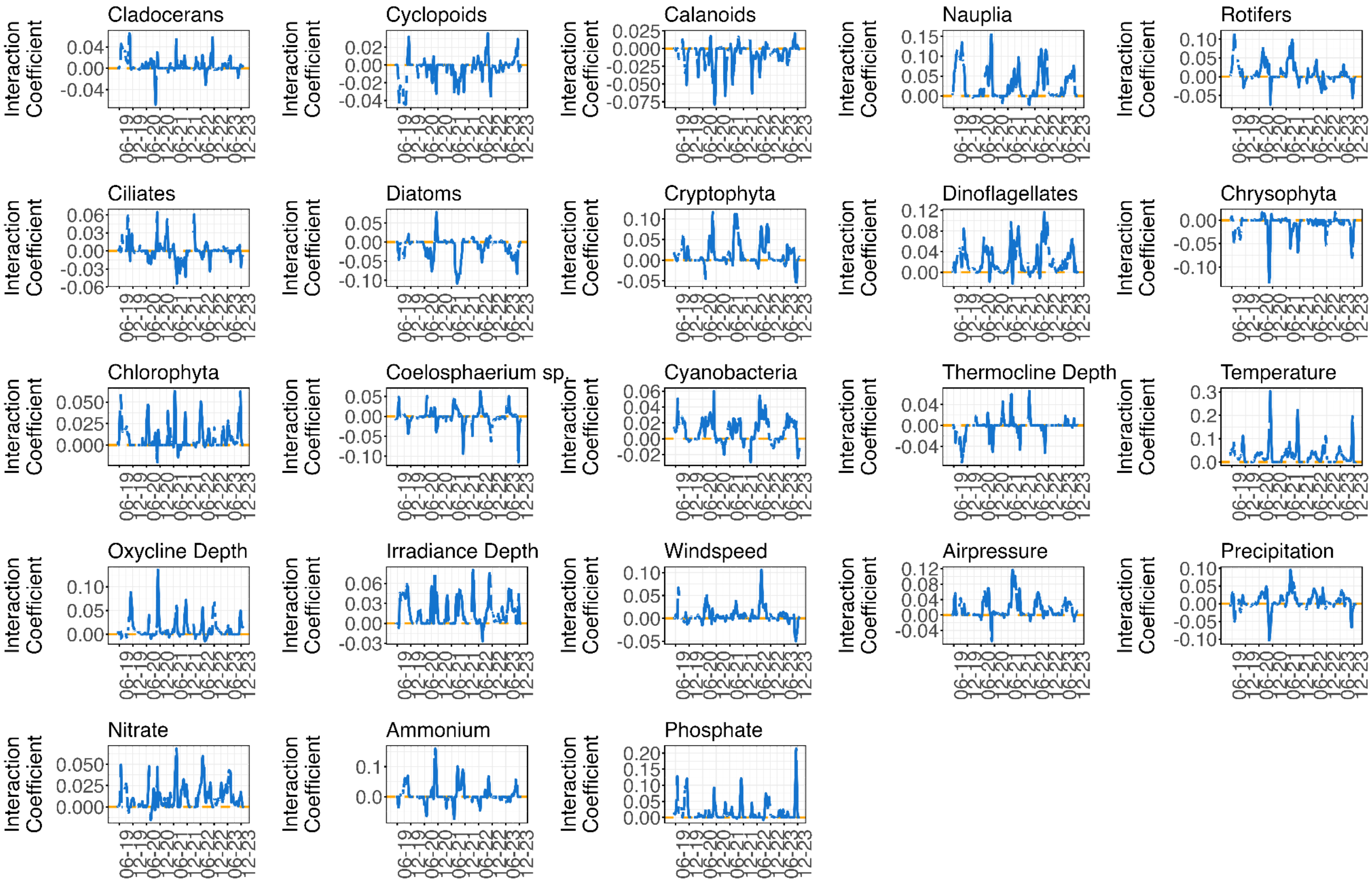

Fig. S9: Time series of coefficients from the regularised multiview-distance S-maps (best model) for *Coelosphaerium sp.*

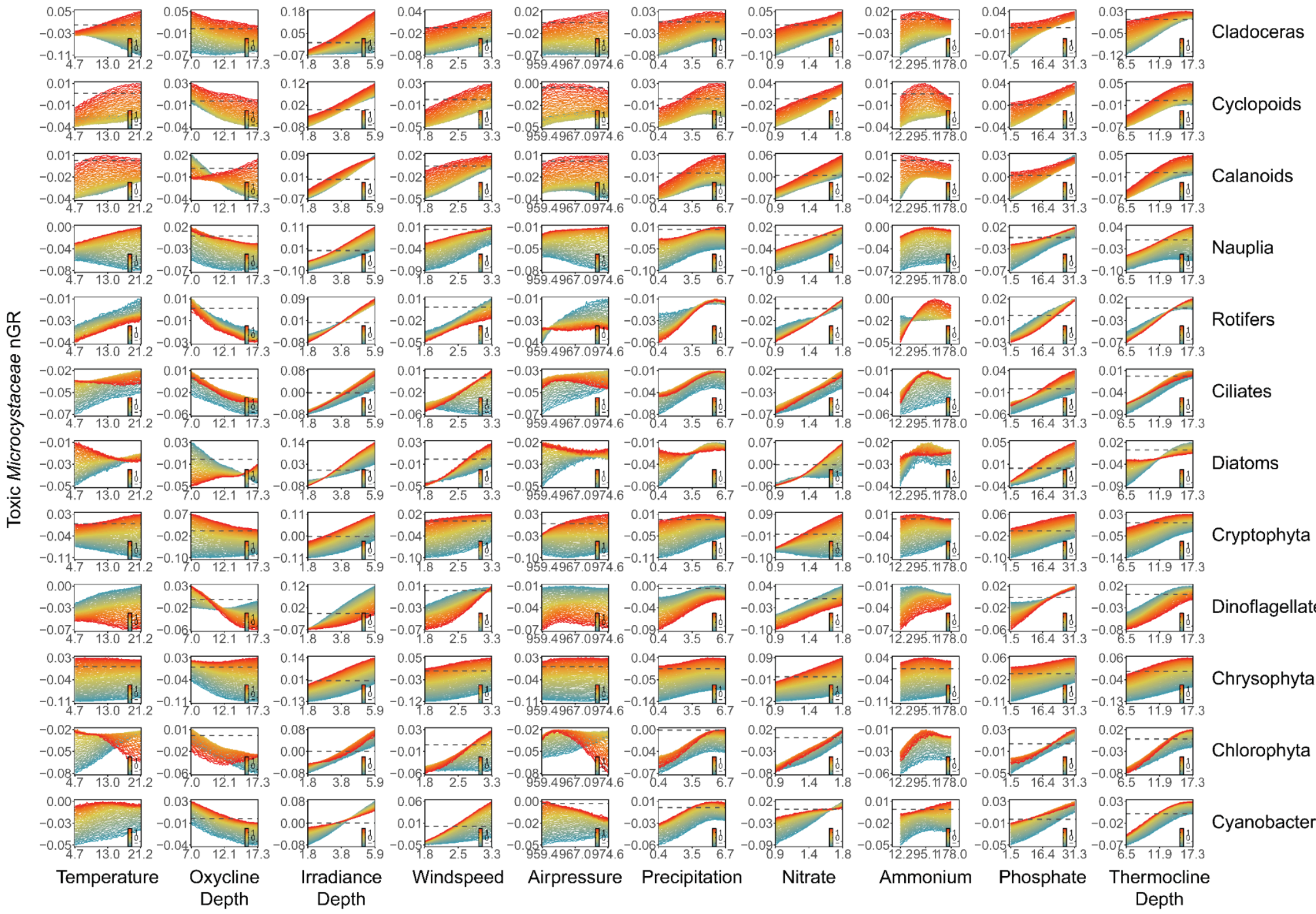

Fig. S10: Pairwise interactions between biotic and abiotic factors shape Toxic *Microcystaceae* net growth responses. This figure shows how Toxic *Microcystaceae* net growth rate (nGR, Ln(ind/mL) · day^{-1}) respond to combinations of environmental conditions, derived from regularized MDR S-maps analysis. Each panel shows predicted nGR when two variables (one biotic, one abiotic) vary across their observed range (5-95 percentile), while all other variables are held at median values. Lines show mean estimated responses from n=50 subsamples (see Materials and Methods) and colour indicates the scaled density of the biotic factor in Ln(ind/mL). **Abiotic effects**: Positive correlations with most variables, except for a negative relationship with oxycline depth. **Biotic effects**: Mostly positive interactions, with notable negative effects from rotifers and dinoflagellates. **Interactive effects**: Biotic factors can modify or reverse growth responses to abiotic conditions, specifically: Temperature responses are modified by cladocerans, cyclopoid copepods, copepods' nauplii, ciliates, diatoms, cryptophytes, dinoflagellates and chlorophytes; oxycline depth responses are altered by calanoid copepods, diatoms, cryptophytes, dinoflagellates, chrysophytes and chlorophytes; air pressure responses are influenced by copepods' nauplii, rotifers, ciliates, diatoms, chlorophytes and cyanobacteria; ammonium responses are modified by all biotic groups except chrysophytes and chlorophytes. **Units**: Biotic variables in Ln(ind/mL) and scaled; abiotic variables: temperature (°C), precipitation (mm/day), wind speed (m/s), phosphate (PO_4^{3-}, µg/L), ammonium (NH_4^+, µg/L), nitrate (NO_3^-, mg/L), air pressure (hPa), depth measurements (metres).

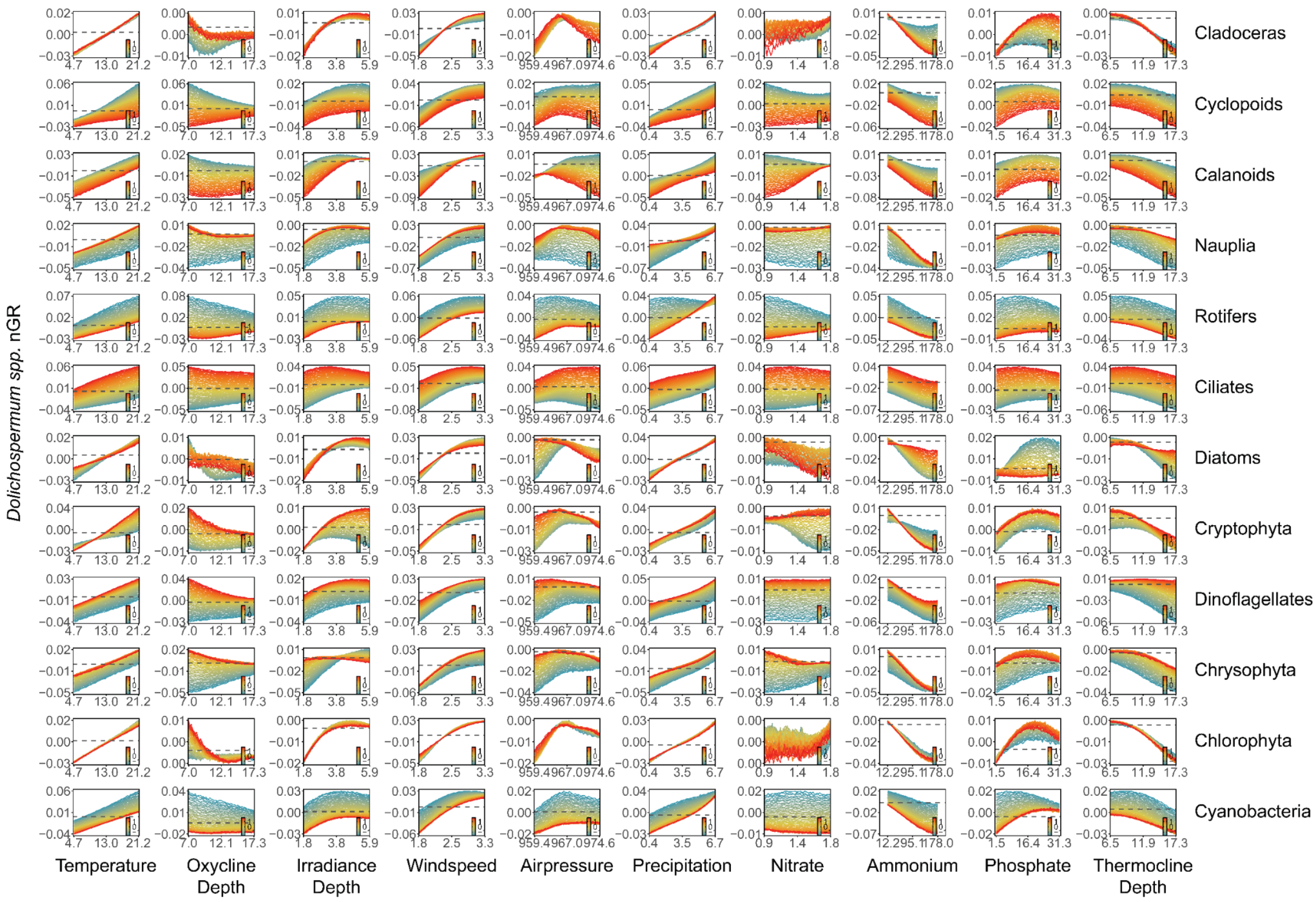

Fig. S11: Pairwise interactions between biotic and abiotic factors shape *Dolichospermum spp.* net growth responses. This figure shows how *Dolichospermum* net growth rate (nGR, Ln(ind/mL)·day^{-1}) responds to combinations of environmental conditions, derived from regularised MDR S-maps analysis (as in **Fig. S10**). Each panel shows predicted nGR when two variables (one biotic, one abiotic) vary across their observed range (5-95 percentile), while all other variables are held at median values. Lines show mean estimated responses from n=50 subsamples (see Materials and Methods) and colour indicates the scaled density of the biotic factor in Ln(ind/mL). **Abiotic effects**: Positive correlations with temperature, light irradiance, wind speed, precipitation, and phosphate; negative relationships with oxycline depth, ammonium, and thermocline depth; unimodal response to air pressure; negligible effects of nitrate. **Biotic effects**: Negative effects from cyclopoid and calanoid copepods, rotifers, and cyanobacteria; positive effects from nauplii, ciliates, dinoflagellates, and chrysophytes. **Interactive effects**: Biotic factors can modify or reverse growth responses to abiotic conditions, specifically: Oxycline depth responses are modified by cyclopoid copepods, rotifers, diatoms, dinoflagellates, chrysophytes, and chlorophytes; nitrate responses are altered by cladocerans, calanoid copepods, rotifers, diatoms, cryptophytes, and chrysophytes; phosphate responses are influenced by rotifers and diatoms. **Units** as in **Fig. S10**.

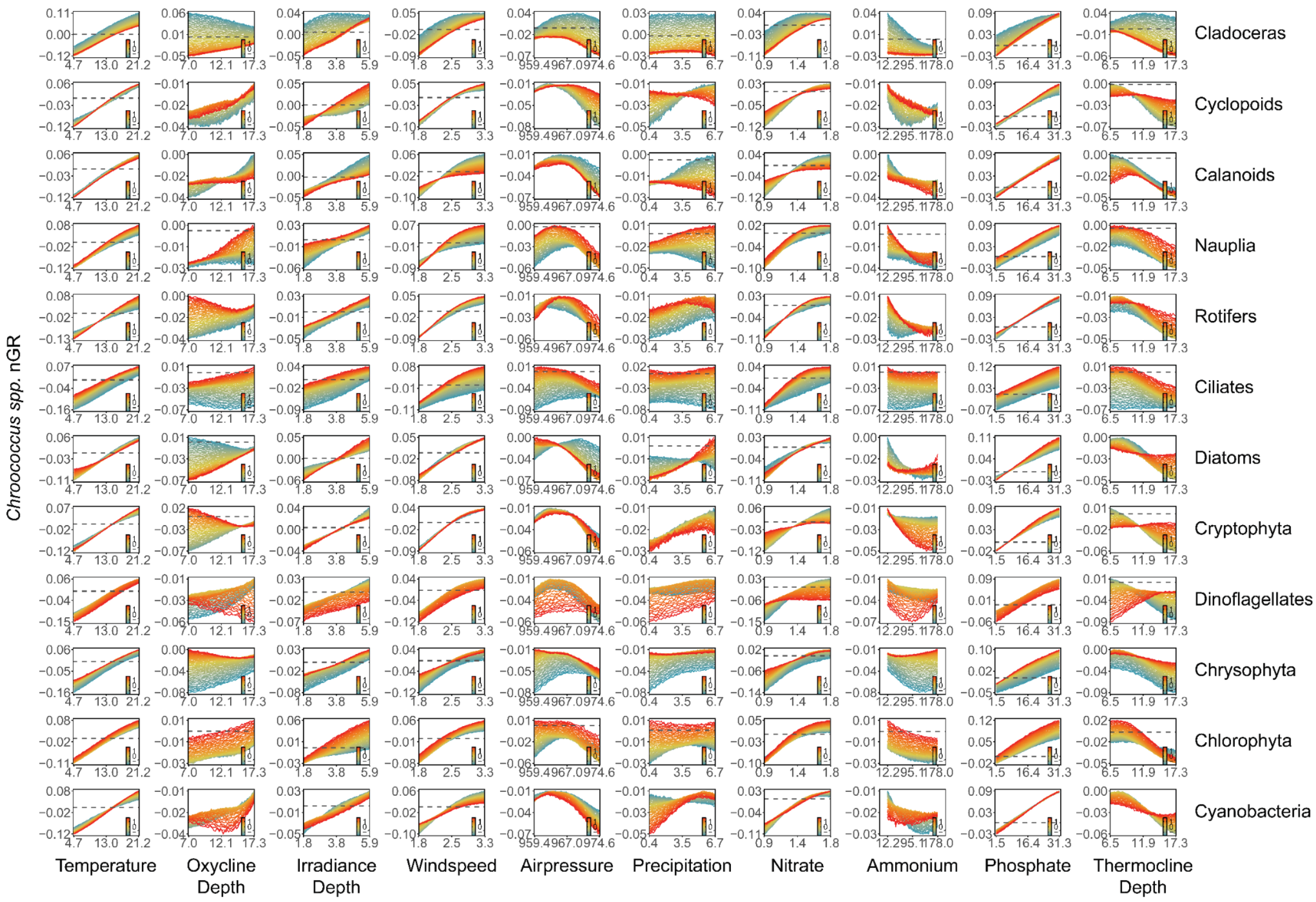

Fig. S12: Pairwise interactions between biotic and abiotic factors shape *Chroococcus spp.* net growth responses. This figure shows how *Chroococcus* net growth rate(nGR, Ln(ind/mL)·day^{-1}) responds to combinations of environmental conditions, derived from regularized MDR S-maps analysis (as in **Fig. S10**). Each panel shows predicted nGR when two variables (one biotic, one abiotic) vary across their observed range (5-95 percentile), while all other variables are held at median values. Lines show mean estimated responses from n=50 subsamples (see Materials and Methods) and colour indicates the scaled density of the biotic factor in Ln(ind/mL). **Abiotic effects**: Positive correlations with temperature, oxycline depth, light irradiance, wind speed, nitrate, and phosphate; negative relationships with ammonium and thermocline depth; unimodal response to air pressure; negligible effects of precipitation. **Biotic effects**: Negative effects from cladocerans, cyclopoid copepods, and diatoms; positive effects from calanoid copepods, their nauplii, rotifers, ciliates, chrysophytes, and chlorophytes. **Interactive effects**: Biotic factors can modify or reverse growth responses to abiotic conditions, specifically: Oxycline depth responses are modified by most biotic groups except cyclopoid copepods and chlorophytes; precipitation responses are altered by copepods (cyclopoid, calanoid, nauplii), diatoms, dinoflagellates, chrysophytes, chlorophytes, and cyanobacteria; phosphate responses are influenced by cladocerans, ciliates, diatoms, cryptophytes, and dinoflagellates. **Units** as in **Fig. S10**.

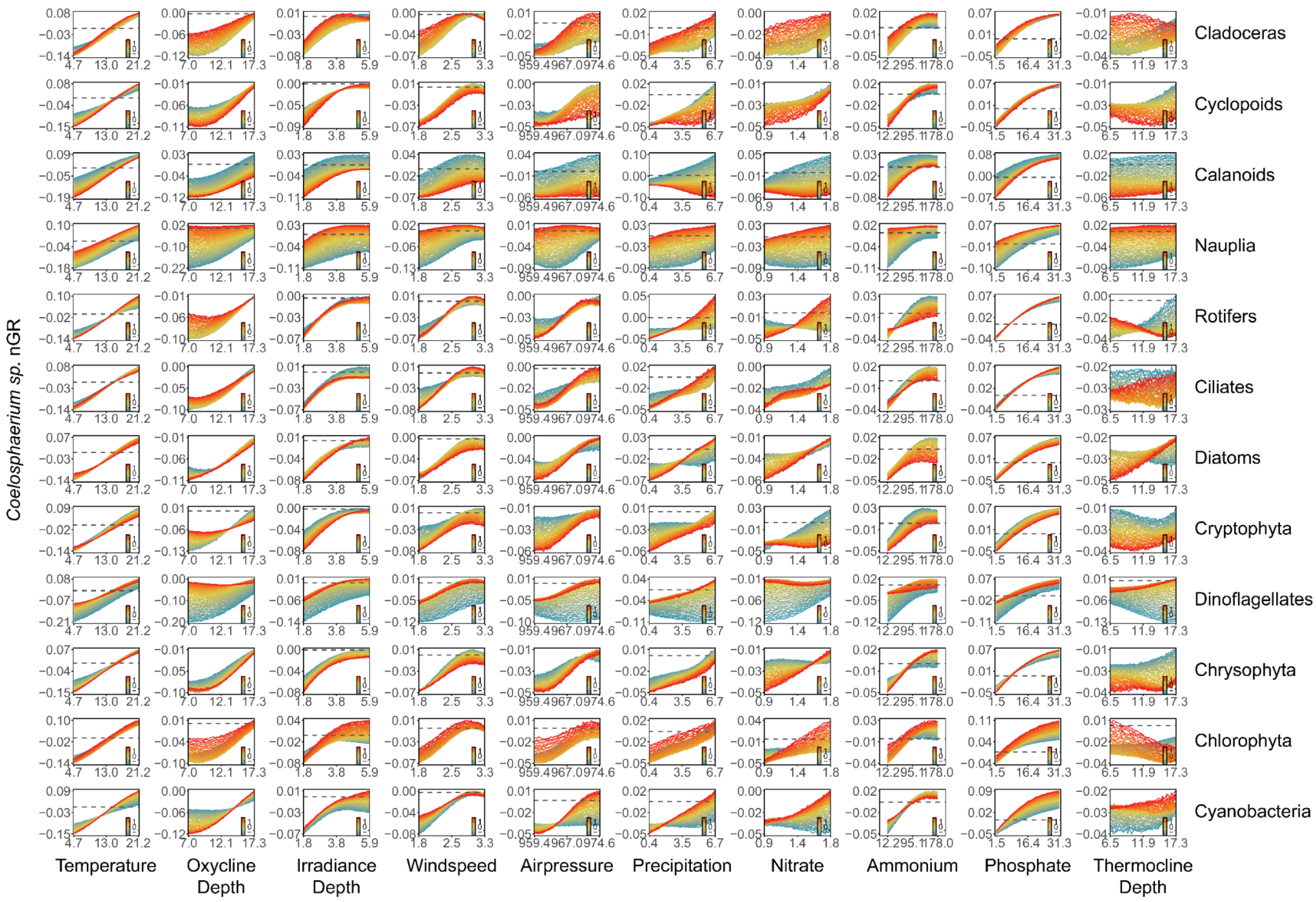

Fig. S13: Pairwise interactions between biotic and abiotic factors shape *Coelosphaerium sp.* net growth responses. This figure shows how *Coelosphaerium* net growth rate (nGR, Ln(ind/mL)·day^{-1}) responds to combinations of environmental conditions, derived from regularised MDR S-maps analysis (as in **Fig. S10**). Each panel shows predicted nGR when two variables (one biotic, one abiotic) vary across their observed range (5-95 percentile), while all other variables are held at median values. Lines show mean estimated responses from n=50 subsamples (see Materials and Methods) and colour indicates the scaled density of the biotic factor in Ln(ind/mL). **Abiotic effects**: Positive correlations with all abiotic variables. **Biotic effects**: Negative effects from cyclopoid and calanoid copepods, cryptophytes, and chrysophytes; positive effects from cladocerans, copepods, dinoflagellates, and cyanobacteria. **Interactive effects**: Biotic factors can modify or reverse growth responses to abiotic conditions, specifically: Air pressure, precipitation, and nitrate responses are modified by calanoid copepods and their nauplii, diatoms, cryptophytes, dinoflagellates, and cyanobacteria; thermocline depth responses are altered by cladocerans, cyclopoid copepods, rotifers, diatoms, dinoflagellates, and chlorophytes. **Units** as in **Fig. S10**.

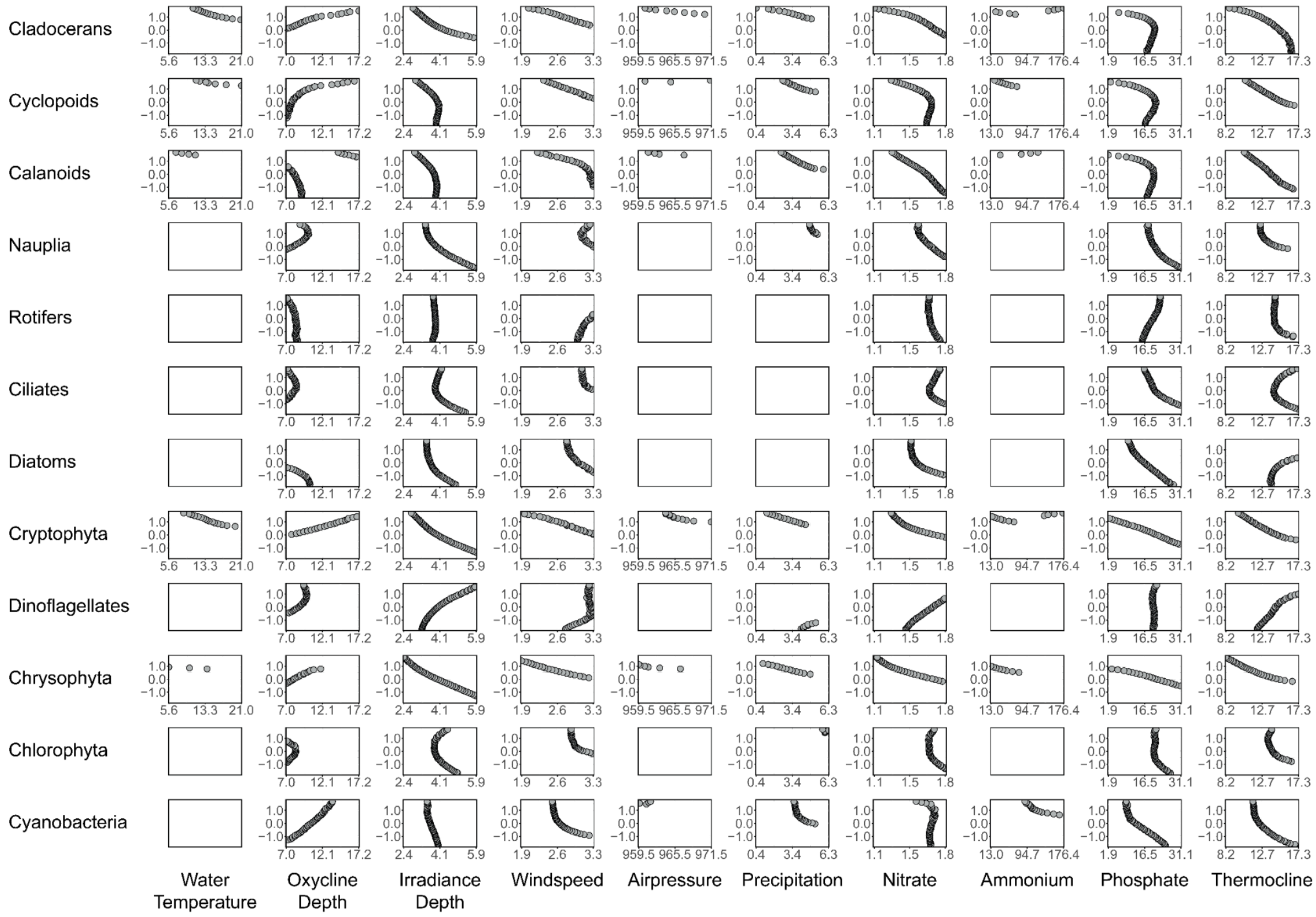

Fig. S14: Zero net growth analysis for Toxic *Microcystaceae* bloom formation across biotic-abiotic variable combinations. This figure analyzes bloom initiation conditions by identifying zero-net growth isoclines from the response functions in **Fig. S10**. Each panel shows how the minimum environmental conditions required for blooming change with increasing densities of different planktonic groups. **Response patterns**: Positive correlations between blooming points and organism density indicate bloom inhibition (higher environmental thresholds needed); negative correlations indicate bloom facilitation (lower thresholds needed). **Key influences**: Cladocerans, cryptophytes, and chrysophytes strongly affect blooming conditions across all tested abiotic ranges. **Notable absences**: White panels indicate conditions where blooming was never observed due to consistently negative or positive growth rates. **Units** as in **Fig. S10**.

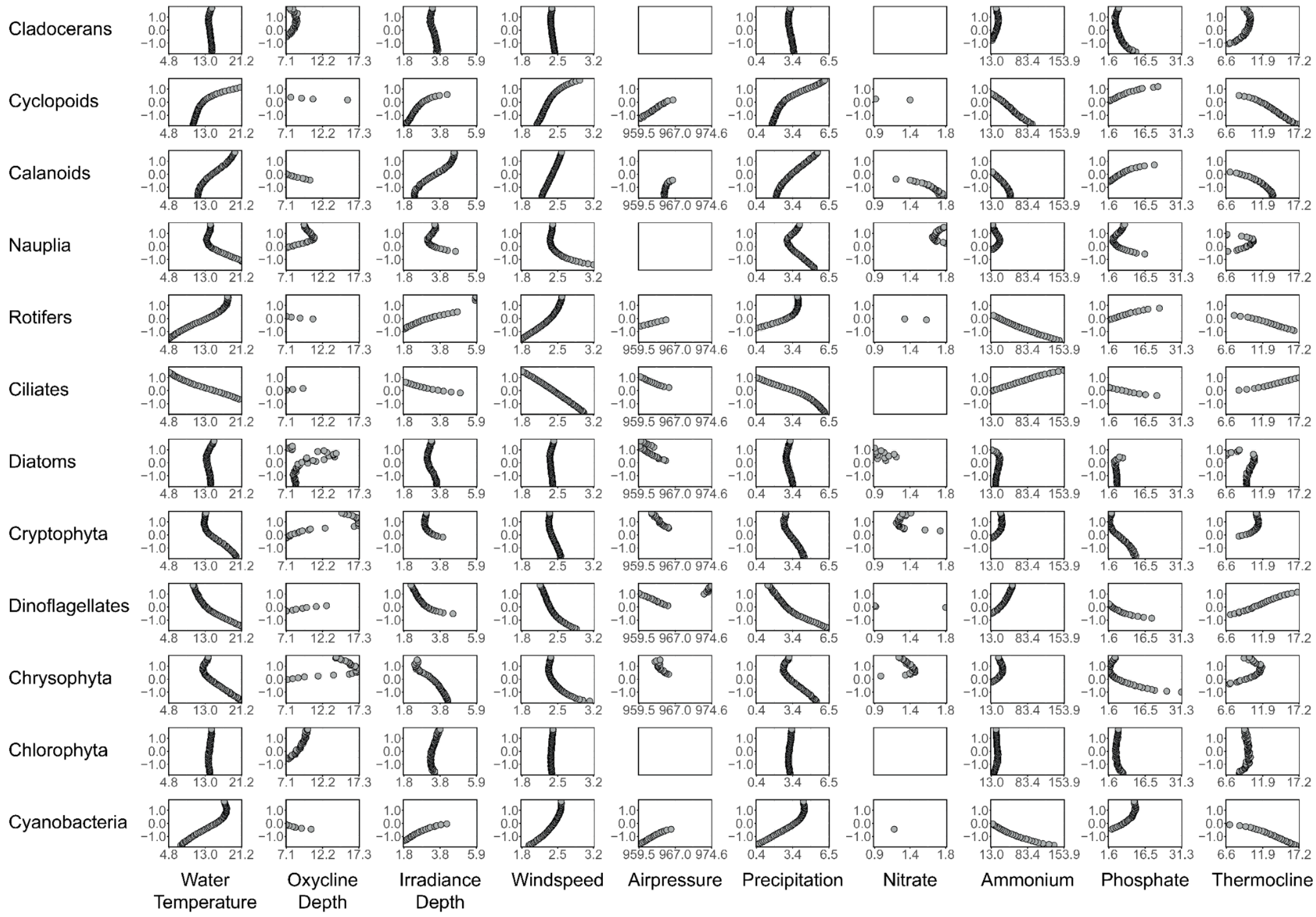

Fig. S15: Zero net growth analysis for *Dolichospermum spp.* bloom formation across biotic-abiotic variable combinations. This figure analyzes bloom initiation conditions by identifying zero-net growth isoclines from the response functions in **Fig. S11**. Each panel shows how the minimum environmental conditions required for blooming change with increasing densities of different planktonic groups. **Response patterns**: Positive correlations between blooming points and organism density indicate bloom inhibition (higher environmental thresholds needed); negative correlations indicate bloom facilitation (lower thresholds needed). **Key influences**: Rotifers, ciliates, dinoflagellates, and cyanobacteria strongly affect blooming conditions across most abiotic scenarios and throughout their observed density ranges. **Notable absences**: White panels indicate conditions where blooming was never observed due to consistently negative or positive growth rates. **Units** as in **Fig. S10**.

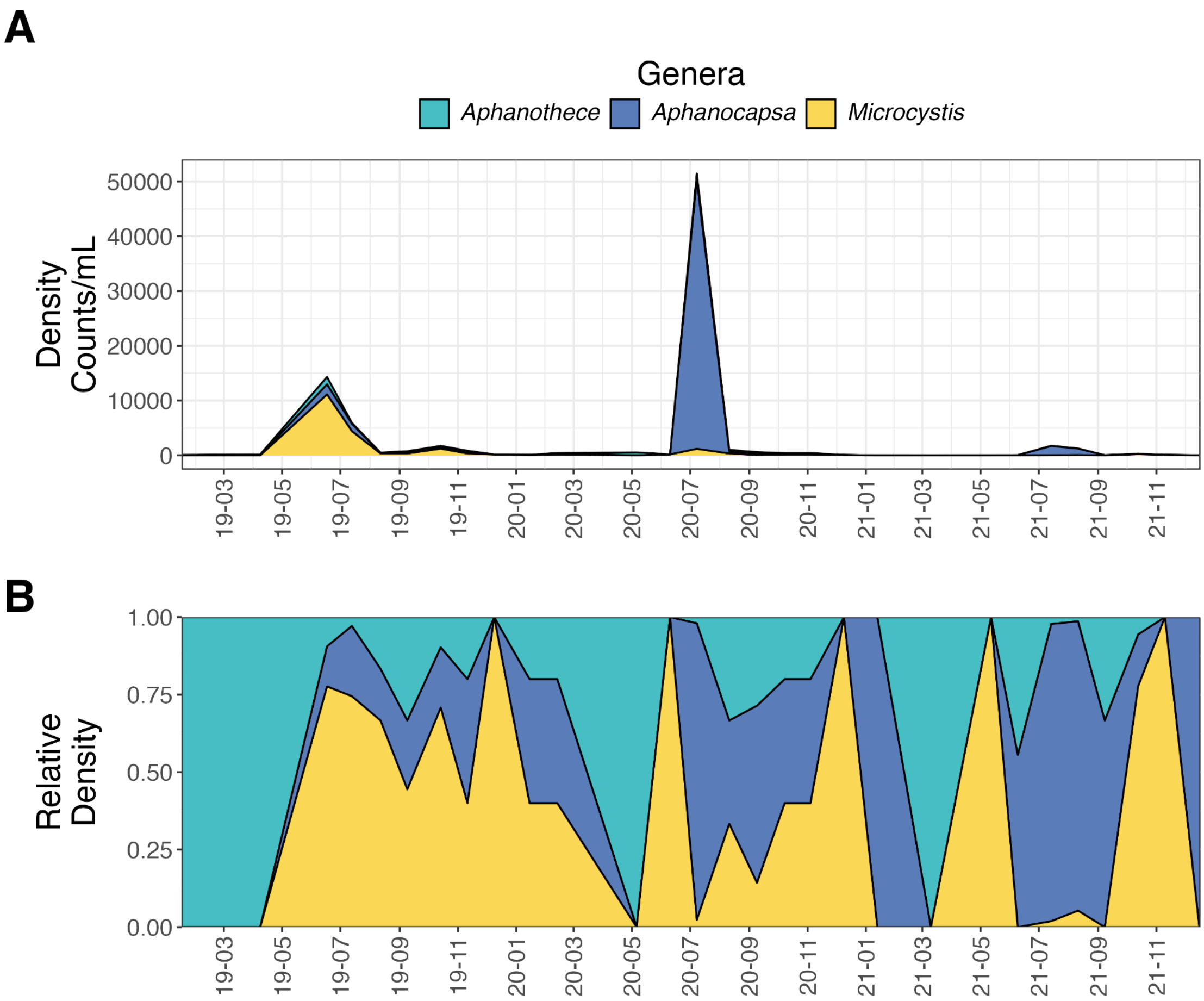

Fig. S16: Taxonomic composition of Toxic *Microcystaceae* based on monthly microscopy data from Lake Greifensee. (A) Absolute abundances (cells·mL^{-1}) of the three colony-forming genera *Microcystis*, *Aphanocapsa*, and *Aphanothece*, which together constitute the "Toxic *Microcystaceae*" group. **(B)** Relative contributions (%) of these three genera to the total group abundance over time. In our high-throughput plankton monitoring system, these taxa are aggregated by the neural network image classifier into the classes **"cyanobacteria_colonial_blue"** and **"cyanobacteria_colonial_clathrate"** (see **Table S2**), which collectively define the "Toxic *Microcystaceae*" group. This grouping reflects the highly similar colony-forming morphologies of these genera, which are challenging to distinguish reliably in automated image analysis**.**

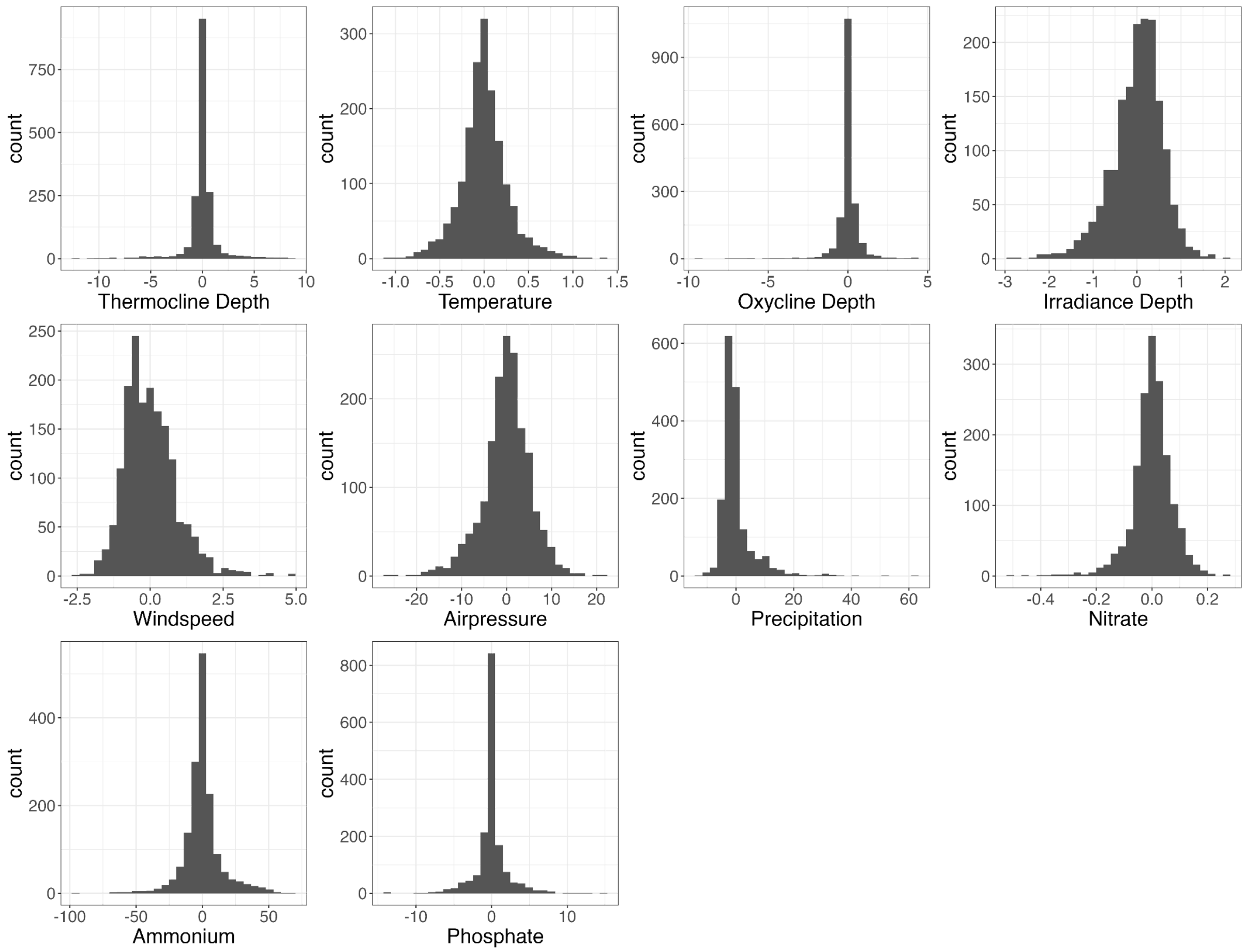

Fig. S17: Histograms of the residuals obtained after smoothing the abiotic variables using a loess function (span = 0.03) to reduce random noise in the raw data.

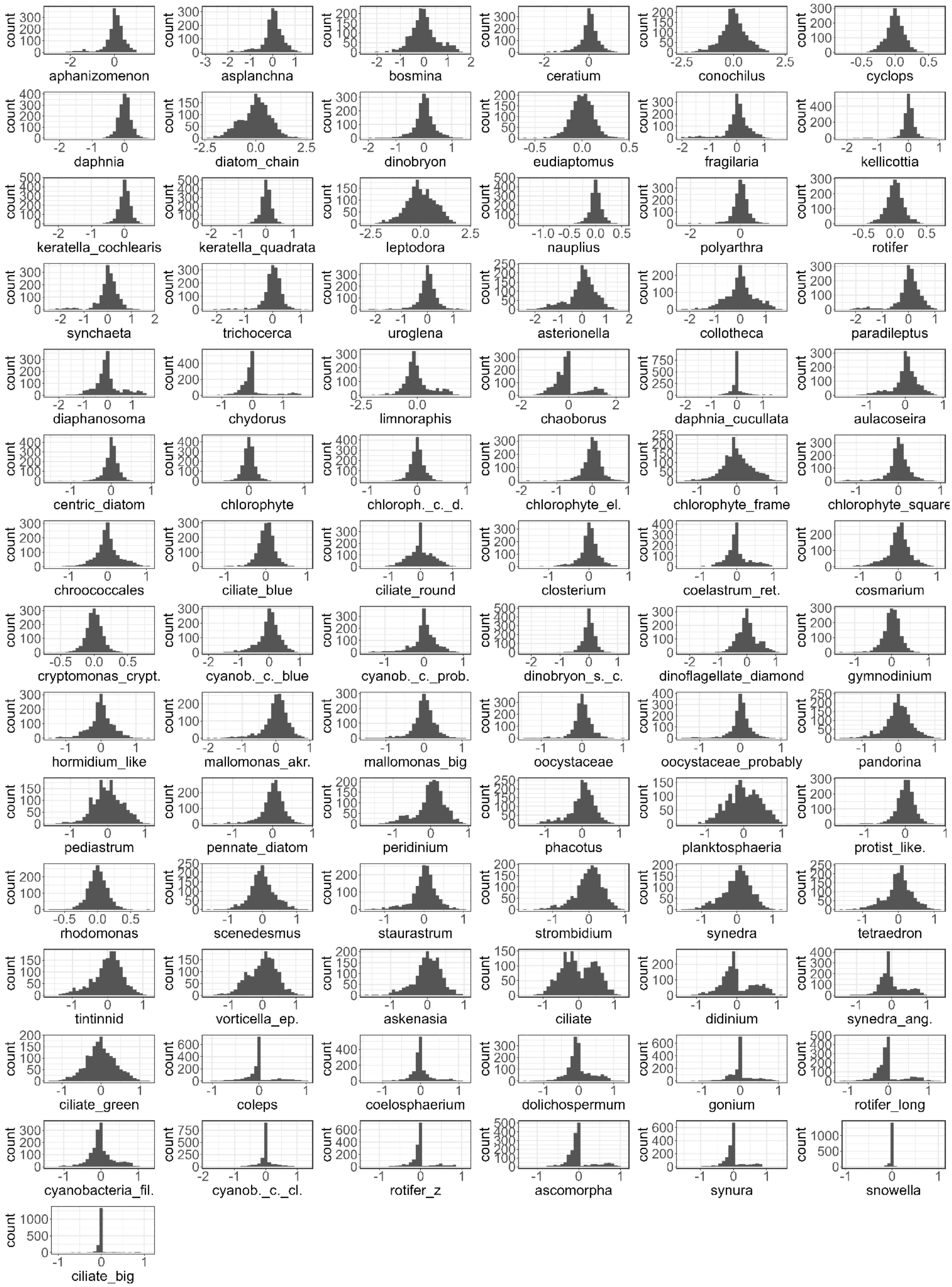

Fig. S18: Histograms of the residuals obtained after smoothing the biotic variables using a loess function (span = 0.03) to reduce random noise in the raw data. The biotic variables represent individual taxa before their aggregation into broader taxonomic groups (see **Tables S1 and S2** for the groupings).

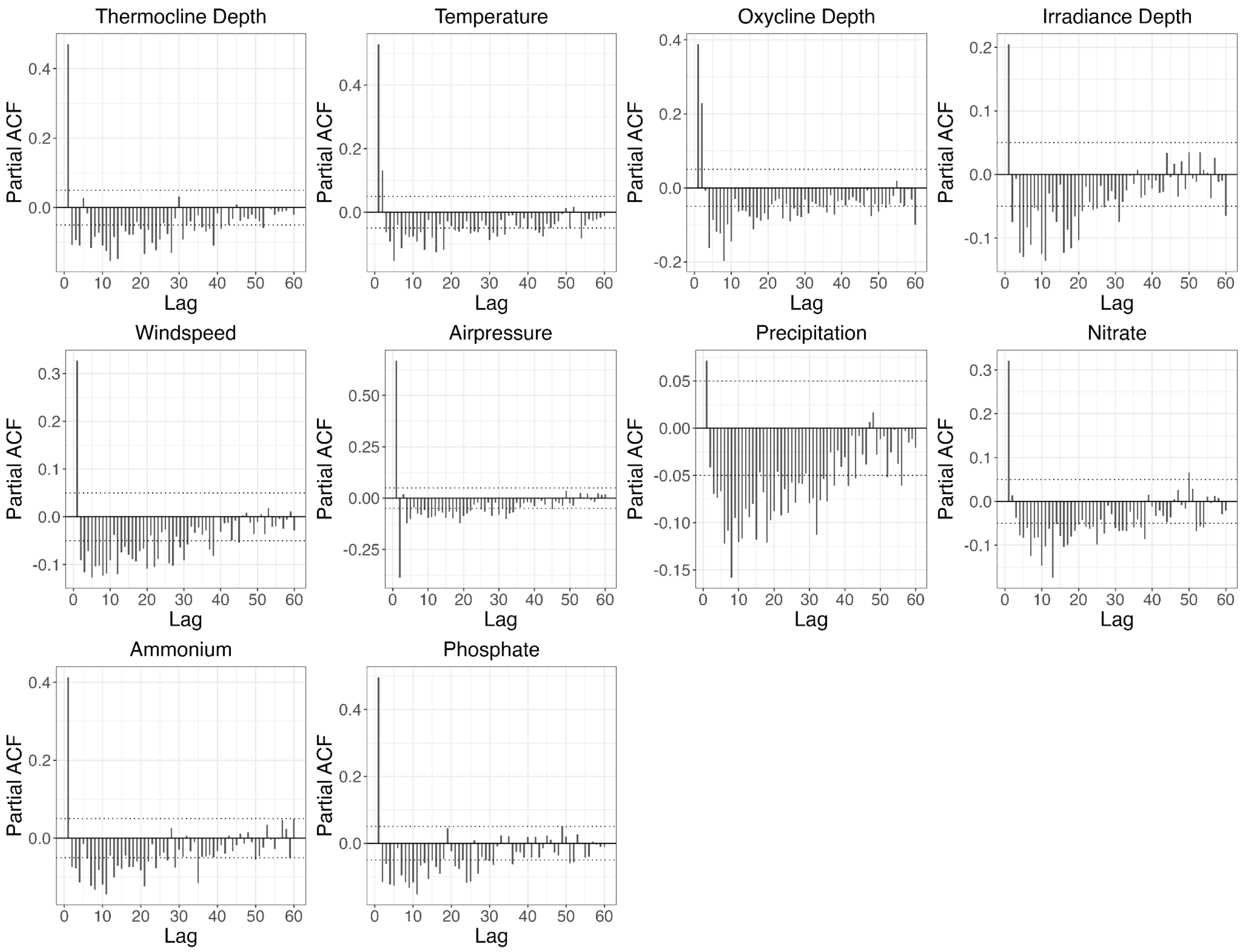

Fig. S19: Partial autocorrelation plots of the residuals obtained after smoothing the abiotic variables using a loess function (span = 0.03) to reduce random noise in the raw data.

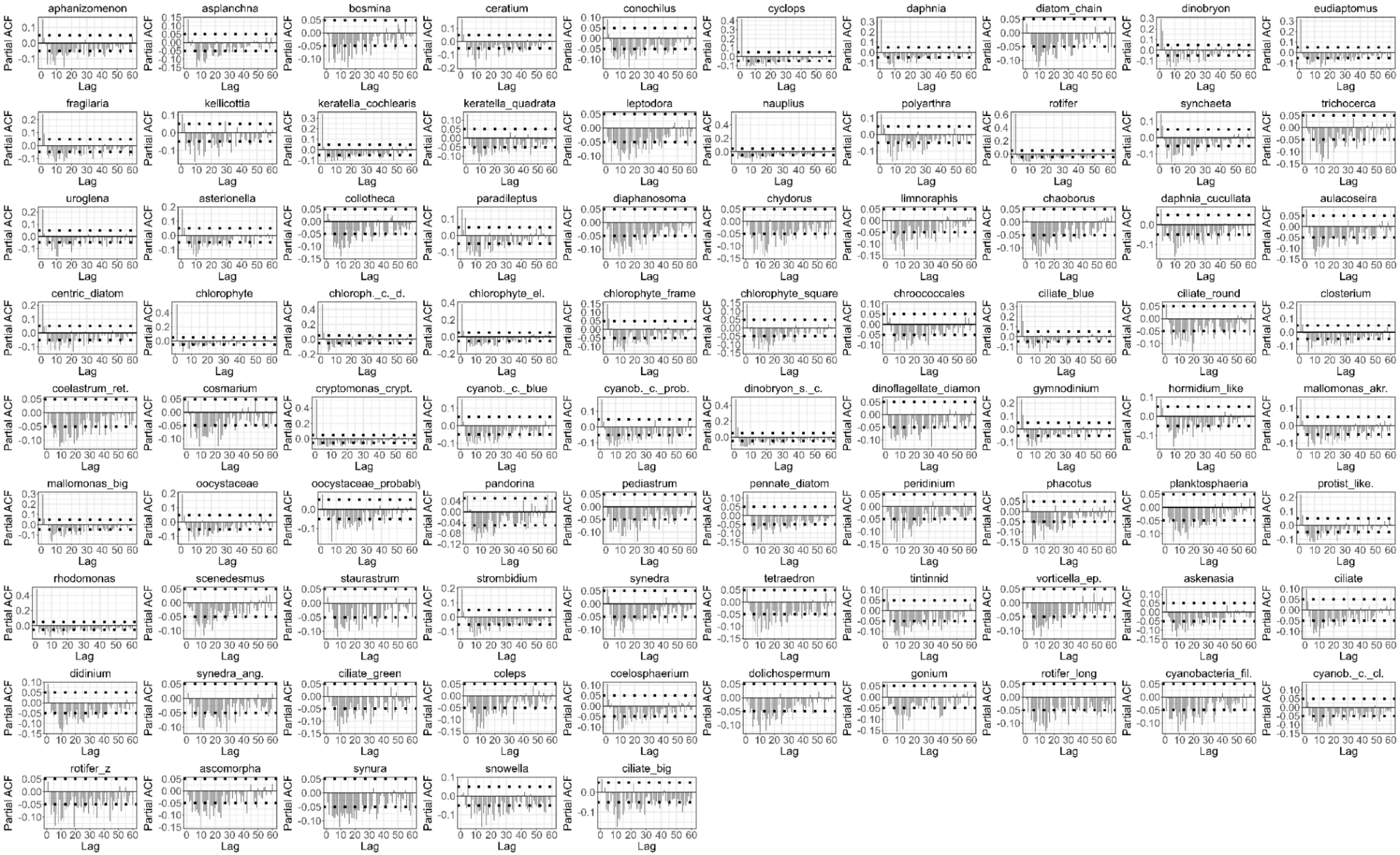

Fig. S20: Partial autocorrelation plots of the residuals obtained after smoothing the biotic variables using a loess function (span = 0.03) to reduce random noise in the raw data. The biotic variables represent individual taxa before their aggregation into broader taxonomic groups (see **Tables S1 and S2** for the groupings).

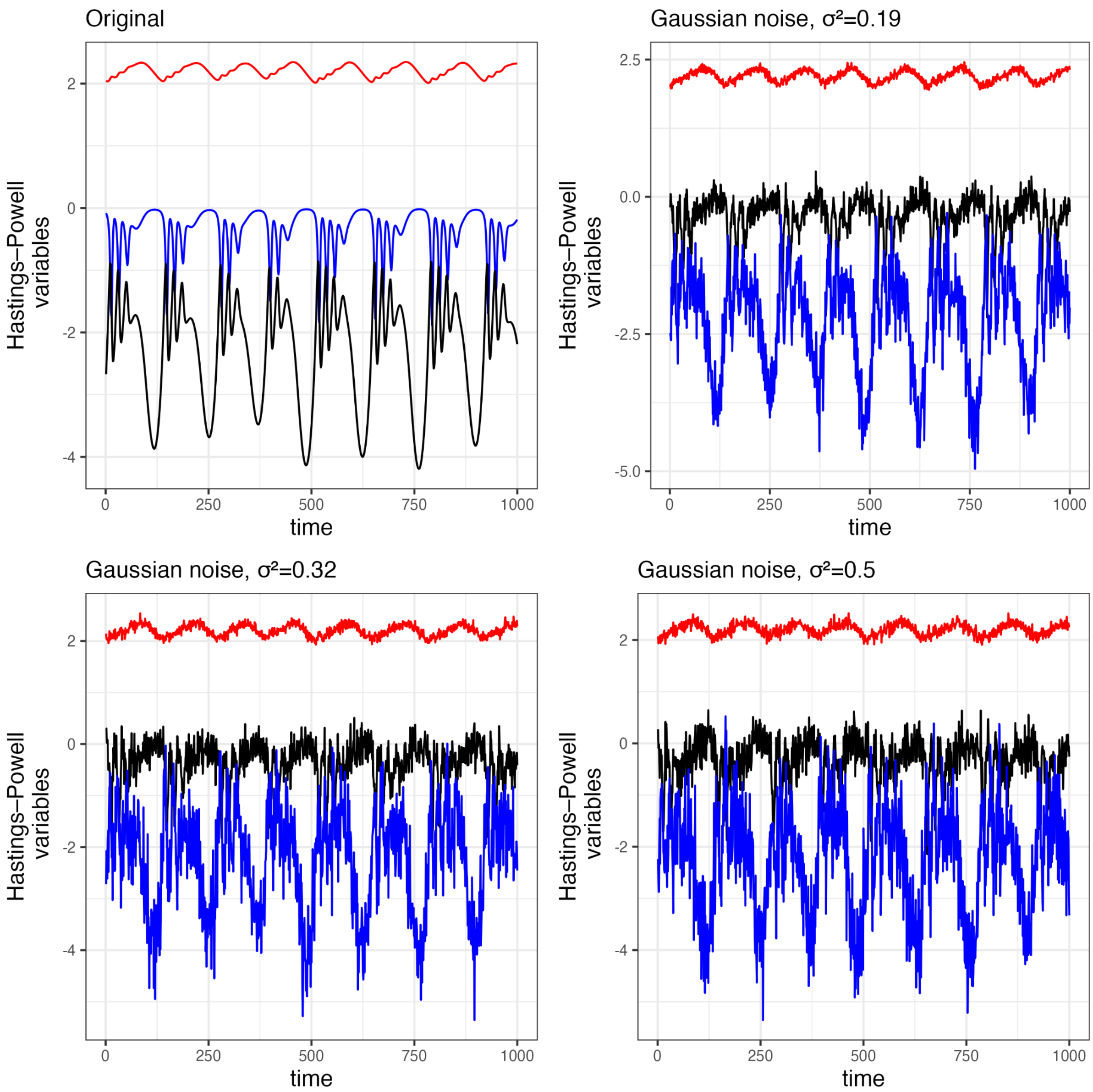

Fig. S21: Simulated Hastings–Powell time series with added measurement noise. Simulated time series from the three-species Hastings–Powell food-chain model. Panels show the original noise-free time series ("Original") and the same series after the addition of Gaussian noise with mean 0 and variance corresponding to the 25th (var = 0.19), 50th (var = 0.32), and 75th (var = 0.50) percentiles of the empirical variance estimated from the observational plankton data. Species are indicated by color: x (blue), y (black), and z (red). These time series illustrate the increasing noise levels used in the in-silico experiments prior to optional noise reduction via loess smoothing.

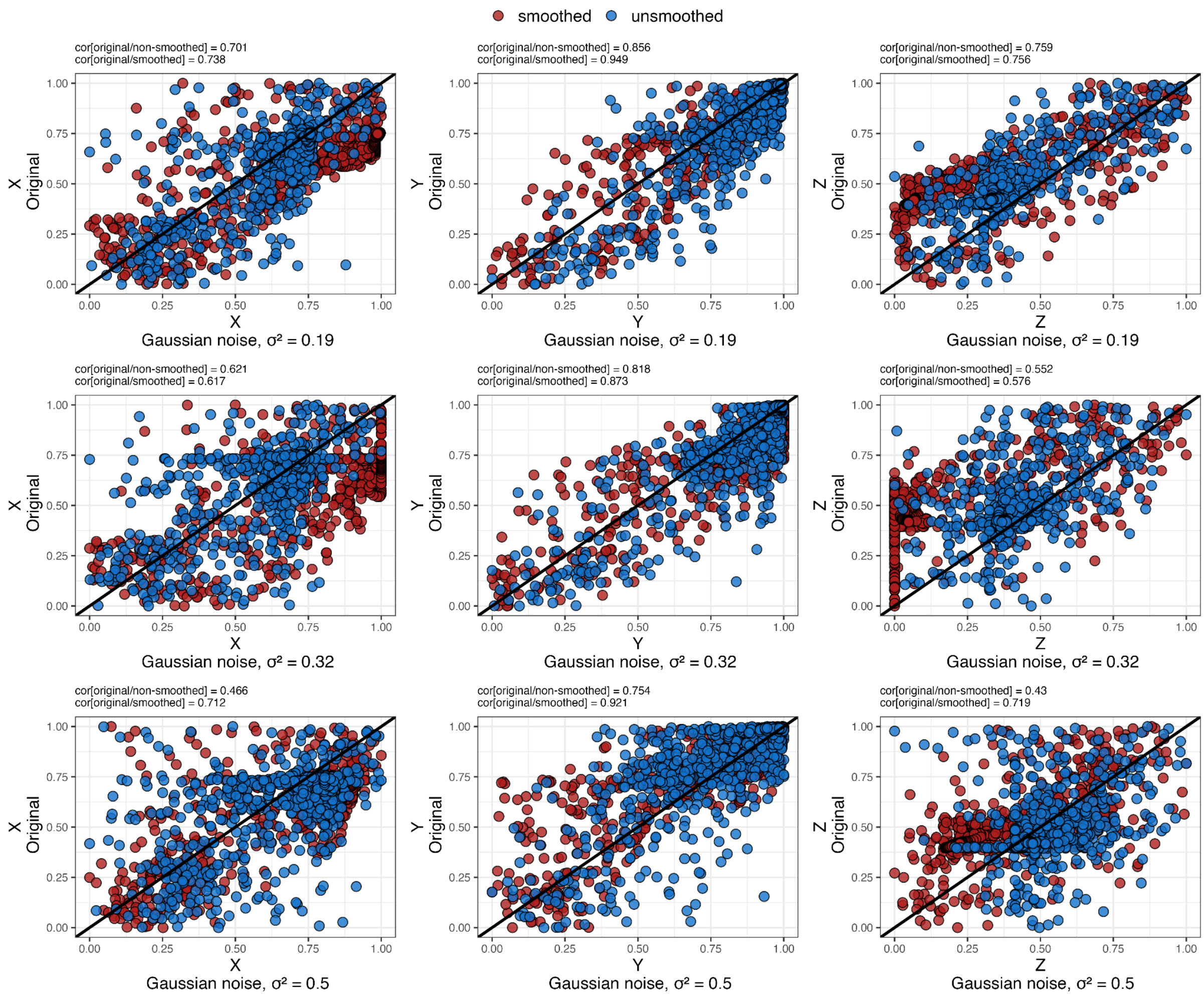

Fig. S22: Accuracy of inferred interaction coefficients for target variable x. Correlation of inferred S-map coefficients for the target variables x from the Hastings–Powell model. Each panel shows results from the best-performing S-map model (lowest RMSE). The x-axis displays the S-map coefficient for a given predictor variable in the noisy time series (with Gaussian noise added mean 0 and variance 0.19, 0.32, and 0.5), and the y-axis shows the corresponding coefficient from the original, noise-free time series. Colors indicate whether noise reduction via loess smoothing was applied (red = smoothed; blue = unsmoothed). Above each panel, the Spearman correlation coefficient between coefficients from the original time series and those from smoothed or unsmoothed noisy series is reported, indicating how well the smoothing preserves the true interactions. Coefficients are rescaled to the [0,1] range within each panel using min–max normalisation.

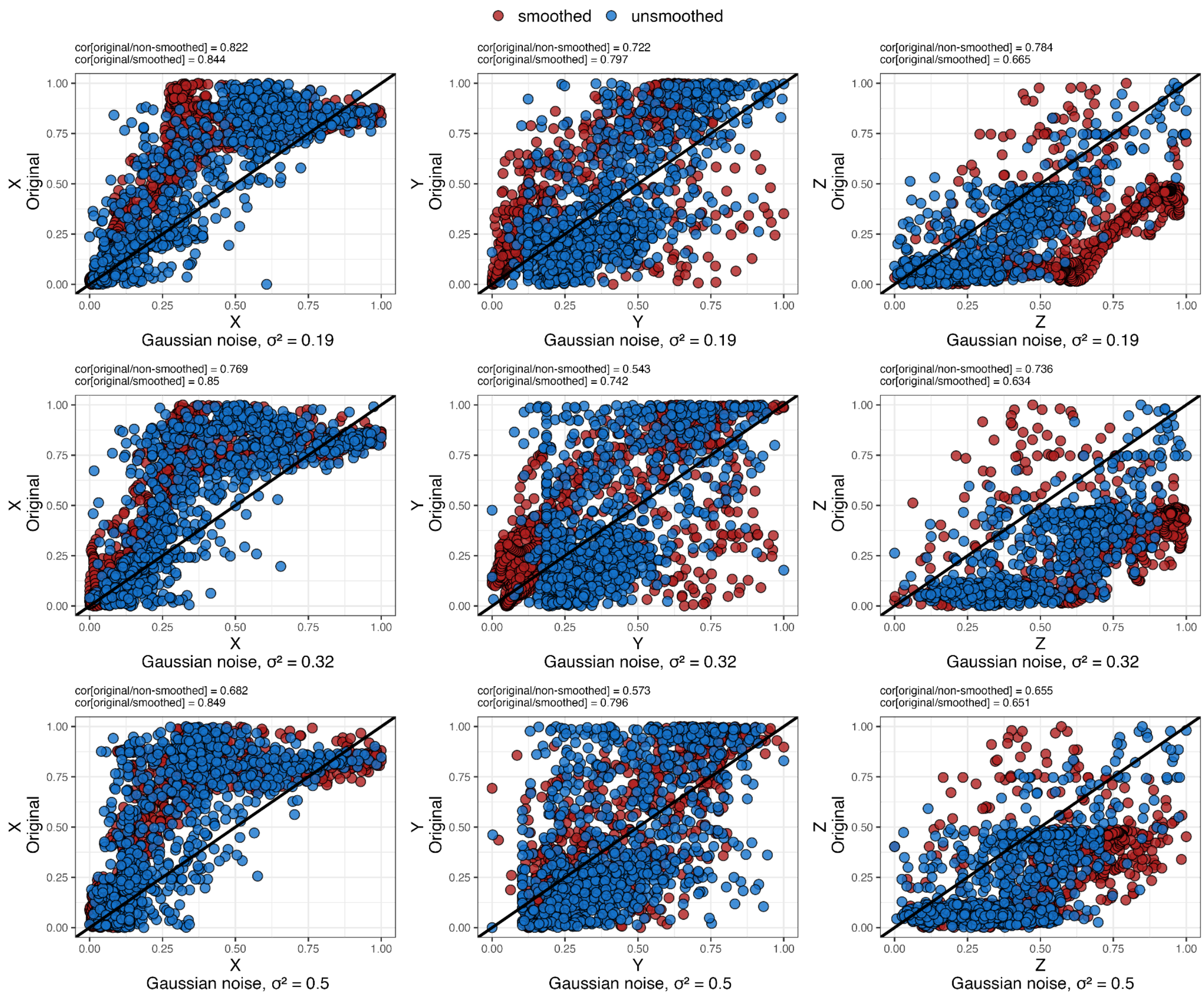

Fig. S23: Accuracy of inferred interaction coefficients for target variable y. Correlation of inferred S-map coefficients for the target variables y from the Hastings–Powell model. Each panel shows results from the best-performing S-map model (lowest RMSE). The x-axis displays the S-map coefficient for a given predictor variable in the noisy time series (with Gaussian noise added, mean 0 and variance 0.19, 0.32, and 0.5), and the y-axis shows the corresponding coefficient from the original, noise-free time series. Colors indicate whether noise reduction via loess smoothing was applied (red = smoothed; blue = unsmoothed). Above each panel, the Spearman correlation coefficient between coefficients from the original time series and those from smoothed or unsmoothed noisy series is reported, indicating how well the smoothing preserves the true interactions. Coefficients are rescaled to the [0,1] range within each panel using min–max normalisation.

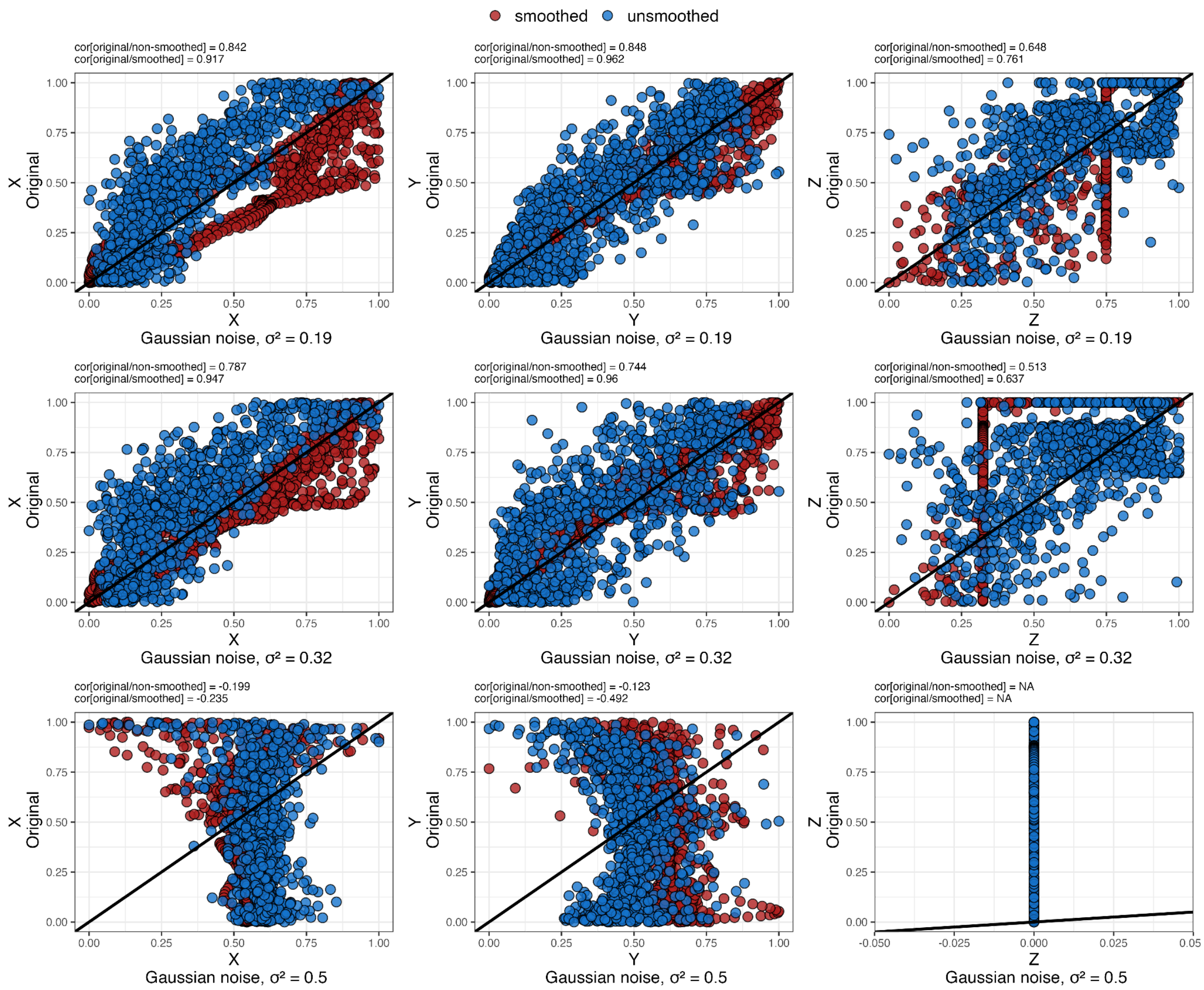

Fig. S24: Accuracy of inferred interaction coefficients for target variable z. Correlation of inferred S-map coefficients for the target variables z from the Hastings–Powell model. Each panel shows results from the best-performing S-map model (lowest RMSE). The x-axis displays the S-map coefficient for a given predictor variable in the noisy time series (with Gaussian noise added mean 0 and variance 0.19, 0.32, and 0.5), and the y-axis shows the corresponding coefficient from the original, noise-free time series. Colors indicate whether noise reduction via loess smoothing was applied (red = smoothed; blue = unsmoothed). Above each panel, the Spearman correlation coefficient between coefficients from the original time series and those from smoothed or unsmoothed noisy series is reported, indicating how well the smoothing preserves the true interactions. Coefficients are rescaled to the [0,1] range within each panel using min–max normalisation.

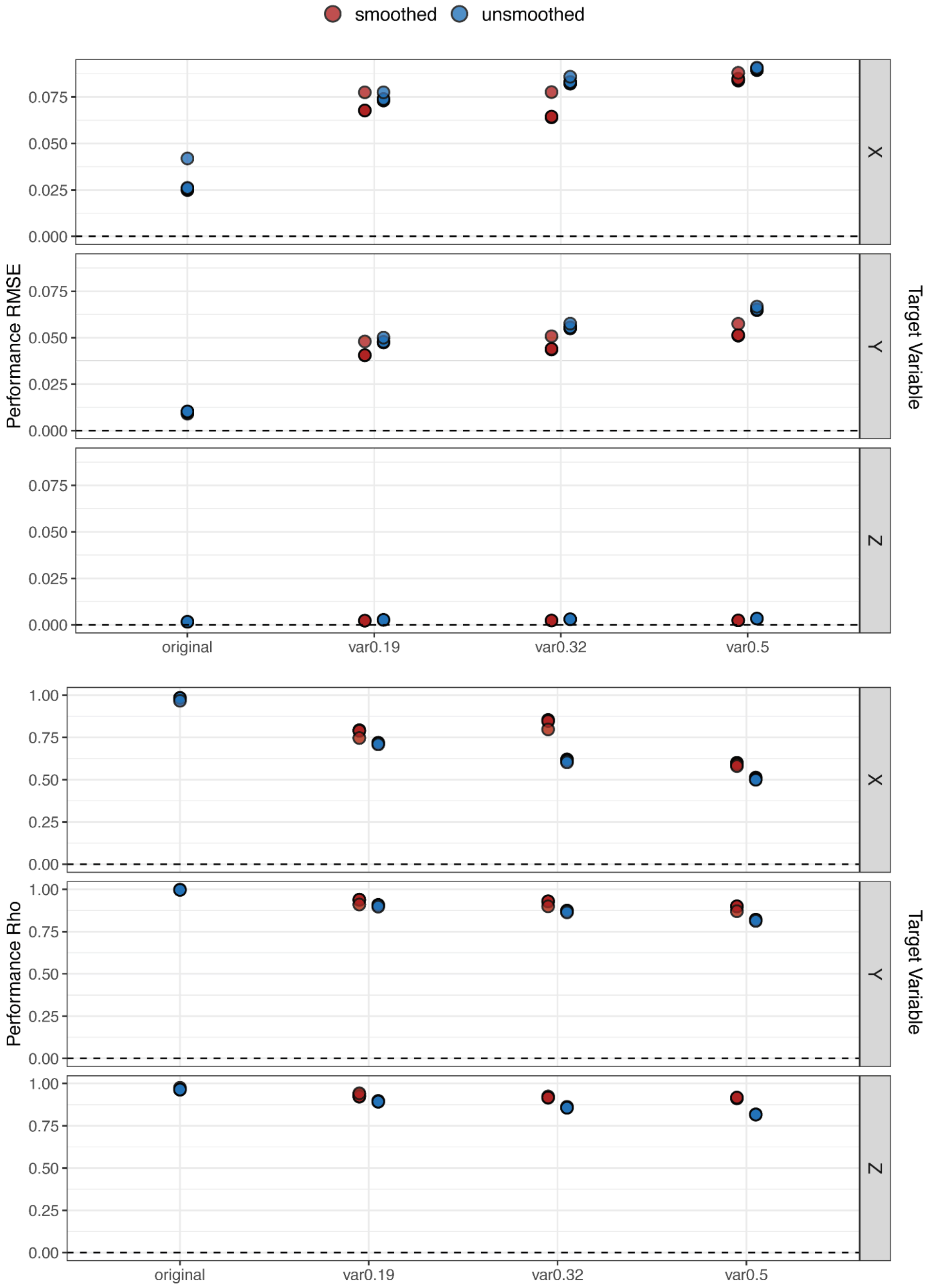

Fig. S25: Model performance comparison. Comparison of the top 5% of S-map models, assessed using RMSE (top panels) and Pearson correlation coefficient (Rho, bottom panels). Model performance was evaluated using sequential leave-future-out cross-validation. The x-axis indicates the level of Gaussian noise added to the time series (mean = 0, variance = var0.19, var0.32, var0.5 corresponding to the 25th, 50th, and 75th percentiles of the empirical variance, respectively), while "original" represents performance using the raw, unmodified time series. Rows correspond to the three target variables in the Hastings–Powell model (x, y, z). Colors indicate whether noise reduction via loess smoothing was applied (red = smoothed; blue = unsmoothed).

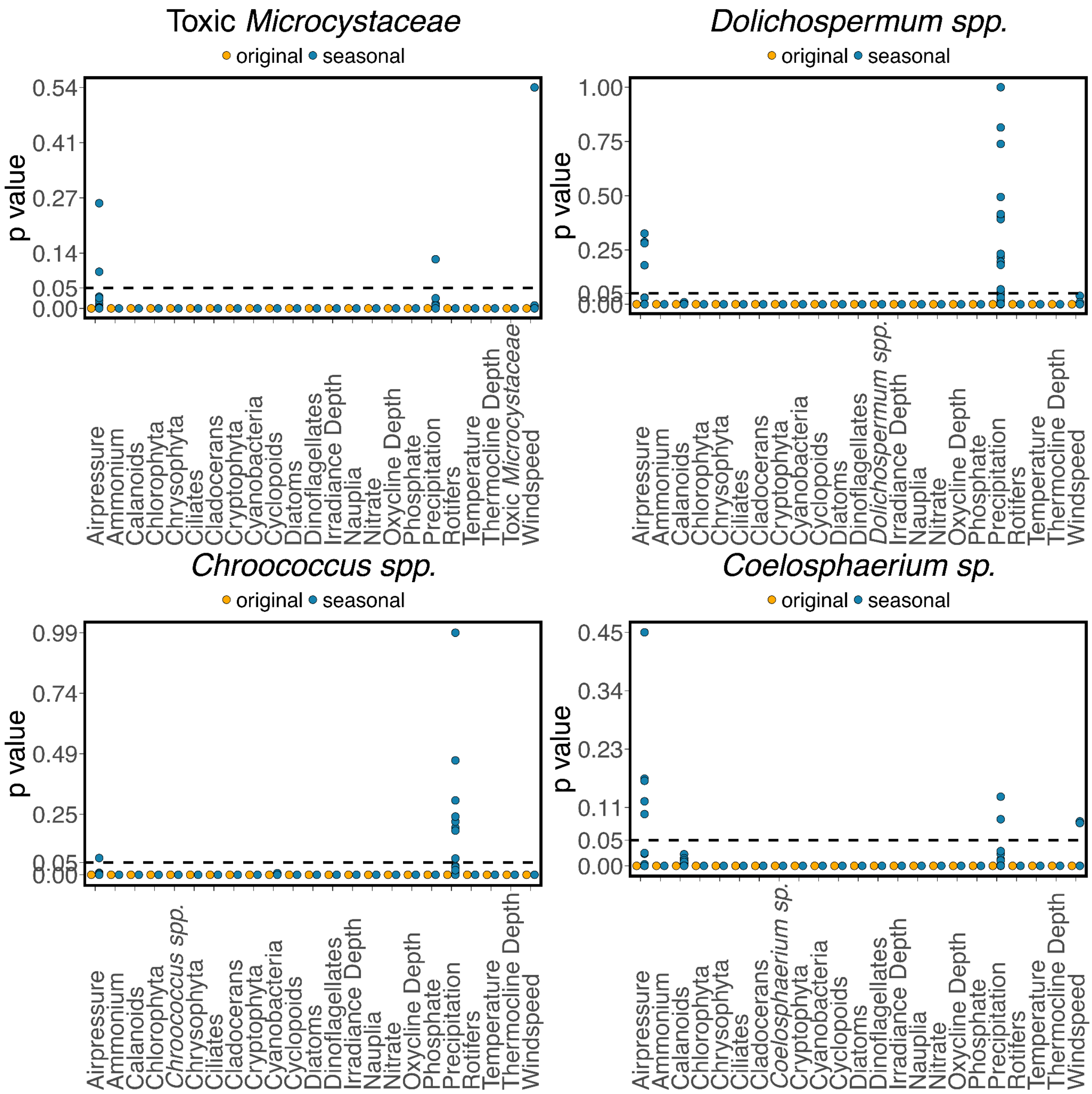

Fig. S26: CCM convergence test for cyanobacterial growth drivers. Convergence of cross-mapping forecasting skill (rho) was assessed for all target cyanobacteria and all predictor variables included in the S-map analysis. CCM was performed using an embedding dimension E = 10 and prediction horizon TP = −1. For each variable pair, rho was estimated at a minimum library size (10% of the time series) and a maximum library size (80% of the time series) using 100 random subsamples. Convergence was evaluated using a one-sided t-test, testing whether the mean rho at the maximum library size was greater than the mean rho at the minimum library size. P-values from the convergence test are shown in **orange** for the original time series (n=1) and in **blue** for the seasonal surrogate time series (n=100). The dashed horizontal line indicates the significance threshold (p = 0.05). All original time series and more than 87% of the seasonal surrogate time series exhibit p-values < 0.05, indicating significant convergence of cross-map skill with increasing library size.

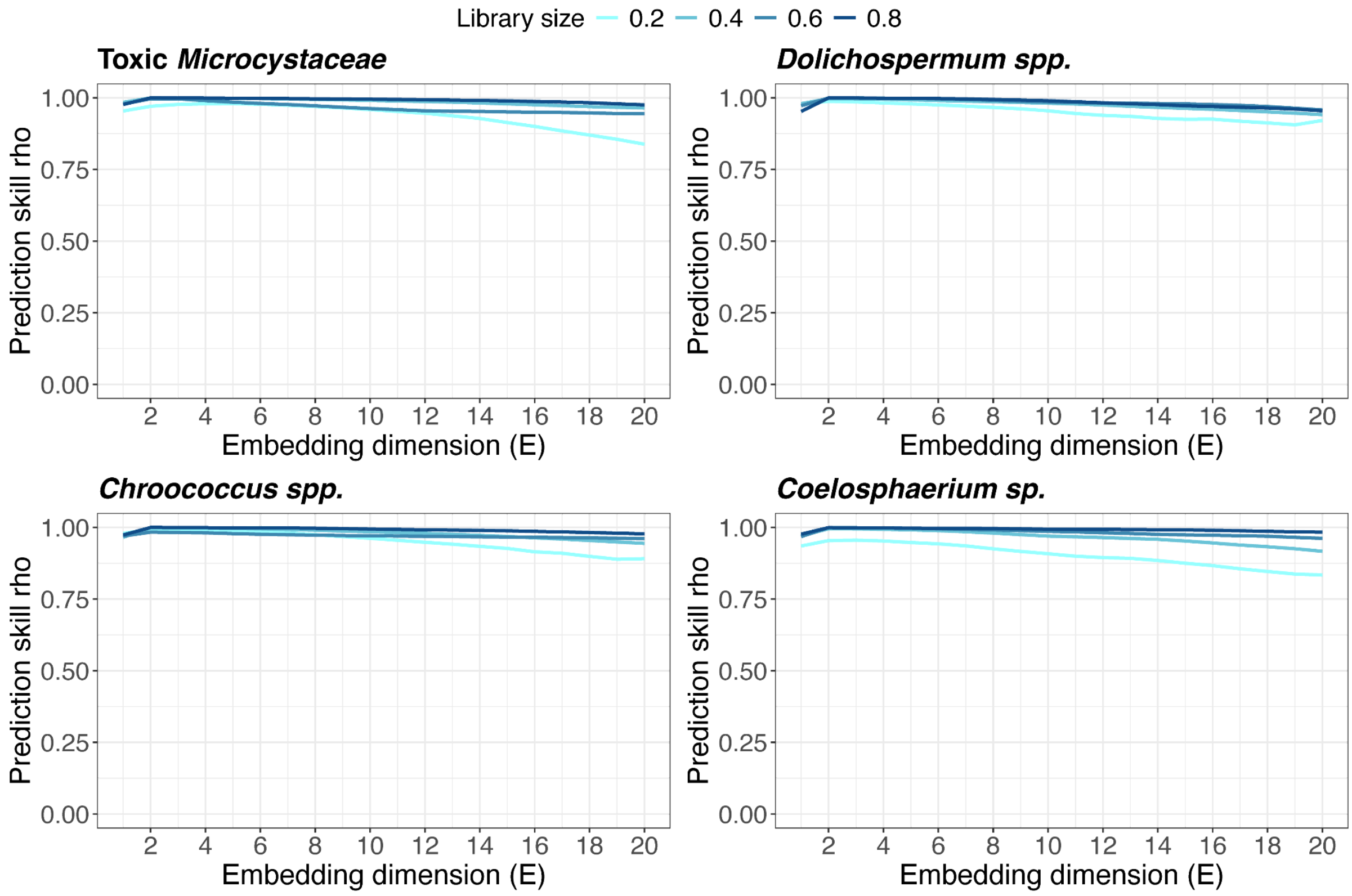

Fig. S27: Simplex projection results for all target cyanobacteria across embedding dimensions. Simplex projection was used to evaluate prediction skill across a range of embedding dimensions (E = 1–20). Models were fitted using different initial library sizes (20%, 40%, 60%, and 80% of the time series) and were used to predict the subsequent 20% of the dataset. The y-axis shows forecasting skill (rho, Pearson correlation coefficient), and the x-axis shows the tested embedding dimensions. Colours indicate the initial library size used for model fitting. Across all target cyanobacterial taxa, prediction skill remains relatively stable across embedding dimensions, indicating limited sensitivity to the choice of E.

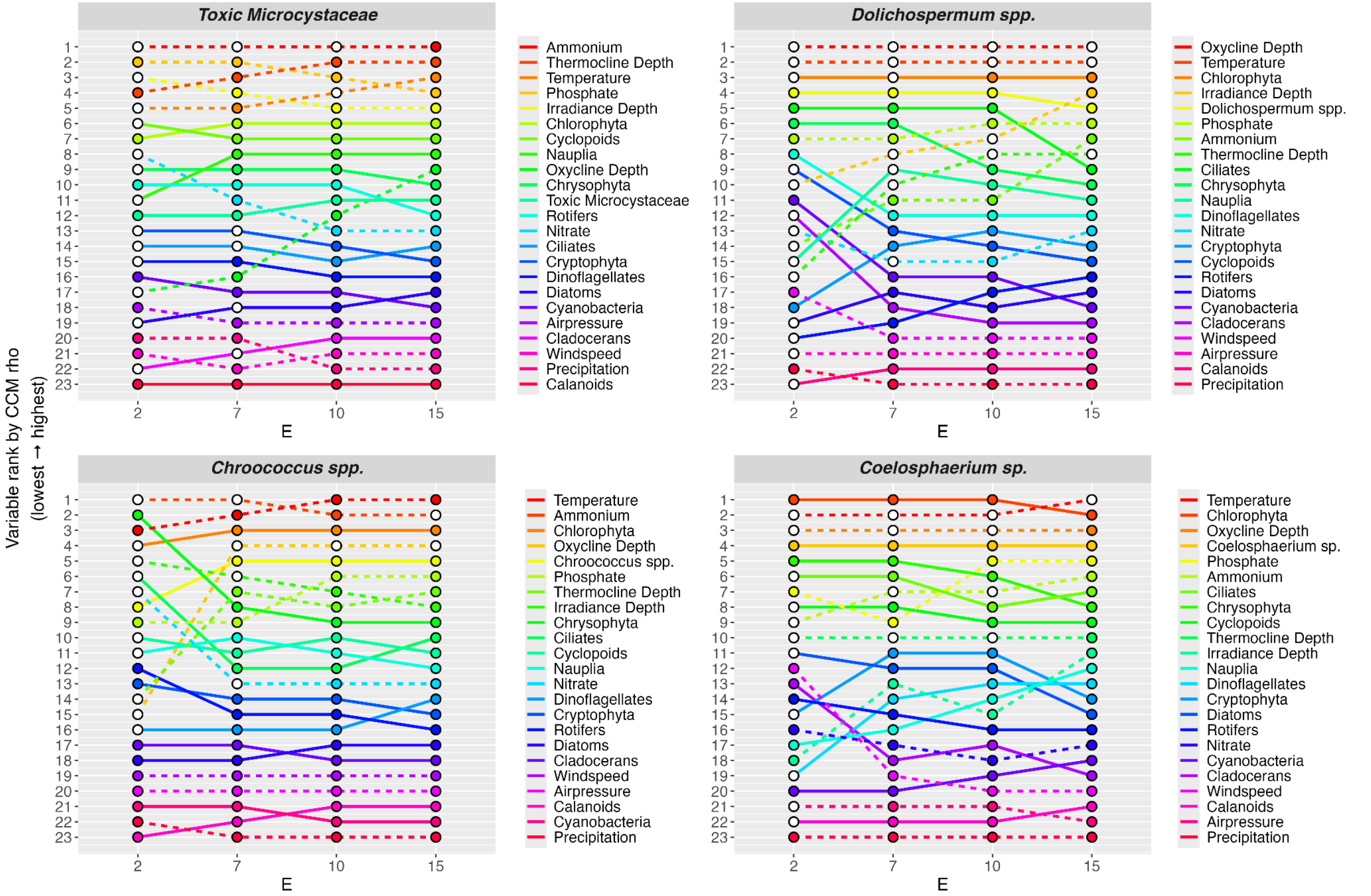

Fig. S28: Variable ranking across embedding dimensions using seasonal surrogate CCM. Rank dynamics of predictor variables across embedding dimensions (E = 2, 7, 10, 15) based on cross-mapping skill (rho) at maximum library size (80%). Each panel corresponds to one cyanobacterial target taxon. Within each panel, variables are ranked (y-axis; 1–23) from highest (rank 1) to lowest rho at each embedding dimension (x-axis). Colours denote the ranking of variables in the seasonal surrogate CCM at E = 15 and are held constant across embedding dimensions and across figures (**Fig. S29**) to facilitate comparison. Solid lines represent biotic variables; dashed lines represent abiotic variables. White symbols indicate variables not identified as causal at the daily scale (original-series rho < seasonal-surrogate rho, **see Methods section 3.1 Convergent Cross-Mapping**), whereas coloured symbols indicate variables classified as causal. Top-ranked variables remain largely stable across embedding dimensions (typically shifting by ≤1–2 ranks). In the seasonal surrogate analysis, highest-ranked predictors are predominantly abiotic variables, consistent with strong seasonal forcing. Note that at higher embedding dimensions (notably E = 15), an increased number of causally linked variables (more coloured symbols) is observed due to higher cross-mapping skill at larger E (**Figs. S30–S31**). This enhances the separation between original and seasonal surrogate signals and improves detection of weaker but ecologically meaningful interactions.

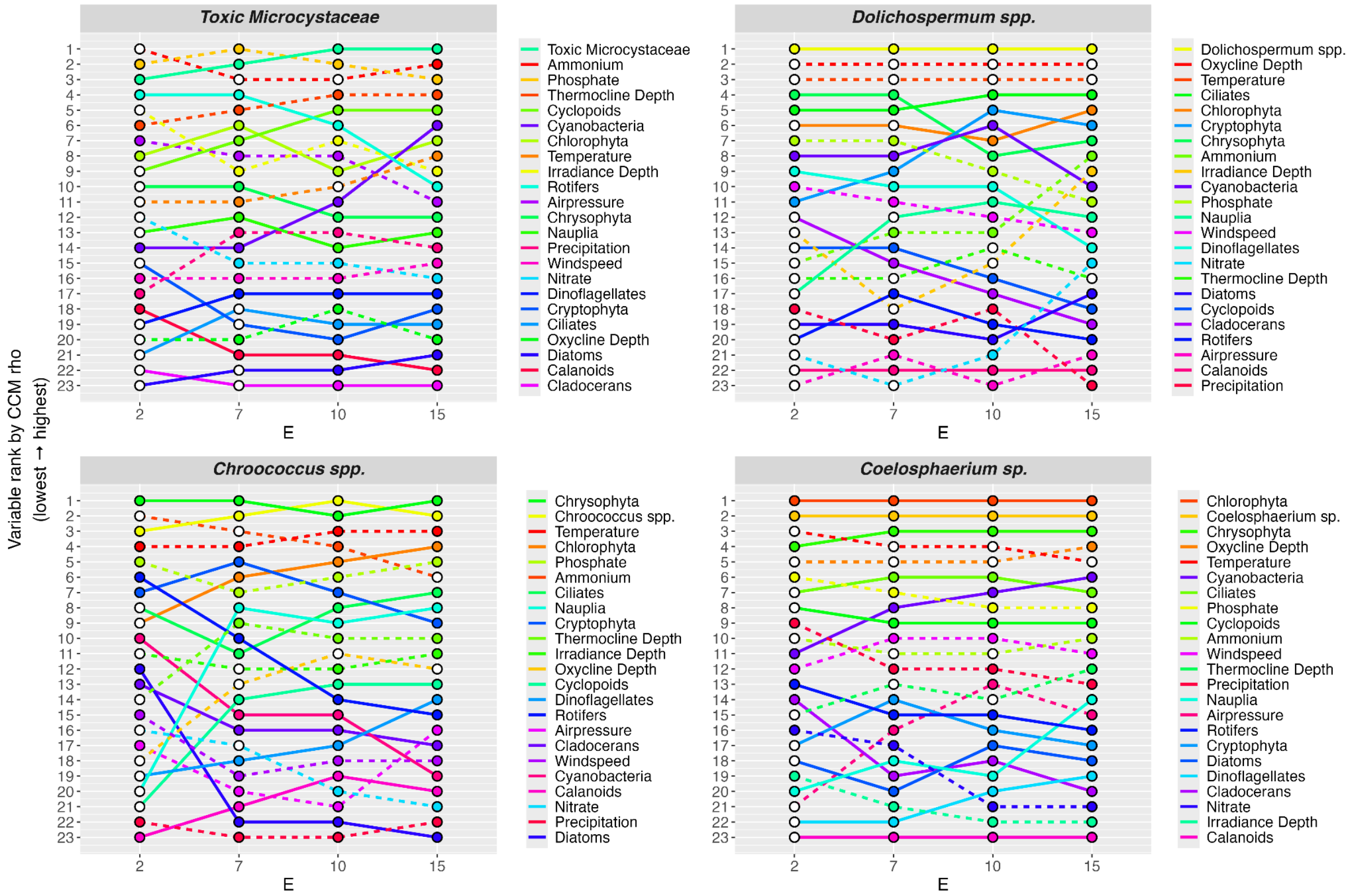

Fig. S29: Variable ranking across embedding dimensions using original time series CCM. Rank dynamics of predictor variables across embedding dimensions (E = 2, 7, 10, 15) based on cross-mapping skill (rho) at maximum library size (80%) for the original time series. Panels correspond to individual cyanobacterial target taxa. Variables are ranked (y-axis; 1–23) from highest to lowest ρ for each embedding dimension (x-axis). Colours correspond to the ranking derived from the seasonal surrogate CCM at E = 15, identical to **Fig. S28**, enabling direct comparison between seasonal and original analyses. Line types denote biotic (solid) and abiotic (dashed) variables. White symbols indicate non-causal variables at the daily scale, whereas coloured symbols denote variables meeting the causal criterion (original-series rho > seasonal-surrogate rho, **see Methods section 3.1 Convergent Cross-Mapping**). Across embedding dimensions, the relative position of top-ranked variables remains stable. In contrast to the seasonal surrogate results—where abiotic drivers dominate the highest ranks—the original time series reveals a mixture of biotic and abiotic predictors among the top ranks (often predominantly biotic), indicating causal influences on daily cyanobacterial growth beyond seasonal structure. Note that at higher embedding dimensions (notably E = 15), an increased number of causally linked variables (more coloured symbols) is observed due to higher cross-mapping skill at larger E (**Figs. S30–S31**). This enhances the separation between original and seasonal surrogate signals and improves detection of weaker but ecologically meaningful interactions.

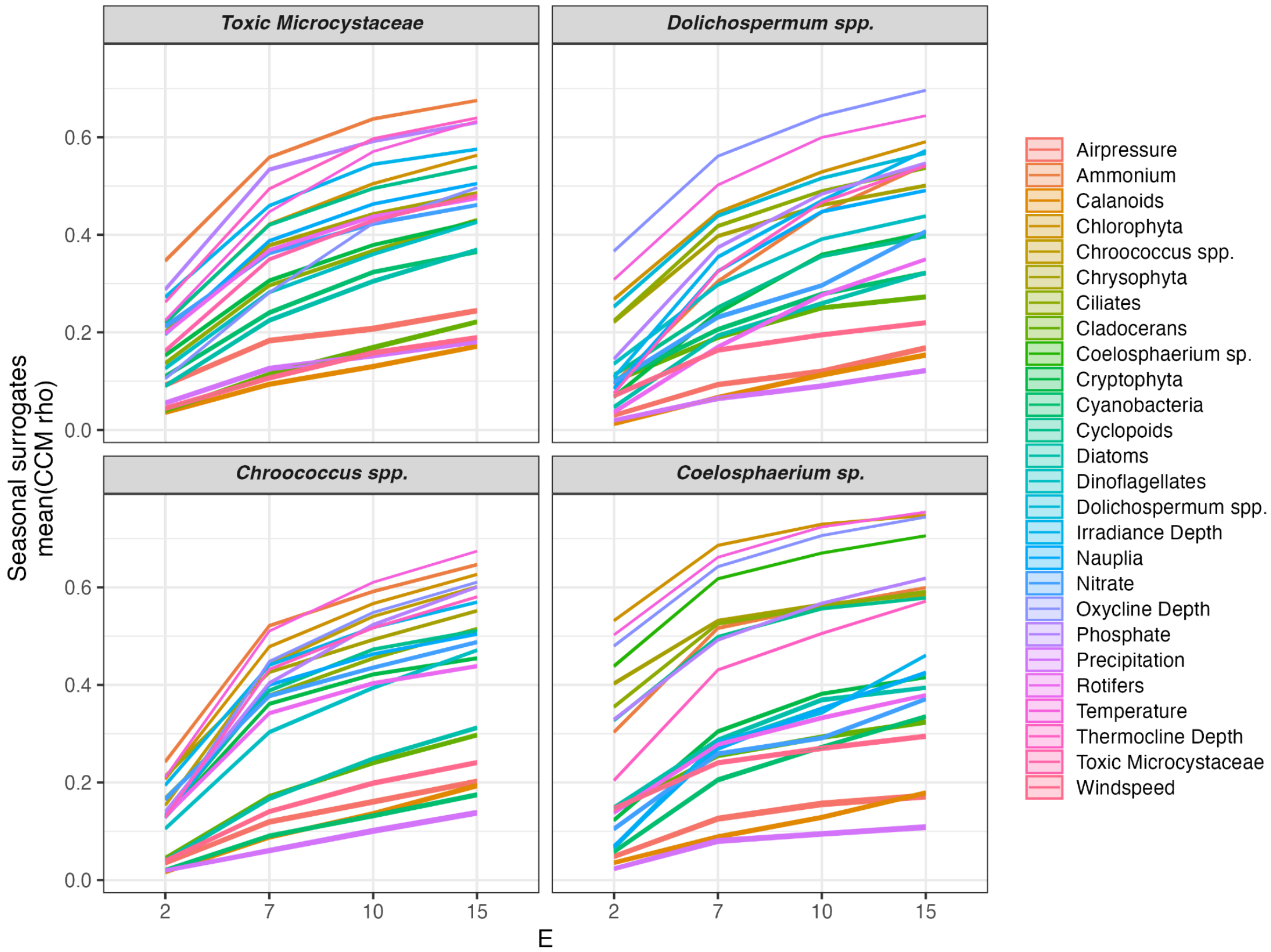

Fig. S30: CCM forecasting skill across embedding dimensions (seasonal surrogate time series). Mean cross-mapping skill (rho) at maximum library size (80%) across embedding dimensions (E) for each predictor variable. Panels correspond to individual cyanobacterial target taxa. The y-axis shows mean rho, and the x-axis shows embedding dimension. Colours identify predictor variables included in the CCM analysis. Shaded bands represent ±1 s.e. across n=100 seasonal surrogate time series. Across taxa and variables, mean rho generally increases with embedding dimension.

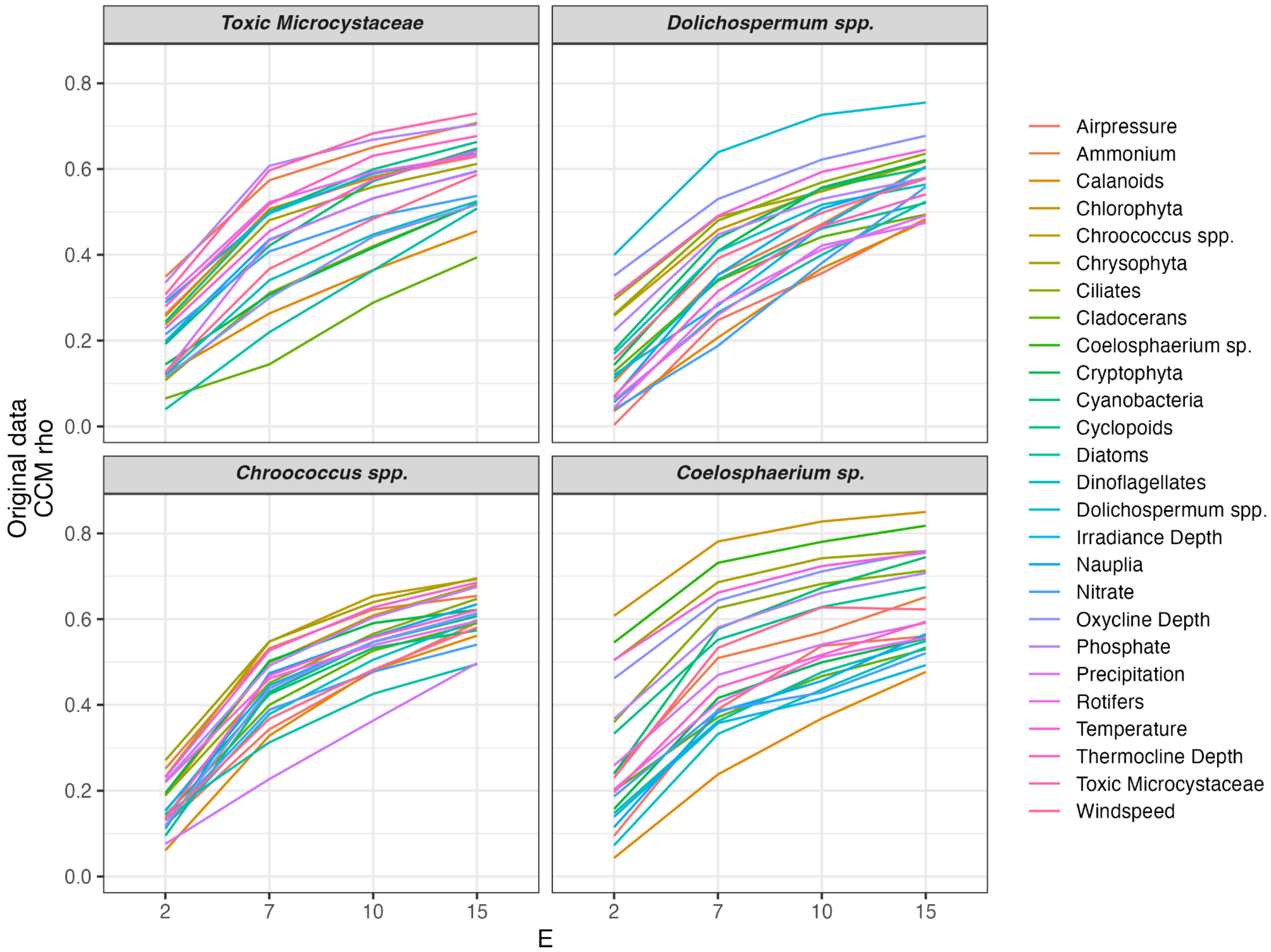

Fig. S31: CCM forecasting skill across embedding dimensions (original time series). Cross-mapping skill (rho) at maximum library size (80%) across embedding dimensions (E) for each predictor variable in the original time series. Panels correspond to individual cyanobacterial target taxa. The y-axis shows mean rho, and the x-axis shows embedding dimension. Colours identify predictor variables tested in the CCM analysis. As in the seasonal surrogate analysis, mean rho generally increases with embedding dimension across taxa and predictors, indicating consistent scaling of cross-mapping skill with E.

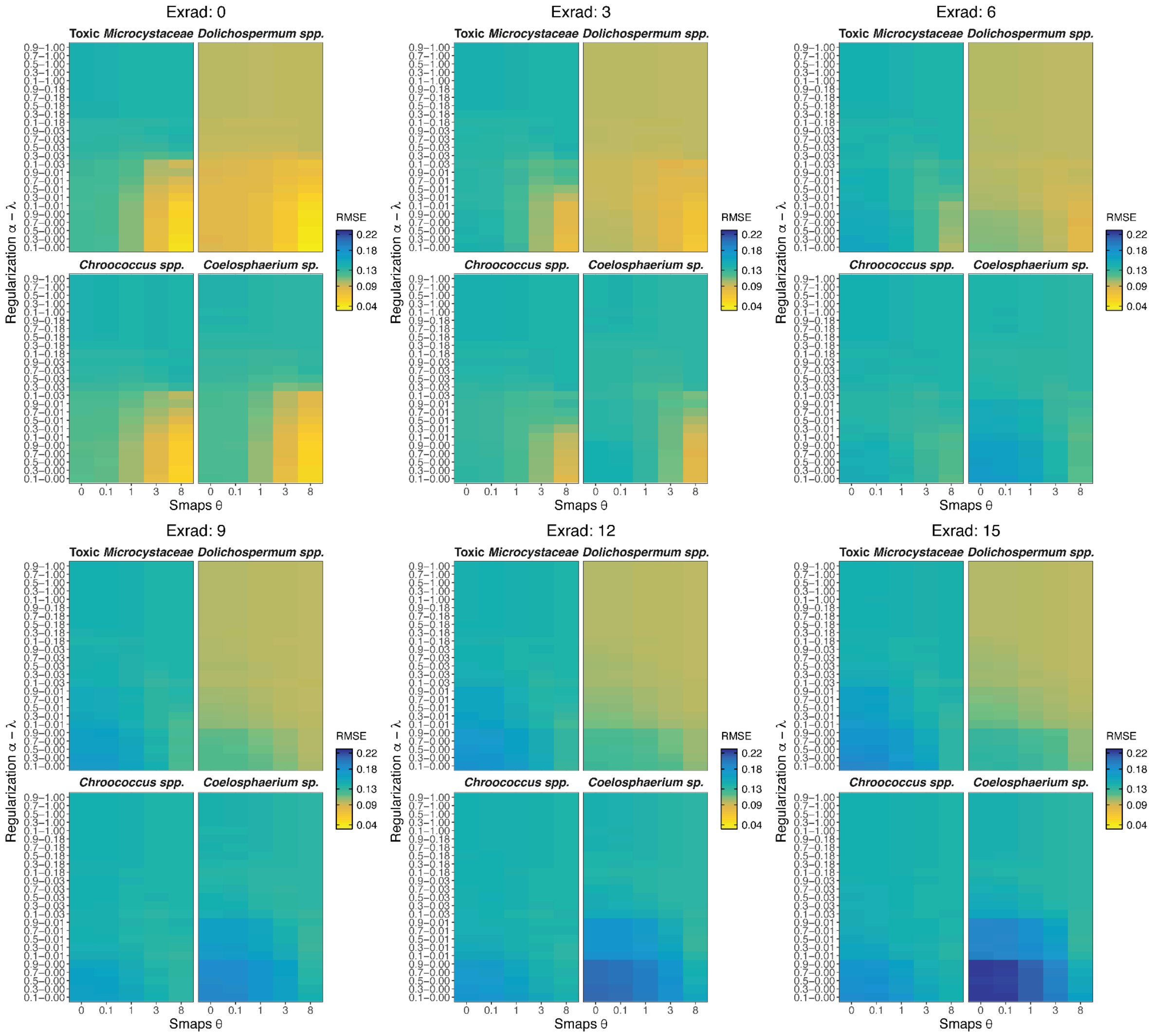

Fig. S32: Performance of MDR S-maps across all 125 parameter combinations (θ, α, λ) for each target cyanobacterial net growth rate, accounting for temporal autocorrelation via the exclusion radius (exrad). Colours represent predictive skill as measured by root mean square error (RMSE) from leave-future-out cross-validation, with warmer colours (e.g., yellow) indicating higher predictive accuracy and cooler colours (e.g., blue) indicating lower accuracy. The exclusion radius (exrad) corresponds to the number of temporally adjacent points removed to control for autocorrelation. The prediction horizon is set at 1 day. See Materials and Methods for a detailed explanation of the model parameters and the implementation of the cross-validation analysis.

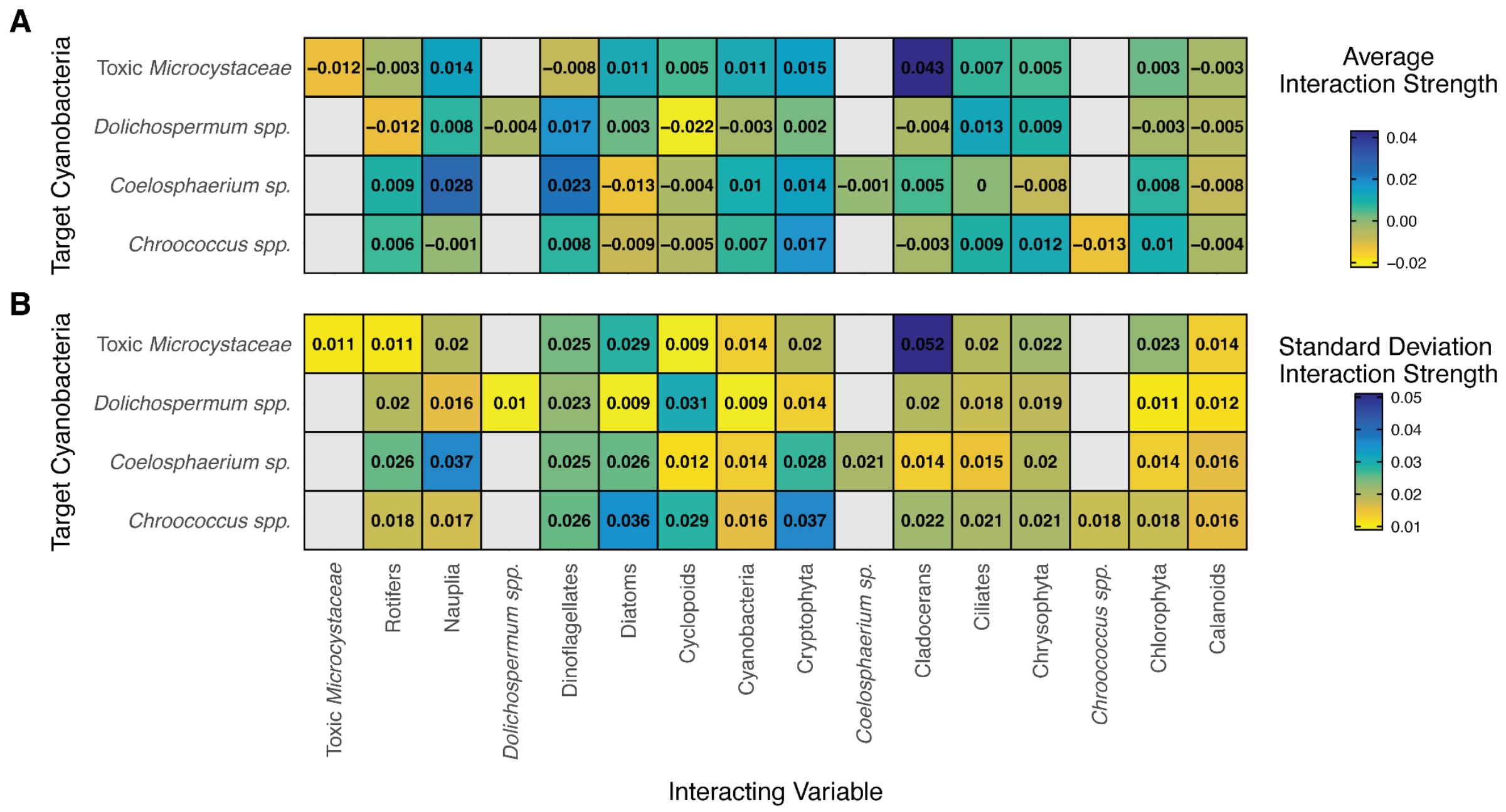

Fig. S33: Interaction strengths between target cyanobacteria taxa and biotic predictors estimated from regularised multiview S-map models. Interaction strengths were inferred as the time-average of the interaction coefficients for the four target cyanobacteria (Toxic *Microcystaceae*, *Dolichospermum spp., Coelosphaerium sp.,* and *Chroococcus spp.*) using the best-performing regularised multiview S-map (MDR S-map) model ($\theta = 8$, $\alpha = 0.1$, $\lambda = 0.001$). Biotic predictors included six zooplankton groups, six phytoplankton groups, and the density of each target cyanobacterial taxon. Panels show **(A)** the time-average interaction coefficient and **(B)** the standard deviation of n=1512 coefficient estimates over time.

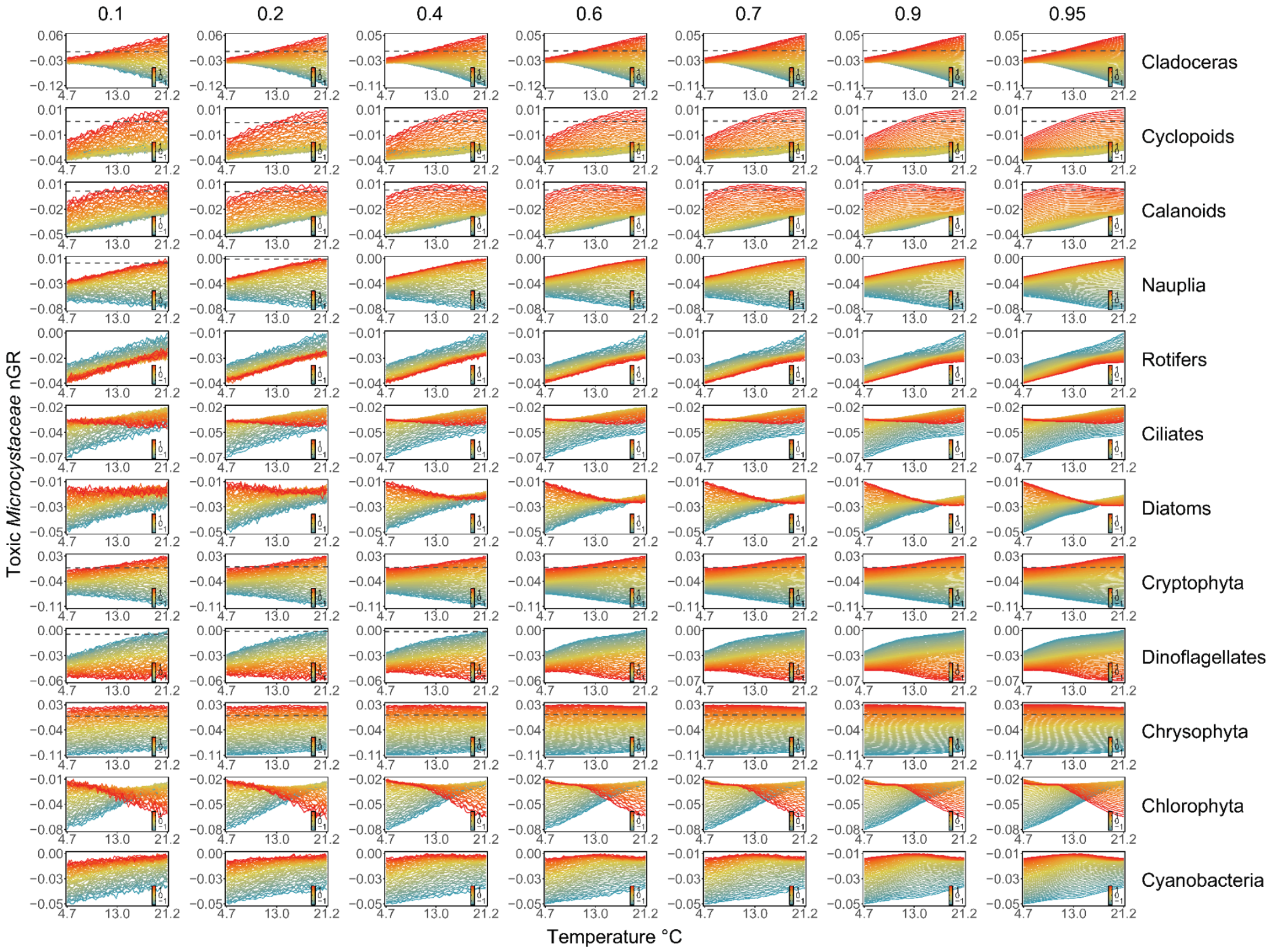

Fig. S34: Sensitivity analysis of Toxic *Microcystaceae* net growth rate to temperature across different dataset sizes. Response functions of Toxic *Microcystaceae* net growth rate (nGR) (Ln(ind/mL) · day^{-1}) to temperature (°C) across different biotic interactions (rows) and data subset sizes (columns). Each panel represents a biotic interaction, with temperature on the x-axis and changes in nGR on the y-axis. Columns correspond to different proportions of the dataset used (10%–95%) to assess response function robustness. Lines show mean estimated responses from n=50 subsamples (see Materials and Methods) and colour indicates the scaled density of the biotic factor in Ln(ind/mL). Patterns remain consistent across data lengths, confirming robustness.

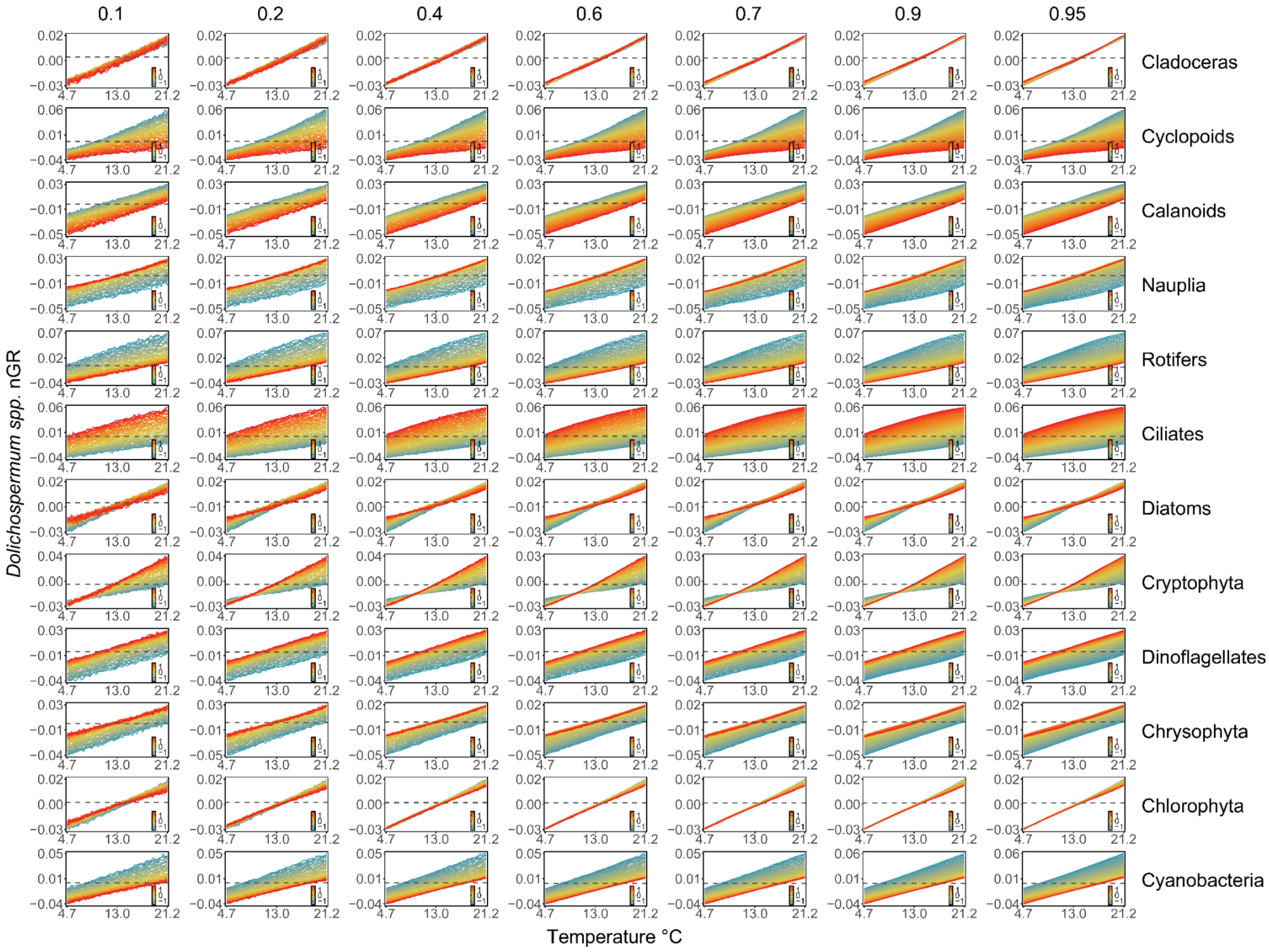

Fig. S35: Sensitivity analysis of *Dolichospermum spp.* net growth rate to temperature across different dataset sizes. Response functions of *Dolichospermum* net growth rate (nGR) (Ln(ind/mL) · day^{-1}) to temperature (°C) across different biotic interactions (rows) and data subset sizes (columns). Each panel represents a biotic interaction, with temperature on the x-axis and changes in nGR on the y-axis. Columns correspond to different proportions of the dataset used (10%–95%) to assess response function robustness. Lines show mean estimated responses from n=50 subsamples (see Materials and Methods) and colour indicates the scaled density of the biotic factor in Ln(ind/mL). Patterns remain consistent across data lengths, confirming robustness.

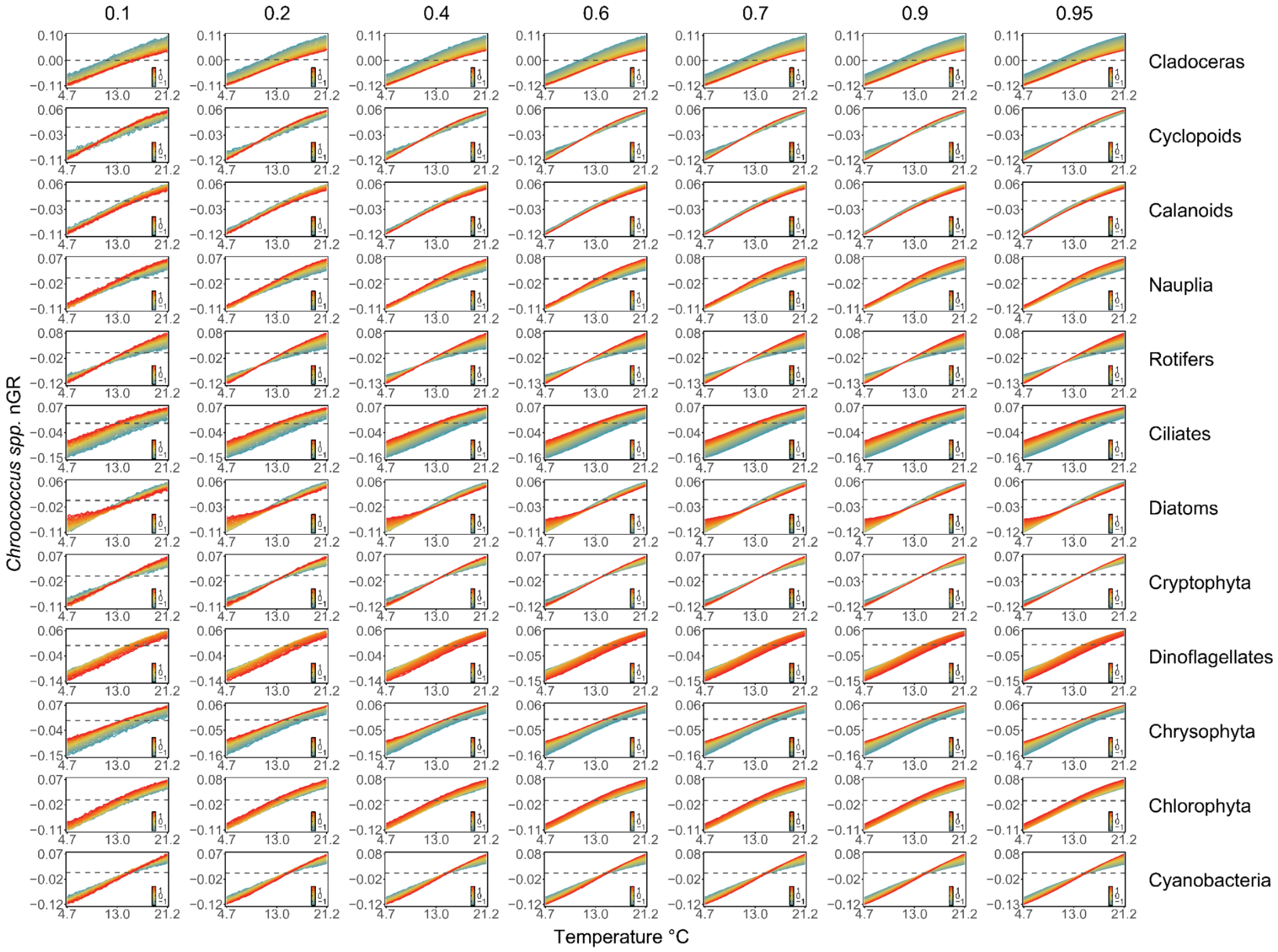

Fig. S36: Sensitivity analysis of *Chroococcus spp.* net growth rate to temperature across different dataset sizes. Response functions of *Chroococcus* net growth rate (nGR) (Ln(ind/mL)·day^{-1}) to temperature (°C) across different biotic interactions (rows) and data subset sizes (columns). Each panel represents a biotic interaction, with temperature on the x-axis and changes in nGR on the y-axis. Columns correspond to different proportions of the dataset used (10%–95%) to assess response function robustness. Lines show mean estimated responses from n=50 subsamples (see Materials and Methods) and colour indicates the scaled density of the biotic factor in Ln(ind/mL). Patterns remain consistent across data lengths, confirming robustness.

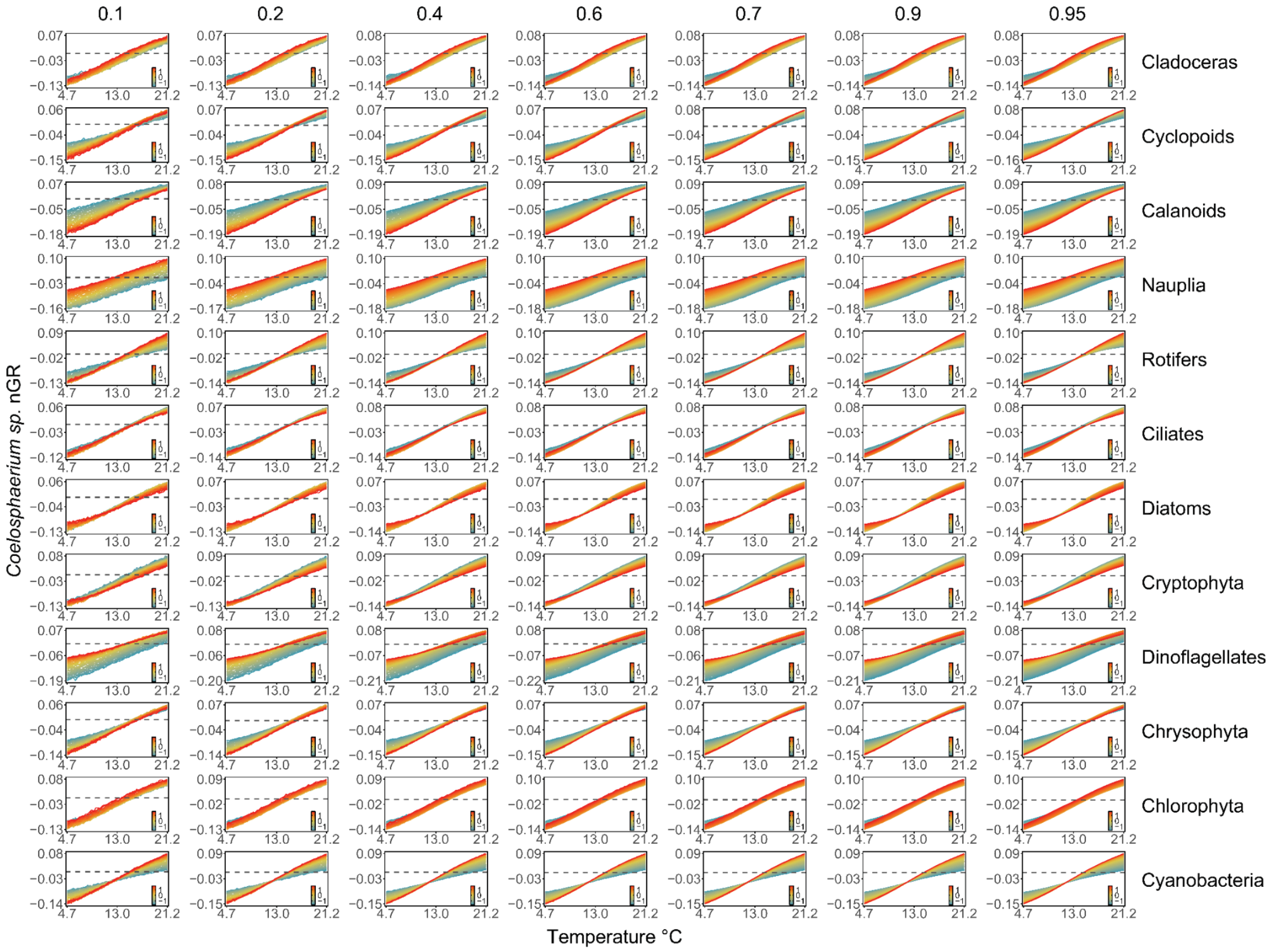

Fig. S37: Sensitivity analysis of *Coelosphaerium sp.* net growth rate to temperature across different dataset sizes. Response functions of *Coelosphaerium* net growth rate (nGR) (Ln(ind/mL) · day^{-1}) to temperature (°C) across different biotic interactions (rows) and data subset sizes (columns). Each panel represents a biotic interaction, with temperature on the x-axis and changes in nGR on the y-axis. Columns correspond to different proportions of the dataset used (10%–95%) to assess response function robustness. Lines show mean estimated responses from n=50 subsamples (see Materials and Methods) and colour indicates the scaled density of the biotic factor in Ln(ind/mL). Patterns remain consistent across data lengths, confirming robustness.

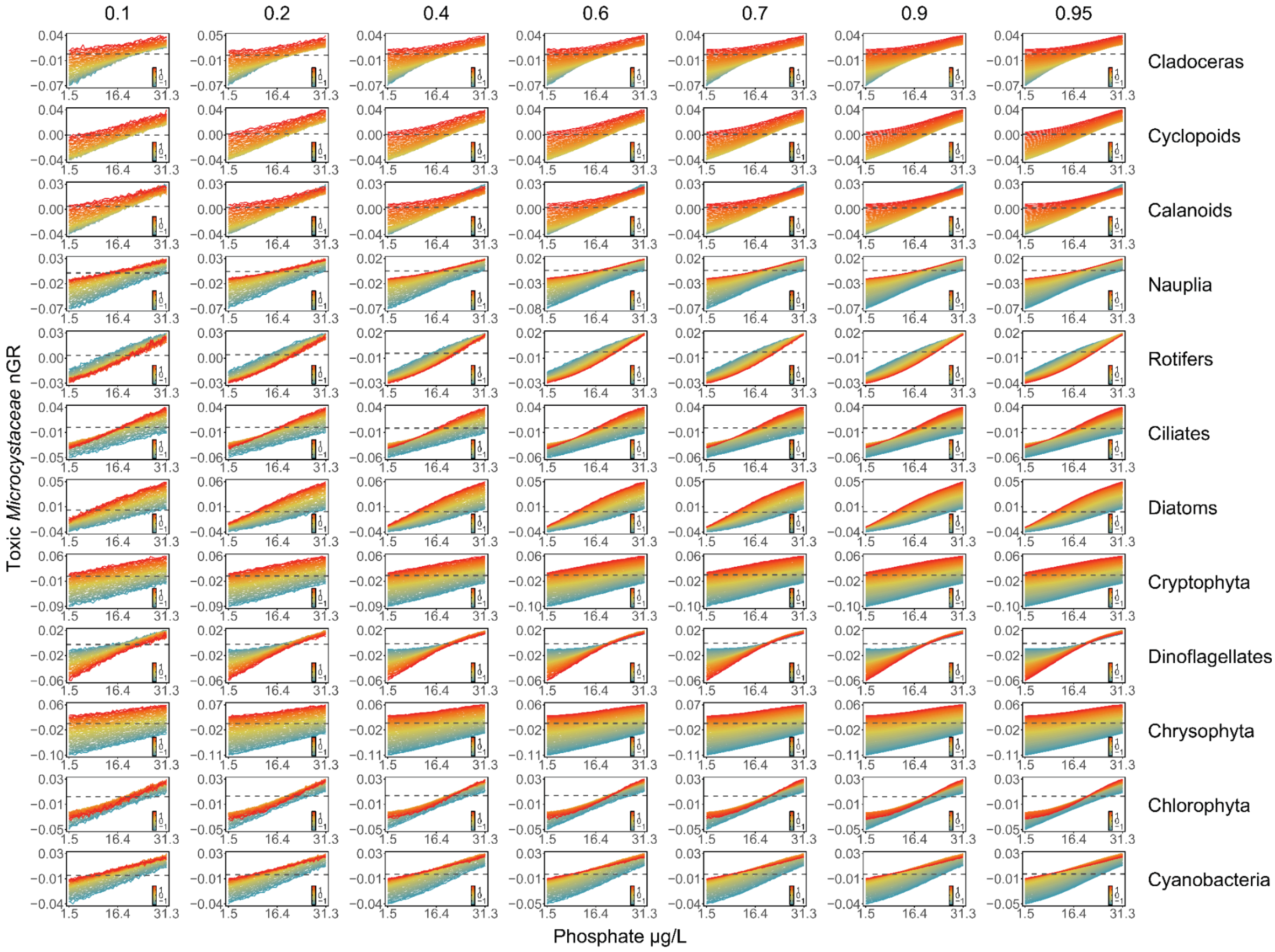

Fig. S38: Sensitivity analysis of Toxic *Microcystaceae* net growth rate to phosphate across different dataset sizes. Response functions of Toxic *Microcystaceae* net growth rate (nGR) (Ln(ind/mL) · day^{-1}) to phosphate (PO_4^{3-}, µg/L) across different biotic interactions (rows) and data subset sizes (columns). Each panel represents a biotic interaction, with phosphate on the x-axis and changes in nGR on the y-axis. Columns correspond to different proportions of the dataset used (10%–95%) to assess response function robustness. Lines show mean estimated responses from n=50 subsamples (see Materials and Methods) and colour indicates the scaled density of the biotic factor in Ln(ind/mL). Patterns remain consistent across data lengths, confirming robustness.

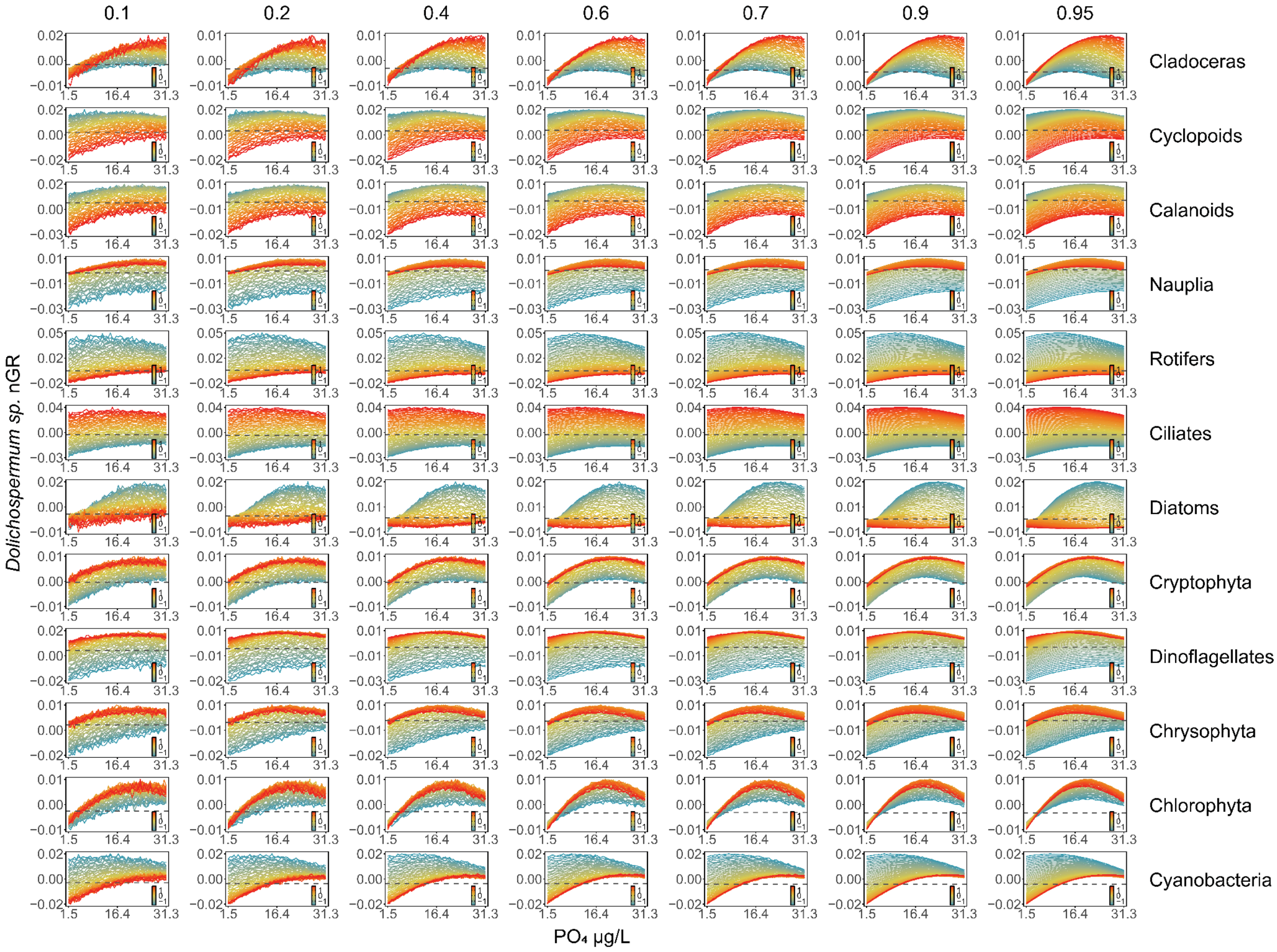

Fig. S39: Sensitivity analysis of *Dolichospermum spp.* net growth rate to phosphate across different dataset sizes. Response functions of *Dolichospermum* net growth rate (nGR) (Ln(ind/mL)·day^{-1}) to phosphate (PO_4^{3-}, µg/L) across different biotic interactions (rows) and data subset sizes (columns). Each panel represents a biotic interaction, with phosphate on the x-axis and changes in nGR on the y-axis. Columns correspond to different proportions of the dataset used (10%–95%) to assess response function robustness. Lines show mean estimated responses from n=50 subsamples (see Materials and Methods) and colour indicates the scaled density of the biotic factor in Ln(ind/mL). Patterns remain consistent across data lengths, confirming robustness.

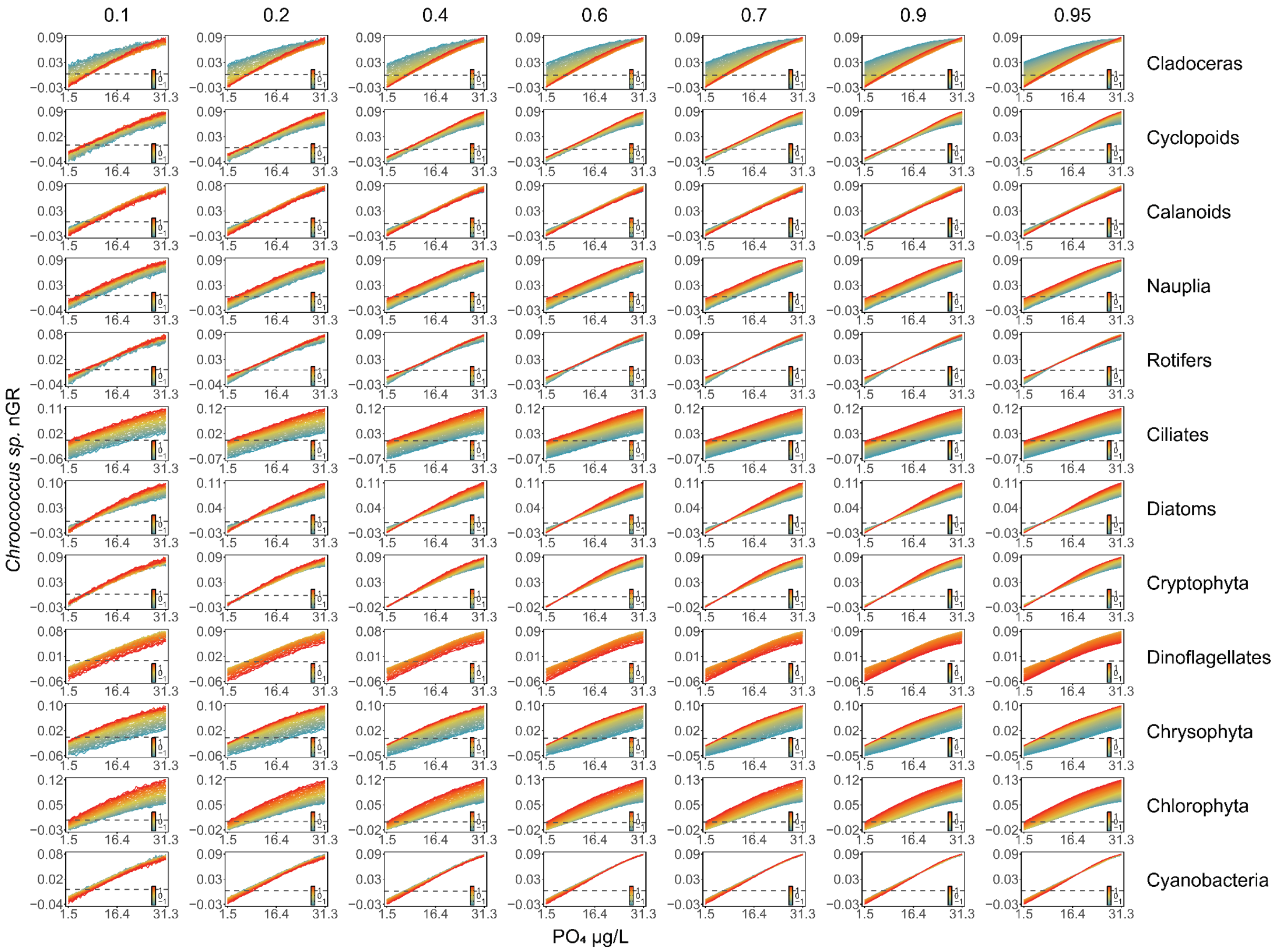

Fig. S40: Sensitivity analysis of *Chroococcus spp.* net growth rate to phosphate across different dataset sizes. Response functions of *Chroococcus* net growth rate (nGR) (Ln(ind/mL) · day^{-1}) to phosphate (PO_4^{3-}, µg/L) across different biotic interactions (rows) and data subset sizes (columns). Each panel represents a biotic interaction, with phosphate on the x-axis and changes in nGR on the y-axis. Columns correspond to different proportions of the dataset used (10%–95%) to assess response function robustness. Lines show mean estimated responses from n=50 subsamples (see Materials and Methods) and colour indicates the scaled density of the biotic factor in Ln(ind/mL). Patterns remain consistent across data lengths, confirming robustness.

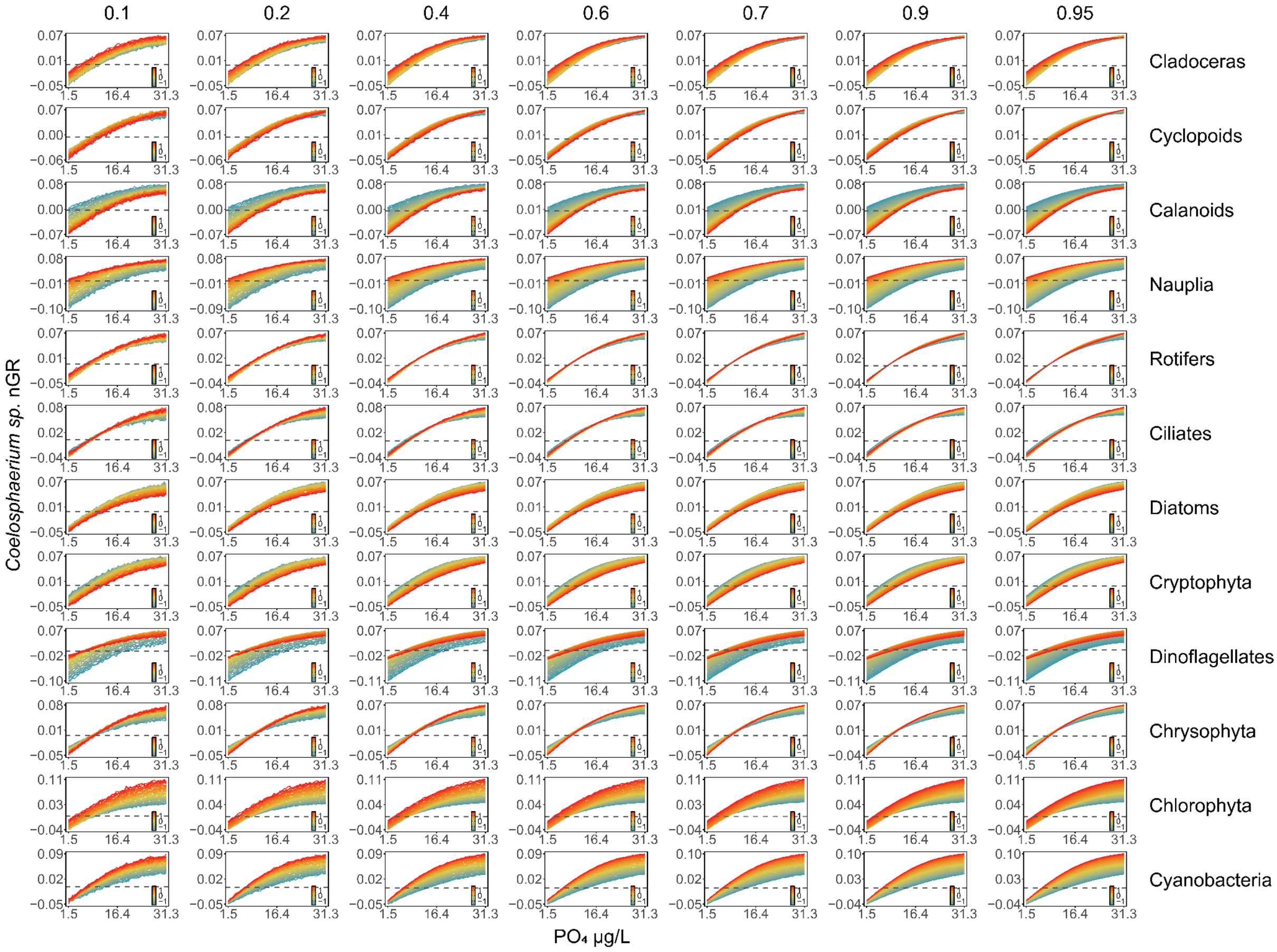

Fig. S41: Sensitivity analysis of *Coelosphaerium sp.* net growth rate to phosphate across different dataset sizes. Response functions of *Coelosphaerium* net growth rate (nGR) (Ln(ind/mL) · day^{-1}) to phosphate (PO_4^{3-}, µg/L) across different biotic interactions (rows) and data subset sizes (columns). Each panel represents a biotic interaction, with phosphate on the x-axis and changes in nGR on the y-axis. Columns correspond to different proportions of the dataset used (10%–95%) to assess response function robustness. Lines show mean estimated responses from n=50 subsamples (see Materials and Methods) and colour indicates the scaled density of the biotic factor in Ln(ind/mL). Patterns remain consistent across data lengths, confirming robustness.

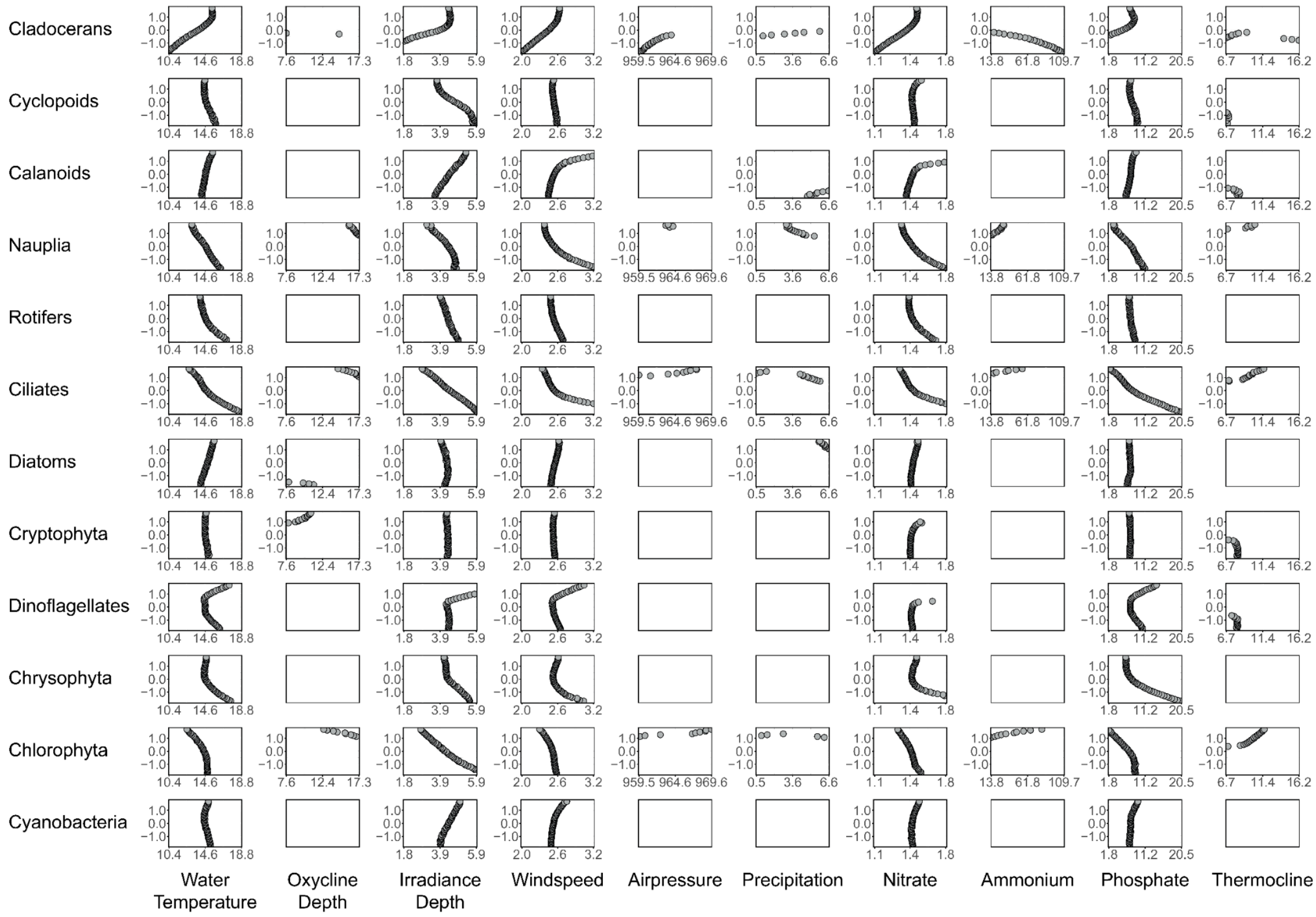

Fig. S42: Zero net growth analysis for *Chroococcus spp.* bloom formation across biotic-abiotic variable combinations. This figure analyses bloom initiation conditions by identifying zero-net growth isoclines from the response functions in **Fig. S12**. Each panel shows how the minimum environmental conditions required for blooming change with increasing densities of different planktonic groups. **Response patterns**: Positive correlations between blooming points and organism density indicate bloom inhibition (higher environmental thresholds needed); negative correlations indicate bloom facilitation (lower thresholds needed). **Key influences**: Cladocerans and ciliates strongly affect blooming conditions across most abiotic scenarios. **Notable absences**: White panels indicate conditions where blooming was never observed due to consistently negative or positive growth rates. **Units** as in **Fig. S10**.

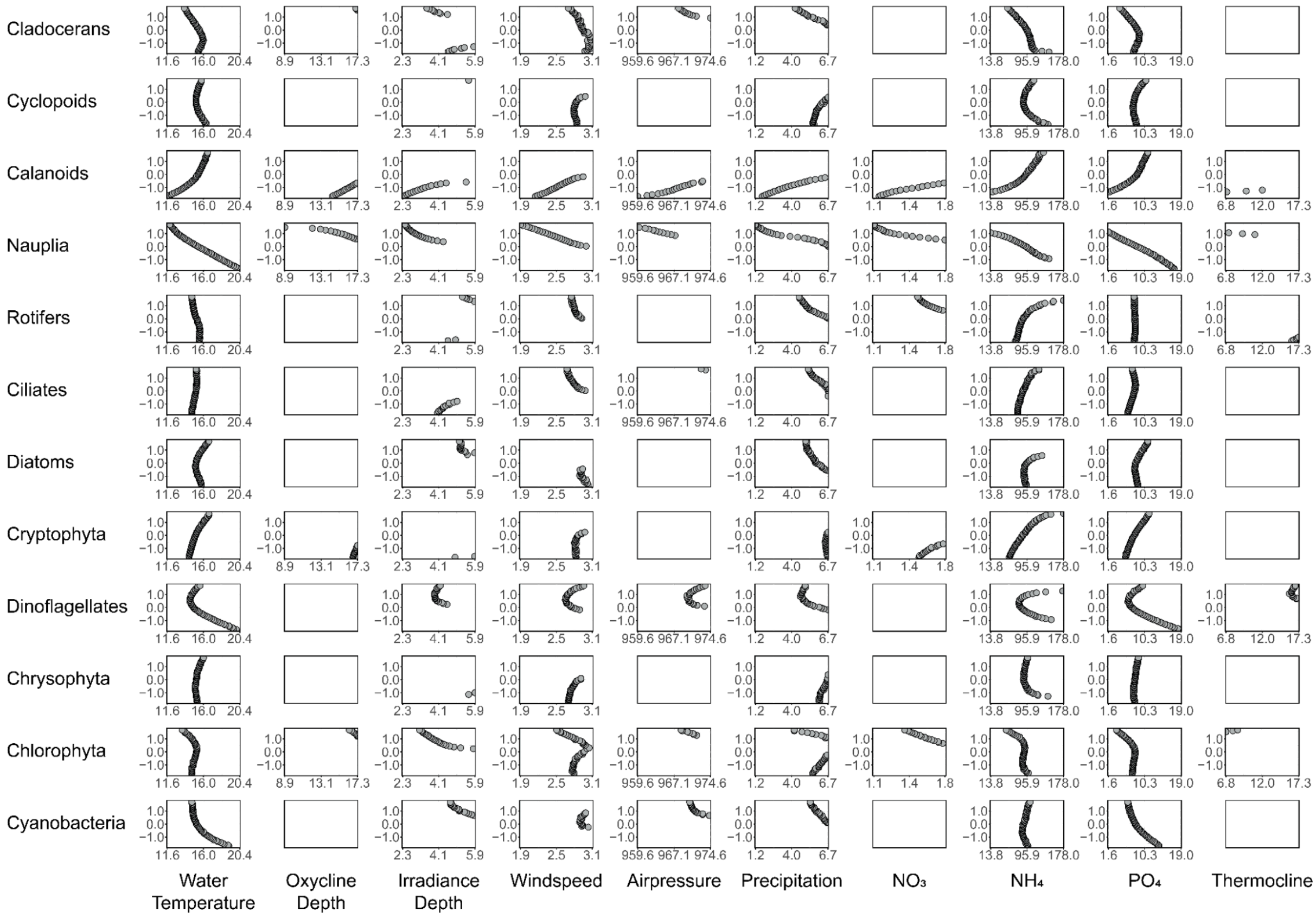

Fig. S43: Zero net growth analysis for *Coelosphaerium sp.* bloom formation across biotic-abiotic variable combinations. This figure analyses bloom initiation conditions by identifying zero-net growth isoclines from the response functions in **Fig. S13**. Each panel shows how the minimum environmental conditions required for blooming change with increasing densities of different planktonic groups. **Response patterns**: Positive correlations between blooming points and organism density indicate bloom inhibition (higher environmental thresholds needed); negative correlations indicate bloom facilitation (lower thresholds needed). **Key influences**: Calanoid copepods and their nauplii strongly affect blooming conditions across most abiotic scenarios. **Notable absences**: White panels indicate conditions where blooming was never observed due to consistently negative or positive growth rates. **Units** as in **Fig. S10**.

Table S1: Classified taxa present in zooplankton groups. Shown are the zooplankton groups used in the analysis, the corresponding classifier classes from the automated deep learning classifiers, and their underlying taxonomic composition.

**Zooplankton Group**	**Classifier Class**	**Taxonomic Composition**
Cladocerans	bosmina	*Bosmina sp.*
Cladocerans	daphnia	*Daphnia sp.*
Cladocerans	diaphanosoma	*Diaphanosoma sp.*
Cladocerans	daphnia_cucullata	*Daphnia cucullata*
Cladocerans	chydorus	*Chydorus sp.*
Cyclopoid copepods	cyclops	*Cyclops sp.*
Calanoid copepods	eudiaptomus	*Eudiaptomus sp.*
Nauplia	nauplius	Nauplius
Rotifers	ascomorpha	*Ascomorpha sp.*
Rotifers	asplanchna	*Asplanchna sp.*
Rotifers	collotheca	*Collotheca sp.*
Rotifers	conochilus	*Conochilus sp.*
Rotifers	kellicottia	*Kellicottia sp.*
Rotifers	keratella_cochlearis	*Keratella cochlearis*
Rotifers	keratella_quadrata	*Keratella quadrata*
Rotifers	polyarthra	*Polyarthra sp.*
Rotifers	rotifer	Rotifera
Rotifers	rotifer_long	Rotifera
Rotifers	rotifer_z	Rotifera
Rotifers	synchaeta	*Synchaeta sp.*
Ciliates	askenasia	*Askenasia sp.*
Ciliates	ciliate	Ciliophora
Ciliates	ciliate_big	Ciliophora
Ciliates	ciliate_blue	Ciliophora
Ciliates	ciliate_green	Ciliophora
Ciliates	ciliate_round	Ciliophora

Ciliates	coleps	*Coleps sp.*
Ciliates	didinium	*Didinium sp.*
Ciliates	paradileptus	*Paradileptus sp.*
Ciliates	protist_like_ciliate	Ciliophora
Ciliates	strombidium	*Strombidium sp.*
Ciliates	tintinnid	Tintinnida
Ciliates	vorticella_epistylis_like	*Vorticella sp.*

Table S2: Classified taxa present in phytoplankton groups. Shown are the phytoplankton groups used in the analysis, the corresponding classifier classes from the automated deep learning classifiers, and their underlying taxonomic composition (multiple species within a genus are denoted as "spp."

**Phytoplankton Group**	**Classifier Class**	**Taxonomic Composition**
Chrysophytes	dinobryon	*Dinobryon sp.*
Chrysophytes	dinobryon_single_cell	*Dinobryon sp.*
Chrysophytes	mallomonas_akrokomos	*Mallomonas akrokomos*
Chrysophytes	mallomonas_big	*Mallomonas sp.*
Chrysophytes	synura	*Synura sp.*
Chrysophytes	uroglena	*Uroglena sp.*
Cryptophytes	cryptomonas_cryptophyceae	*Cryptomonas sp.*
Cryptophytes	rhodomonas	*Rhodomonas sp.*
Chlorophytes	chlorophyte	*Chlorella sp.*<br>*Chlamydomonas sp*.<br>other Chlorophyceae
Chlorophytes	chlorophyte_colonial_dividing	*Coelastrum microporum*<br>other Chlorophyceae.
Chlorophytes	chlorophyte_elongated	*Ankyra sp.*<br>*Elakatothrix sp.*<br>*Bitrichia sp.*<br>other Chlorophyceae
Chlorophytes	chlorophyte_frame	Chlorophyceae
Chlorophytes	chlorophyte_square	*Crucigenia sp.*<br>*Willea sp.*<br>*Raysiella sp.,*
Chlorophytes	closterium	*Closterium sp.*
Chlorophytes	coelastrum_reticulatum	*Coelastrum reticulatum*
Chlorophytes	cosmarium	*Cosmarium sp.*
Chlorophytes	gonium	*Gonium sp.*
Chlorophytes	hormidium_like	*Hormidium sp.*
Chlorophytes	oocystaceae	*Oocystis spp..*<br>*Lagerheimia sp.*
Chlorophytes	oocystaceae_probably	*Oocystis spp..*<br>*Lagerheimia sp..*

Chlorophytes	pandorina	*Pandorina sp.*<br>*Eudorina sp.*
Chlorophytes	pediastrum	*Pediastrum spp.*
Chlorophytes	phacotus	*Phacotus sp.*
Chlorophytes	planktosphaeria	*Planktosphaeria sp.*
Chlorophytes	scenedesmus	*Scenedesmus sp.*
Chlorophytes	staurastrum	*Staurastrum sp.*
Chlorophytes	tetraedron	*Tetraedron sp.*
Diatoms	asterionella	*Asterionella sp.*
Diatoms	diatom_chain	*Tabellaria sp.*<br>*Diatoma sp.*
Diatoms	fragilaria	*Fragilaria crotonensis*<br>*Fragilaria capucina*
Diatoms	aulacoseira	*Aulacoseira sp.*
Diatoms	centric_diatom	Bacillariophyceae
Diatoms	pennate_diatom	Bacillariophyceae
Diatoms	synedra	*Synedra spp.*
Diatoms	synedra_angustissima	*Synedra angustissima*
Cyanobacteria	aphanizomenon	*Aphanizomenon flos-aquae.*
Cyanobacteria	chroococcales	*Chroococcus spp.*
Cyanobacteria	coelosphaerium	*Coelosphaerium sp.*
Cyanobacteria	cyanobacteria_colonial_blue	*Aphanothece spp.*<br>*Aphanocapsa spp.*<br>*Microcystis spp.*
Cyanobacteria	cyanobacteria_colonial_clathrate	*Aphanothece spp.*<br>*Aphanocapsa spp.*<br>*Microcystis spp.*
Cyanobacteria	cyanobacteria_colonial_probably	*Microcystis spp.*<br>*Coelosphaerium sp.*
Cyanobacteria	cyanobacteria_filamentous	*Aphanizomenon sp.*<br>*Dolichospermum spp.*<br>*Planktothrix sp.*
Cyanobacteria	dolichospermum	*Dolichospermum spp.*

Cyanobacteria	limnoraphis	*Limnoraphis spp.*
Cyanobacteria	snowella	*Snowella sp.*
Dinoflagellates	ceratium	*Ceratium sp.*
Dinoflagellates	gymnodinium	*Gimnodinium spp.*
Dinoflagellates	peridinium	*Peridinium aciculiferum*<br>*Peridinium willei*
Dinoflagellates	dinoflagellate_diamond	*Peridinium aciculiferum*<br>*Gymnodinium helveticum*<br>Dinophyceae
Toxic *Microcystaceae*	cyanobacteria_colonial_blue	*Aphanothece spp.*<br>*Aphanocapsa spp.*<br>*Microcystis spp.*
Toxic *Microcystaceae*	cyanobacteria_colonial_clathrate	*Aphanothece spp.*<br>*Aphanocapsa spp.*<br>*Microcystis spp.*
Dolichospermum spp.	dolichospermum	*Dolichospermum spp.*
Chroococcus spp.	chroococcales	*Chroococcus spp.*
Coelospharerium sp.	coelosphaerium	*Coelosphaerium sp.*

Table S3: The performance of classifiers from the hierarchical image classification.

	Classifier		
	Cyanobacteria	Phytoplankton	Zooplankton
**False Positives**	0.002	0.214	0.038
**Accuracy**	0.986	0.907	0.946
**F1 Score**	0.882	0.863	0.934
**AUC Macro**	0.998	0.614	0.998
**Precision**	0.92	0.876	0.932
**Recall**	0.856	0.842	0.938
