# Supplementary material for: Biotic interactions shape the realized niche of toxic cyanobacteria
Source: Nat Ecol Evol. 2026 Jul 20;10(8):1434–48. doi: 10.1038/s41559-026-03131-0 (PMC13441909; doi:10.1038/s41559-026-03131-0)
Supplement: Supplementary file 2 [file 41559_2026_3131_MOESM2_ESM.pdf]

nature portfolio

Corresponding author(s): Pinelopi Ntetsika
Last updated by author(s): May 26, 2026

Reporting Summary

Nature Portfolio wishes to improve the reproducibility of the work that we publish. This form provides structure for consistency and transparency in reporting. For further information on Nature Portfolio policies, see our Editorial Policies and the Editorial Policy Checklist.

Statistics

For all statistical analyses, confirm that the following items are present in the figure legend, table legend, main text, or Methods section.

n/a	Confirmed	
☐	☒	The exact sample size (n) for each experimental group/condition, given as a discrete number and unit of measurement
☒	☐	A statement on whether measurements were taken from distinct samples or whether the same sample was measured repeatedly
☒	☐	The statistical test(s) used AND whether they are one- or two-sided<br>*Only common tests should be described solely by name; describe more complex techniques in the Methods section.*
☐	☒	A description of all covariates tested
☒	☐	A description of any assumptions or corrections, such as tests of normality and adjustment for multiple comparisons
☐	☒	A full description of the statistical parameters including central tendency (e.g. means) or other basic estimates (e.g. regression coefficient) AND variation (e.g. standard deviation) or associated estimates of uncertainty (e.g. confidence intervals)
☒	☐	For null hypothesis testing, the test statistic (e.g. F, t, r) with confidence intervals, effect sizes, degrees of freedom and P value noted<br>*Give P values as exact values whenever suitable.*
☒	☐	For Bayesian analysis, information on the choice of priors and Markov chain Monte Carlo settings
☒	☐	For hierarchical and complex designs, identification of the appropriate level for tests and full reporting of outcomes
☐	☒	Estimates of effect sizes (e.g. Cohen's d, Pearson's r), indicating how they were calculated

Our web collection on statistics for biologists contains articles on many of the points above.

Software and code

Policy information about availability of computer code

Data collection	The software used for data collection is available at https://aquascope.ch/links/.
Data analysis	The R scripts used to generate the results of this manuscript are made available through the Eawag Research Data Institutional Repository (ERIC/Open) at https://doi.org/10.25678/000GPG, together with the final aggregated dataset. All analyses have been performed in R 4.5.1 with the use of the packages tidyverse (2.0.0), rEDM (0.4.7 and 1.15.4), vegan (2.7.2) and glmnet (4.1.10). All figures have been generated with the use of the package ggplot2 (3.5.2).

For manuscripts utilizing custom algorithms or software that are central to the research but not yet described in published literature, software must be made available to editors and reviewers. We strongly encourage code deposition in a community repository (e.g. GitHub). See the Nature Portfolio guidelines for submitting code & software for further information.

Data

Policy information about availability of data

All manuscripts must include a data availability statement. This statement should provide the following information, where applicable:

- Accession codes, unique identifiers, or web links for publicly available datasets
- A description of any restrictions on data availability
- For clinical datasets or third party data, please ensure that the statement adheres to our policy

The raw data supporting this study's findings are openly available from the Eawag Research Data Institutional Repository (ERIC/Open) at https://

doi.org/10.25678/000GPG.

Research involving human participants, their data, or biological material

Policy information about studies with human participants or human data. See also policy information about sex, gender (identity/presentation), and sexual orientation and race, ethnicity and racism.

Reporting on sex and gender	*Use the terms sex (biological attribute) and gender (shaped by social and cultural circumstances) carefully in order to avoid confusing both terms. Indicate if findings apply to only one sex or gender; describe whether sex and gender were considered in study design; whether sex and/or gender was determined based on self-reporting or assigned and methods used. Provide in the source data disaggregated sex and gender data, where this information has been collected, and if consent has been obtained for sharing of individual-level data; provide overall numbers in this Reporting Summary. Please state if this information has not been collected. Report sex- and gender-based analyses where performed, justify reasons for lack of sex- and gender-based analysis.*
Reporting on race, ethnicity, or other socially relevant groupings	*Please specify the socially constructed or socially relevant categorization variable(s) used in your manuscript and explain why they were used. Please note that such variables should not be used as proxies for other socially constructed/relevant variables (for example, race or ethnicity should not be used as a proxy for socioeconomic status). Provide clear definitions of the relevant terms used, how they were provided (by the participants/respondents, the researchers, or third parties), and the method(s) used to classify people into the different categories (e.g. self-report, census or administrative data, social media data, etc.) Please provide details about how you controlled for confounding variables in your analyses.*
Population characteristics	*Describe the covariate-relevant population characteristics of the human research participants (e.g. age, genotypic information, past and current diagnosis and treatment categories). If you filled out the behavioural & social sciences study design questions and have nothing to add here, write "See above."*
Recruitment	*Describe how participants were recruited. Outline any potential self-selection bias or other biases that may be present and how these are likely to impact results.*
Ethics oversight	*Identify the organization(s) that approved the study protocol.*

Note that full information on the approval of the study protocol must also be provided in the manuscript.

Field-specific reporting

Please select the one below that is the best fit for your research. If you are not sure, read the appropriate sections before making your selection.

☐ Life sciences ☐ Behavioural & social sciences ☒ Ecological, evolutionary & environmental sciences

For a reference copy of the document with all sections, see nature.com/documents/nr-reporting-summary-flat.pdf

Ecological, evolutionary & environmental sciences study design

All studies must disclose on these points even when the disclosure is negative.

Study description	This is an observational, data-driven, ecological modelling study. It relies on high-frequency, in situ observational data, machine learning, and empirical dynamic modelling. The focus is on inferring causal interactions and predicting ecological dynamics—particularly bloom thresholds—under varying biotic and abiotic conditions. We investigated the drivers of cyanobacterial growth in 4 target taxa (Microcystis group, Dolichospermum sp, Chroococcus sp., Coelosphaerium sp) in Lake Greifensee using 10 environmental factors (phosphate, nitrate, ammonium, temperature, oxycline depth, air pressure, precipitation, windspeed, irradiance depth, thermocline depth) and the densities of 12 biotic factors (cyanobacteria, calanoid copepods, cyclopoid copepods, cladocerans, rotifers, ciliates, nauplia, dinoflagellates, diatoms, cryptophyta, chrysophyta, chlorophyta, density of target cyanobacteria) as explanatory variables.
Research sample	The research sample consists of daily observations of aquatic plankton communities and environmental variables collected from Lake Greifensee, a eutrophic peri-alpine lake in Switzerland, spanning March 2019 to December 2023. This sample includes high-resolution time series (1742 data points) of cyanobacterial taxa (Microcystis group, Dolichospermum sp, Chroococcus sp., Coelosphaerium sp), other phytoplankton (dinoflagellates, diatoms, cryptophyta, chrysophyta, chlorophyta,) and zooplankton groups (calanoid copepods, cyclopoid copepods, cladocerans, rotifers, ciliates, nauplia), and key abiotic factors phosphate, nitrate, ammonium, temperature, oxycline depth, air pressure, precipitation, windspeed, irradiance depth, thermocline depth) influencing lake ecology. The biotic samples are daily averages of hourly sample ensuring representative estimates of organismal densities across all tracked taxa.
Sampling strategy	All data used in this study were obtained from the long-term automated monitoring platform in Lake Greifensee, which is equipped with a CTD probe, a meteorological station, and a dual-magnification dark-field underwater microscope (DSPC) for high-frequency monitoring of the plankton community. Meteorological data, including air pressure, precipitation, and wind speed, were recorded at high temporal resolution (1–30 minute intervals) at the station. The CTD probe collects physical parameters of the water column—including temperature, oxygen saturation, and photosynthetically active radiation (PAR)—at 0.1-meter vertical resolution from 1 to 17 meters depth, with measurements taken every 3 to 6 hours. For consistency and ecological relevance, we used data from the

	upper 0–8 meters, corresponding to the photic zone above the thermocline where plankton are most abundant and image acquisition occurs. The DSPC, positioned at 3 meters depth within the photic zone, captures images of the plankton community every hour for a duration of 10 minutes. This imaging protocol provides high-frequency biotic data encompassing the majority of the freshwater plankton food web. For analyses of plankton group densities, we included only dates with a minimum of 19 hourly sampling events, thereby ensuring that our daily averages are robust and representative of diel variability. Nutrient concentrations were obtained through manual water sampling at 3 meters depth from the monitoring station on a weekly basis (or biweekly during winter months) and interpolated at a daily frequency using random forest models trained on hourly CTD and meteorological measurements at the lake. All data were averaged at a daily scale for analysis. For more information see Eyring, Stefanie, Marta Reyes, Ewa Merz, Marco Baity-Jesi, Pinelopi Ntesika, Christian Ebi, Stuart Dennis, and Francesco Pomati. 2025. "Five Years of High-Frequency Data of Phytoplankton Zooplankton and Limnology from a Temperate Eutrophic Lake." bioRxiv. https://doi.org/10.1101/2025.03.20.644357.
Data collection	Data collection is automated for all variables used in this study apart from the nutrient concentrations which were estimated from sampled lake water from our monitoring platform. Stefanie Eyering was the responsible for abiotic data management, Stuart Dennis and Marco Baity-Jesi are responsible for the collection and processing of the plankton images. For more information see Eyring, Stefanie, Marta Reyes, Ewa Merz, Marco Baity-Jesi, Pinelopi Ntesika, Christian Ebi, Stuart Dennis, and Francesco Pomati. 2025. "Five Years of High-Frequency Data of Phytoplankton Zooplankton and Limnology from a Temperate Eutrophic Lake." bioRxiv. https://doi.org/10.1101/2025.03.20.644357.
Timing and spatial scale	Biotic data were collected hourly from 26 March 2019 to 31 December 2023 and averaged to daily estimates. We excluded the following sampling dates due to maintainance of instruments or unexpected malfunctioning or when none adequate sampling imaging events were occurring (<20 per day). The following large periods were excluded from the data with a few other occasional removed data: 2019/07/30-2019/08/13, 2020/01/07-2020/01/21, 2020/07/21-2020/07/23, 2022/11/25-2022/11/28, 2022/12/10-2022/12/12, 2023/04/01-2023/04/03. All data are collected at the monitoring station at Lake Greifensee: 47° 21' 59" N, 8° 39' 54.4" E. For more information see Eyring, Stefanie, Marta Reyes, Ewa Merz, Marco Baity-Jesi, Pinelopi Ntesika, Christian Ebi, Stuart Dennis, and Francesco Pomati. 2025. "Five Years of High-Frequency Data of Phytoplankton Zooplankton and Limnology from a Temperate Eutrophic Lake." bioRxiv. https://doi.org/10.1101/2025.03.20.644357.
Data exclusions	To reduce the influence of extreme outliers and improve the interpretability of the multivariate partial response functions, we excluded the upper and lower 5% of values (i.e., the extreme 10%) for each explanatory variable prior to prediction only on this part of the analysis. This trimming helps ensure that the response functions reflect the central tendency of the relationships rather than being skewed by rare or extreme observations, which may arise from sensor noise, rare events, or transient anomalies not representative of the typical system behavior.
Reproducibility	Not applicable.
Randomization	Not applicable.
Blinding	Not applicable.

Did the study involve field work? ☒ Yes ☐ No

Field work, collection and transport

Field conditions	We collected data in the middle of Lake Greifensee - see above. We monitored all water and meteo parameters at the monitoring station.
Location	The site is at coordinates 45°57033.4300N, 8°52053.4900E, at 436 m above sea level
Access & import/export	N/A
Disturbance	no disturbance

Reporting for specific materials, systems and methods

We require information from authors about some types of materials, experimental systems and methods used in many studies. Here, indicate whether each material, system or method listed is relevant to your study. If you are not sure if a list item applies to your research, read the appropriate section before selecting a response.

Materials & experimental systems

n/a	Involved in the study
☒	☐ Antibodies
☒	☐ Eukaryotic cell lines
☒	☐ Palaeontology and archaeology
☒	☐ Animals and other organisms
☒	☐ Clinical data
☒	☐ Dual use research of concern
☒	☐ Plants

Methods

n/a	Involved in the study
☒	☐ ChIP-seq
☒	☐ Flow cytometry
☒	☐ MRI-based neuroimaging

Plants

Seed stocks

Report on the source of all seed stocks or other plant material used. If applicable, state the seed stock centre and catalogue number. If plant specimens were collected from the field, describe the collection location, date and sampling procedures.

Novel plant genotypes

Describe the methods by which all novel plant genotypes were produced. This includes those generated by transgenic approaches, gene editing, chemical/radiation-based mutagenesis and hybridization. For transgenic lines, describe the transformation method, the number of independent lines analyzed and the generation upon which experiments were performed. For gene-edited lines, describe the editor used, the endogenous sequence targeted for editing, the targeting guide RNA sequence (if applicable) and how the editor was applied.

Authentication

Describe any authentication procedures for each seed stock used or novel genotype generated. Describe any experiments used to assess the effect of a mutation and, where applicable, how potential secondary effects (e.g. second site T-DNA insertions, mosiacism, off-target gene editing) were examined.
