# Supplementary material for: Biotic interactions shape the realized niche of toxic cyanobacteria
Source: Nat Ecol Evol. 2026 Jul 20;10(8):1434–48. doi: 10.1038/s41559-026-03131-0 (PMC13441909; doi:10.1038/s41559-026-03131-0)
Supplement: Supplementary file 3 [file 41559_2026_3131_MOESM3_ESM.pdf]

nature portfolio

# Peer Review File

# Biotic interactions shape the realised niche of toxic cyanobacteria

Corresponding Author: Ms Pinelopi Ntetsika

Parts of this Peer Review File have been redacted as indicated to remove third-party material.

Version 0:

Reviewer comments:

Reviewer #3

(Remarks to the Author)
The authors greatly improved their manuscript, and they clarified many of the points raised. Overall, I still think this is great work, as it is based on high frequency plankton data and provides a unique opportunity to generate understanding on plankton dynamics. It strongly hinges on data analyses, which has been done very thorough. While I am not an expert, there are a few choices and their consequences that remain elusive. Overall, the authors seem to oversell their work by emphasizing toxic cyanobacteria, and in relation to this, some conclusions seem to stretch it. This is a pity, as the work does not need this. It is strong in providing insights in complex plankton dynamics in natural systems, whatever the chosen phytoplankton group. Indeed, by focusing on net growth rates, it is the dynamics that is of interest, while risks of cyanobacterial blooms are not related to their growth, but rather population densities as those more generally link to toxins (and hence the risk). Below I listed some of my remaining concerns:
1) In answer to my question why cyanobacterial net growth rates were compared to biomass of other plankton groups, the authors refer to the classical Lotka-Volterra concept. They state that growth rate (as a process) depends on densities (as state variables). This is where to me the terminology gets confusing. While I see the intrinsic growth rate as a process, the per capita growth rate is not (as it is the net change in population density following from gains and losses). To me, it seems that inferring the intrinsic growth rate of cyanobacteria would involve relating the per capita change of cyanobacteria with their abundance. From the used calculations, it seems the authors calculate the intrinsic growth rate directly, and then use this as analogue to per capita change for comparison with the other population densities (used as state variables). This needs further clarification, and perhaps some indication on the consequence of this. I understand the method has been used for an earlier study (Nguyen et al. 2025, PNAS), but that study is constrained to phytoplankton groups, compares the intrinsic growth rates across all involved groups, and includes some clear constraints of the approach.
2) I understand how facilitation is concluded, but still do not see how this can be differentiated from co-occurrence, and/or inversed directionality where cyanobacteria facilitate other taxa? While I understand that the focus of the current study is not about the role of cyanobacteria on other plankton taxa, the authors do infer the direction from their findings. This needs to be more clearly emphasized.
3) The risk of cyanobacterial blooms is mainly linked to their biomass, as that often is well correlated with the toxin concentrations (which is the reason why they form a risk). This is not the same as positive growth rates, and one may even argue that at the onset of a bloom, growth may be high but the risk is still low. While at the peak of a bloom, the growth is low, but the risk is high. Specific outcomes, such as thresholds (L371) or monitoring (L395-401), seem too farfetched. There would be sufficient novelty when focusing on plankton dynamics, and I would strongly suggest to tone down those parts. Rather, I would like to see some words on how the current approach would look like when targeting other groups (at least in discussion) in relation to cyanobacteria, especially given that other groups are often more dominant in lake Greifensee; as more clearly shown in Nguyen et al. 2025).
4) The term ‘Toxic cyanobacteria’ in the title and ‘Toxic Microcystaceae’ is misleading, as it would suggest the authors actually assessed toxins. Why not focus on Microcystis and Dolichospermum, and, as currently already done, show the other genera in the suppl.

Reviewer #4

(Remarks to the Author)
I have reviewed the manuscript titled "Biotic interactions shape the realised niche of toxic cyanobacteria" by Ntetsika et al.,

submitted to Nature Ecology & Evolution. The authors analyzed unique ecological time series from Lake Greifensee in Switzerland to understand how the net growth rate (nGR) of potentially harmful cyanobacterial groups is affected by biotic and abiotic factors. The authors utilized specific time series analytical tools, called empirical dynamic modeling (EDM), to address the nonlinearity and high dimensionality of ecological time series. They found that the thresholds of physical variables (e.g., temperature and nutrients) enabling nGR > 0 (defined by the authors as the "realised niche") varied depending on the abundance of co-occurring plankton taxa, suggesting that biotic interactions can modify the response of cyanobacterial groups to physical parameters. My expertise lies in general community ecology and statistical ecology, and I have limited knowledge of the ecology and biology of specific freshwater plankton taxa. Therefore, my comments mostly on the logical flow and statistical analyses.

Overall, I am quite positive about this manuscript. Although the idea that the abundance of cyanobacteria is determined by both abiotic and biotic factors is not new (as also pointed out by the original Referee #2), the "quantitative" evaluation of how biotic factors alter the physical thresholds is novel (as far as I know). This is particularly challenging under field conditions, and the authors achieved this by utilizing recently developed nonlinear time series analysis methods. Their findings are ecologically meaningful and can contribute to more accurate predictions of harmful algal blooms.

I have also read the comments from the original referees and the authors' responses. I agree with most of the original referees' comments and believe that careful wording is necessary to communicate the ideas accurately. The authors have revised terms such as "response" and "fundamental" and have clearly defined the concept of "realised niche." Based on my review of the revised manuscript, the concept is now clear.

Although I am generally positive and understand that the authors have already done a substantial amount of work, however, I still have several concerns about the data analysis. I believe these concerns should be addressed before the manuscript is accepted.

1. The authors smoothed the plankton densities using local polynomial regression to remove random noise from the original time series (L477 in the methods section). While smoothing is useful for capturing the general trend in time series, I wonder whether it is appropriate to apply it to a chaotic time series (as the authors stated in their response letter that they are "chaotic"). If the dynamics are governed by linear processes with some random errors, smoothing can effectively remove these errors. However, if the dynamics are governed by a nonlinear deterministic process, smoothing may eliminate ecologically meaningful information from the time series. The authors can test whether smoothing influences the outcome of this analysis using at least two approaches: (i) analyzing the raw data and comparing the results to those from the smoothed time series, and (ii) generating artificial time series (for example, from simple differential equations, such as those used in Sugihara et al. 2012 Science), applying a smoothing function, and conducting EDM. The second approach is particularly effective for assessing the appropriateness of the smoothing method. I believe the authors should demonstrate that smoothing does not affect the final conclusions of this manuscript.

2. Most of the physical parameters were predicted using a Random Forest (RF) approach. While I totally understand that daily measurements of these parameters are currently impractical, I am concerned that the choice of prediction method may affect the results. Is it possible to show how the choice of prediction methods influences the outcome of EDM? One suggestion for the authors is to use only the real measurement values (i.e., weekly data), conduct EDM, and compare it to the results of the daily time series analysis. Although analyzing weekly data may change the interpretation of the results (as the time scale of the dynamics changes from daily to weekly), this type of validation could demonstrate the robustness of the analysis. Also, the relevant topic of interpolating missing values was studied in Carpenter et al. (2025) in "Repairing Gaps in Ecological Time Series" (https://doi.org/10.1111/2041-210X.14491; just for your information).

3. Technical Details of CCM. In the original CCM paper (Sugihara et al. 2012), two important criteria are used to assess causality. The first is rho (forecasting skill) at the largest library size. The second is "convergence," which measures the difference between rho at the largest library size and that at the smallest library size. This convergence checks whether the reconstructed trajectories ultimately reach a 1:1 mapping and helps determine if the observed cross-map skill is due to pseudo-correlation or not. The authors did not assess this "convergence" in the manuscript. I believe that the surrogate test of rho at the largest library size alone is insufficient to exclude the possibility of pseudo-correlation, and they should also check for convergence. Alternatively, the authors could utilize a more recently developed method called "unified-information theoretic causality" (https://doi.org/10.1101/2023.04.20.537743), which integrates transfer entropy and CCM and does not require testing for convergence.

4. The authors carefully discussed the limitations of their study in response to the comments from the original referees. While they have addressed several important topics, they may also comment on the inclusion of physical processes such as water circulation and waves. As Referee #3 pointed out, the study is based on a single location, but monitoring multiple locations and utilizing spatiotemporal information would definitely improve prediction accuracy, especially in more open and dynamic marine systems where harmful algal blooms have also been observed. The authors briefly acknowledged this in L364, but they can explicitly state that integrating physical processes or spatial information is a promising direction for future research. Although EDM is a novel time series analysis approach, ecological statistics should ultimately be spatiotemporal. The integration of spatial information in EDM (spatial EDM) is an exciting direction for future research (e.g., https://doi.org/10.1111/2041-210X.13511).

Minor points

- Abstract: It is better to mention that the authors conducted "causal analysis," not "correlation analysis," based on nonlinear

time series analysis.
- Line 95: The authors may clarify that they performed time-series-based causal detection analysis and used "predicted" daily physical parameters here.
- Line 123: It is better to say " the natural logarithm" is taken instead of "Ln-transformed" for clarity.
- Line 134: The authors may introduce the term "empirical dynamic modeling (EDM)" here.
- Line 205: Figure 2 indicates that the benchmark model (persistence model) outperformed the MDR S-map models when the forecast horizon is from 0 to about 5 days (or sometimes more). Does this suggest that the MDR S-map did not provide meaningful predictions for the 0-5 day period? Also, is this related to the smoothing of the original time series (as the smoothing may strengthen the autocorrelation)? What would happen if EDM were applied to the original time series?
- Line 357: The term "empirical dynamic modeling" should be introduced earlier.
- Line 360: The term "embedding dimension" was not explained.
- Line 517: Why was the embedding dimension fixed at 15? It should be determined for each variable.
- Line 531: The term "EDM" was not defined before.
- Line 532: CCM was conducted using TP=0, while the MDR S-map was conducted using TP=1. Why didn't the authors use TP=-1 for CCM?
- Figure 1A: It should be clarified on the y-axis that these are also "Ln-transformed" values.

Decision Letter:

24th November 2025

*Please ensure you delete the link to your author homepage in this e-mail if you wish to forward it to your co-authors.

Dear Ms Ntetsika,

Your manuscript entitled "Biotic interactions shape the realised niche of toxic cyanobacteria" has now been seen by 2 reviewers, whose comments are below. One of the reviewers (reviewer #3) is the same from the previous round of review and there are comments from a new reviewer (reivewer #4). The reviewers have raised a number of concerns which will need to be addressed before we can offer publication in Nature Ecology & Evolution. We will therefore need to see your responses to the criticisms raised and to some editorial concerns, along with a revised manuscript, before we can reach a final decision regarding publication.

In addition to addressing the reviewers' remaining concerns, we would like you to try increase clarity in the manuscript when describing physiological vs ecological processes. For example, when discussing net growth rate rather than specific growth rate, and for discussing growth thresholds that might be confused with physiological thresholds.

We therefore invite you to revise your manuscript taking into account all reviewer and editor comments. Please highlight all changes in the manuscript text file in Microsoft Word format.

We are committed to providing a fair and constructive peer-review process. Do not hesitate to contact us if there are specific requests from the reviewers that you believe are technically impossible or unlikely to yield a meaningful outcome.

When revising your manuscript:

* Include a “Response to reviewers” document detailing, point-by-point, how you addressed each reviewer comment. If no action was taken to address a point, you must provide a compelling argument. This response will be sent back to the reviewers along with the revised manuscript.

* If you have not done so already please begin to revise your manuscript so that it conforms to our Article format instructions at http://www.nature.com/natecolevol/info/final-submission. Refer also to any guidelines provided in this letter.

* Include a revised version of any required reporting checklist. It will be available to referees (and, potentially, statisticians) to aid in their evaluation if the manuscript goes back for peer review. A revised checklist is essential for re-review of the paper.

* Extended Data Figures - please ensure that any supplementary figures and tables that are crucial to the manuscript’s conclusions are converted into Extended Data figures and tables to increase visibility of these data. Extended Data figures and tables are online-only (present in the online PDF and full-text HTML versions of the paper), peer-reviewed display items that provide essential background to the article but are not included in the main article due to space constraints. A maximum of ten Extended Data display items (figures and tables) is permitted.

Please use the link below to submit your revised manuscript and related files:

Link Redacted

**Note:** This URL links to your confidential home page and associated information about manuscripts you may have submitted, or that you are reviewing for us. If you wish to forward this email to co-authors, please delete the link to your homepage.

We hope to receive your revised manuscript within four to eight weeks. If you cannot send it within this time, please let us

know. We will be happy to consider your revision so long as nothing similar has been accepted for publication at Nature Ecology & Evolution or published elsewhere.

Nature Ecology & Evolution is committed to improving transparency in authorship. As part of our efforts in this direction, we are now requesting that all authors identified as 'corresponding author' on published papers create and link their Open Researcher and Contributor Identifier (ORCID) with their account on the Manuscript Tracking System (MTS), prior to acceptance. ORCID helps the scientific community achieve unambiguous attribution of all scholarly contributions. You can create and link your ORCID from the home page of the MTS by clicking on 'Modify my Springer Nature account'. For more information please visit please visit <a href="http://www.springernature.com/orcid">www.springernature.com/orcid</a>.

Please do not hesitate to contact me if you have any questions or would like to discuss these revisions further.

We look forward to seeing the revised manuscript and thank you for the opportunity to review your work.

[redacted]

Reviewer expertise:

Reviewer #3: phytoplankton ecology, harmful cyanobacteria

Reviewer #4: ecosystem dynamics, nonlinear time-series analysis, CCM

Reviewers' comments:

Reviewer #3 (Remarks to the Author):

The authors greatly improved their manuscript, and they clarified many of the points raised. Overall, I still think this is great work, as it is based on high frequency plankton data and provides a unique opportunity to generate understanding on plankton dynamics. It strongly hinges on data analyses, which has been done very thorough. While I am not an expert, there are a few choices and their consequences that remain elusive. Overall, the authors seem to oversell their work by emphasizing toxic cyanobacteria, and in relation to this, some conclusions seem to stretch it. This is a pity, as the work does not need this. It is strong in providing insights in complex plankton dynamics in natural systems, whatever the chosen phytoplankton group. Indeed, by focusing on net growth rates, it is the dynamics that is of interest, while risks of cyanobacterial blooms are not related to their growth, but rather population densities as those more generally link to toxins (and hence the risk). Below I listed some of my remaining concerns:
1) In answer to my question why cyanobacterial net growth rates were compared to biomass of other plankton groups, the authors refer to the classical Lotka-Volterra concept. They state that growth rate (as a process) depends on densities (as state variables). This is where to me the terminology gets confusing. While I see the intrinsic growth rate as a process, the per capita growth rate is not (as it is the net change in population density following from gains and losses). To me, it seems that inferring the intrinsic growth rate of cyanobacteria would involve relating the per capita change of cyanobacteria with their abundance. From the used calculations, it seems the authors calculate the intrinsic growth rate directly, and then use this as analogue to per capita change for comparison with the other population densities (used as state variables). This needs further clarification, and perhaps some indication on the consequence of this. I understand the method has been used for an earlier study (Nguyen et al. 2025, PNAS), but that study is constrained to phytoplankton groups, compares the intrinsic growth rates across all involved groups, and includes some clear constraints of the approach.
2) I understand how facilitation is concluded, but still do not see how this can be differentiated from co-occurrence, and/or inversed directionality where cyanobacteria facilitate other taxa? While I understand that the focus of the current study is not about the role of cyanobacteria on other plankton taxa, the authors do infer the direction from their findings. This needs to be more clearly emphasized.
3) The risk of cyanobacterial blooms is mainly linked to their biomass, as that often is well correlated with the toxin concentrations (which is the reason why they form a risk). This is not the same as positive growth rates, and one may even argue that at the onset of a bloom, growth may be high but the risk is still low. While at the peak of a bloom, the growth is low, but the risk is high. Specific outcomes, such as thresholds (L371) or monitoring (L395-401), seem too farfetched. There would be sufficient novelty when focusing on plankton dynamics, and I would strongly suggest to tone down those parts. Rather, I would like to see some words on how the current approach would look like when targeting other groups (at least in discussion) in relation to cyanobacteria, especially given that other groups are often more dominant in lake Greifensee; as more clearly shown in Nguyen et al. 2025).
4) The term 'Toxic cyanobacteria' in the title and 'Toxic Microcystaceae' is misleading, as it would suggest the authors actually assessed toxins. Why not focus on Microcystis and Dolichospermum, and, as currently already done, show the other genera in the suppl.

Reviewer #4 (Remarks to the Author):

I have reviewed the manuscript titled "Biotic interactions shape the realised niche of toxic cyanobacteria" by Ntetsika et al., submitted to Nature Ecology & Evolution. The authors analyzed unique ecological time series from Lake Greifensee in

Switzerland to understand how the net growth rate (nGR) of potentially harmful cyanobacterial groups is affected by biotic and abiotic factors. The authors utilized specific time series analytical tools, called empirical dynamic modeling (EDM), to address the nonlinearity and high dimensionality of ecological time series. They found that the thresholds of physical variables (e.g., temperature and nutrients) enabling nGR > 0 (defined by the authors as the "realised niche") varied depending on the abundance of co-occurring plankton taxa, suggesting that biotic interactions can modify the response of cyanobacterial groups to physical parameters. My expertise lies in general community ecology and statistical ecology, and I have limited knowledge of the ecology and biology of specific freshwater plankton taxa. Therefore, my comments mostly on the logical flow and statistical analyses.

Overall, I am quite positive about this manuscript. Although the idea that the abundance of cyanobacteria is determined by both abiotic and biotic factors is not new (as also pointed out by the original Referee #2), the "quantitative" evaluation of how biotic factors alter the physical thresholds is novel (as far as I know). This is particularly challenging under field conditions, and the authors achieved this by utilizing recently developed nonlinear time series analysis methods. Their findings are ecologically meaningful and can contribute to more accurate predictions of harmful algal blooms.

I have also read the comments from the original referees and the authors' responses. I agree with most of the original referees' comments and believe that careful wording is necessary to communicate the ideas accurately. The authors have revised terms such as "response" and "fundamental" and have clearly defined the concept of "realised niche." Based on my review of the revised manuscript, the concept is now clear.

Although I am generally positive and understand that the authors have already done a substantial amount of work, however, I still have several concerns about the data analysis. I believe these concerns should be addressed before the manuscript is accepted.

1. The authors smoothed the plankton densities using local polynomial regression to remove random noise from the original time series (L477 in the methods section). While smoothing is useful for capturing the general trend in time series, I wonder whether it is appropriate to apply it to a chaotic time series (as the authors stated in their response letter that they are "chaotic"). If the dynamics are governed by linear processes with some random errors, smoothing can effectively remove these errors. However, if the dynamics are governed by a nonlinear deterministic process, smoothing may eliminate ecologically meaningful information from the time series. The authors can test whether smoothing influences the outcome of this analysis using at least two approaches: (i) analyzing the raw data and comparing the results to those from the smoothed time series, and (ii) generating artificial time series (for example, from simple differential equations, such as those used in Sugihara et al. 2012 Science), applying a smoothing function, and conducting EDM. The second approach is particularly effective for assessing the appropriateness of the smoothing method. I believe the authors should demonstrate that smoothing does not affect the final conclusions of this manuscript.

2. Most of the physical parameters were predicted using a Random Forest (RF) approach. While I totally understand that daily measurements of these parameters are currently impractical, I am concerned that the choice of prediction method may affect the results. Is it possible to show how the choice of prediction methods influences the outcome of EDM? One suggestion for the authors is to use only the real measurement values (i.e., weekly data), conduct EDM, and compare it to the results of the daily time series analysis. Although analyzing weekly data may change the interpretation of the results (as the time scale of the dynamics changes from daily to weekly), this type of validation could demonstrate the robustness of the analysis. Also, the relevant topic of interpolating missing values was studied in Carpenter et al. (2025) in "Repairing Gaps in Ecological Time Series" (https://doi.org/10.1111/2041-210X.14491; just for your information).

3. Technical Details of CCM. In the original CCM paper (Sugihara et al. 2012), two important criteria are used to assess causality. The first is rho (forecasting skill) at the largest library size. The second is "convergence," which measures the difference between rho at the largest library size and that at the smallest library size. This convergence checks whether the reconstructed trajectories ultimately reach a 1:1 mapping and helps determine if the observed cross-map skill is due to pseudo-correlation or not. The authors did not assess this "convergence" in the manuscript. I believe that the surrogate test of rho at the largest library size alone is insufficient to exclude the possibility of pseudo-correlation, and they should also check for convergence. Alternatively, the authors could utilize a more recently developed method called "unified-information theoretic causality" (https://doi.org/10.1101/2023.04.20.537743), which integrates transfer entropy and CCM and does not require testing for convergence.

4. The authors carefully discussed the limitations of their study in response to the comments from the original referees. While they have addressed several important topics, they may also comment on the inclusion of physical processes such as water circulation and waves. As Referee #3 pointed out, the study is based on a single location, but monitoring multiple locations and utilizing spatiotemporal information would definitely improve prediction accuracy, especially in more open and dynamic marine systems where harmful algal blooms have also been observed. The authors briefly acknowledged this in L364, but they can explicitly state that integrating physical processes or spatial information is a promising direction for future research. Although EDM is a novel time series analysis approach, ecological statistics should ultimately be spatiotemporal. The integration of spatial information in EDM (spatial EDM) is an exciting direction for future research (e.g., https://doi.org/10.1111/2041-210X.13511).

Minor points
- Abstract: It is better to mention that the authors conducted "causal analysis," not "correlation analysis," based on nonlinear time series analysis.

- Line 95: The authors may clarify that they performed time-series-based causal detection analysis and used "predicted" daily physical parameters here.
- Line 123: It is better to say " the natural logarithm" is taken instead of "Ln-transformed" for clarity.
- Line 134: The authors may introduce the term "empirical dynamic modeling (EDM)" here.
- Line 205: Figure 2 indicates that the benchmark model (persistence model) outperformed the MDR S-map models when the forecast horizon is from 0 to about 5 days (or sometimes more). Does this suggest that the MDR S-map did not provide meaningful predictions for the 0-5 day period? Also, is this related to the smoothing of the original time series (as the smoothing may strengthen the autocorrelation)? What would happen if EDM were applied to the original time series?
- Line 357: The term "empirical dynamic modeling" should be introduced earlier.
- Line 360: The term "embedding dimension" was not explained.
- Line 517: Why was the embedding dimension fixed at 15? It should be determined for each variable.
- Line 531: The term "EDM" was not defined before.
- Line 532: CCM was conducted using TP=0, while the MDR S-map was conducted using TP=1. Why didn't the authors use TP=-1 for CCM?
- Figure 1A: It should be clarified on the y-axis that these are also "Ln-transformed" values.

********************END********************

Version 1:

Reviewer comments:

Reviewer #3

(Remarks to the Author)

Reviewer #4

(Remarks to the Author)
I have reviewed the revised manuscript, “Biotic interactions shape the realised niche of toxic cyanobacteria,” by Ntetsika et al., submitted to Nature Ecology & Evolution. The authors have carefully and thoroughly addressed my previous comments, substantially improving the study’s robustness. I would like to congratulate the authors on this impressive work. I have no further comments, and I now have no hesitation in recommending this paper for publication.

Decision Letter:

10th March 2026

Dear Dr. Ntetsika,

Thank you for submitting your revised manuscript "Biotic interactions shape the realised niche of toxic cyanobacteria" (NATECOLEVOL-25103591A). It has now been seen again by the original reviewers and their comments are below. The reviewers find that the paper has improved in revision, and therefore we'll be happy in principle to publish it in Nature Ecology & Evolution, pending minor revisions to satisfy the reviewers' final requests and to comply with our editorial and formatting guidelines.

If the current version of your manuscript is in a PDF format, please email us a copy of the file in an editable format (Microsoft Word or LaTex)-- we can not proceed with PDFs at this stage.

If you have not done so already, please ensure that you also email us a completed copy of the Reporting summary :

Reporting summary: <a href="https://www.nature.com/documents/nr-reporting-summary.pdf">https://www.nature.com/documents/nr-reporting-summary.pdf</a>

We are now performing detailed checks on your paper and will send you a checklist detailing our editorial and formatting requirements in about a week. Please do not upload the final materials and make any revisions until you receive this additional information from us.

Thank you again for your interest in Nature Ecology & Evolution. Please do not hesitate to contact me if you have any questions.

Sincerely,

[redacted]

Reviewer #4 (Remarks to the Author):

I have reviewed the revised manuscript, “Biotic interactions shape the realised niche of toxic cyanobacteria,” by Ntetsika et al., submitted to Nature Ecology & Evolution. The authors have carefully and thoroughly addressed my previous comments, substantially improving the study’s robustness. I would like to congratulate the authors on this impressive work. I have no further comments, and I now have no hesitation in recommending this paper for publication.

**Open Access** This Peer Review File is licensed under a Creative Commons Attribution 4.0 International License, which permits use, sharing, adaptation, distribution and reproduction in any medium or format, as long as you give appropriate credit to the original author(s) and the source, provide a link to the Creative Commons license, and indicate if changes were made.
In cases where reviewers are anonymous, credit should be given to 'Anonymous Referee' and the source.
The images or other third party material in this Peer Review File are included in the article’s Creative Commons license, unless indicated otherwise in a credit line to the material. If material is not included in the article’s Creative Commons license and your intended use is not permitted by statutory regulation or exceeds the permitted use, you will need to obtain permission directly from the copyright holder.
To view a copy of this license, visit https://creativecommons.org/licenses/by/4.0/

# Point-by-point response for

**Biotic interactions shape the realised niche of toxic cyanobacteria**

Pinelopi Ntetsika, Stefanie Eyring, Ewa Merz, Marta Reyes, Benno Käch, Stephan Munch, Stuart Dennis, Marco Baity-Jesi, Francesco Pomati

Corresponding author: Pinelopi Ntetsika, pinelopi.ntetsika@eawag.ch

**Reviewer #3**

The authors greatly improved their manuscript, and they clarified many of the points raised. Overall, I still think this is great work, as it is based on high frequency plankton data and provides a unique opportunity to generate understanding on plankton dynamics. It strongly hinges on data analyses, which has been done very thorough.

We thank the reviewer for their continued engagement with our manuscript and thoughtful suggestions. We have carefully considered all comments and made revisions where they strengthen the manuscript. However, some suggestions would require substantial expansions in scope or length that are not feasible given the manuscript is already at the word limit and represents a synthesis of multiple taxa and methodological developments. Following the editor's guidance, we have identified requests that we believe are impractical or beyond the scope of this study. Below, we address each comment individually and explain our reasoning where we have declined suggested changes.
***For clarity, please note that all line numbers referenced in our responses correspond to the Microsoft Word document with tracked changes and reviewer comments visible.**

While I am not an expert, there are a few choices and their consequences that remain elusive. Overall, the authors seem to oversell their work by emphasizing toxic cyanobacteria, and in relation to this, some conclusions seem to stretch it. This is a pity, as the work does not need this. It is strong in providing insights in complex plankton dynamics in natural systems, whatever the chosen phytoplankton group. Indeed, by focusing on net growth rates, it is the dynamics that is of interest, while risks of cyanobacterial blooms are not related to their growth, but rather population densities as those more generally link to toxins (and hence the risk).

We appreciate the reviewer's positive assessment of our work and their recognition of the insights it provides into complex plankton dynamics. We fully agree on the potential impact on the fundamental understanding of plankton ecology that our approach can convey. We do not think that emphasising toxic cyanobacteria represents 'overselling' our work. While we agree that our methodological framework applies broadly to plankton dynamics, the choice to focus on bloom-forming cyanobacteria is strategic and well-justified for several reasons:

First, cyanobacterial blooms represent one of the most pressing water quality challenges globally, with direct impacts on drinking water safety, recreation, and ecosystem services. Understanding the processes governing cyanobacterial accumulation dynamics has clear societal relevance and management implications.

Second, the reviewer correctly notes that risks are associated with population densities that produce toxins. However, these densities are the direct result of net growth dynamics—the very processes we quantify. One cannot understand or predict bloom formation without understanding the fundamental growth processes that lead to biomass accumulation. Our focus on net growth rates provides the mechanistic foundation for understanding how blooms develop, which is essential for any predictive or management framework.

Third, the regulatory thresholds for cyanobacterial risk indeed vary by water use (drinking water, recreation, etc.), but all are fundamentally tied to cell densities achieved through the net growth processes we characterise. By revealing how biotic and abiotic factors interact to govern these

processes, we provide insights directly relevant to understanding when and where these thresholds are likely to be exceeded.

We believe our emphasis on cyanobacteria appropriately highlights the applied relevance of fundamental ecological insights. The work stands on its methodological and conceptual merits while also addressing a critical environmental challenge.

Below I listed some of my remaining concerns:
1) In answer to my question why cyanobacterial net growth rates were compared to biomass of other plankton groups, the authors refer to the classical Lotka-Volterra concept. They state that growth rate (as a process) depends on densities (as state variables). This is where to me the terminology gets confusing. While I see the intrinsic growth rate as a process, the per capita growth rate is not (as it is the net change in population density following from gains and losses). To me, it seems that inferring the intrinsic growth rate of cyanobacteria would involve relating the per capita change of cyanobacteria with their abundance. From the used calculations, it seems the authors calculate the intrinsic growth rate directly, and then use this as analogue to per capita change for comparison with the other population densities (used as state variables). This needs further clarification, and perhaps some indication on the consequence of this. I understand the method has been used for an earlier study (Nguyen et al. 2025, PNAS), but that study is constrained to phytoplankton groups, compares the intrinsic growth rates across all involved groups, and includes some clear constraints of the approach.

We appreciate the reviewer's attention to this important methodological detail and welcome the opportunity to clarify the distinction between our approach and that of Nguyen et al. (2025, PNAS). We agree that terminology can be confusing here. In our study, we model the net per capita growth rate (denoted as nGR), which represents the balance between intrinsic growth and all loss processes (grazing, parasitism, competition, etc.). This is indeed a rate of population change, not a static process parameter. The reviewer is correct that this differs from the intrinsic growth rate (r), which is a process parameter in classic Lotka-Volterra formulations.

The key distinction between our study and Nguyen et al. (2025) lies in our objectives and corresponding model structures:

- Nguyen et al. (2025, PNAS): Focused on estimating Lotka-Volterra parameters (intrinsic growth rates and interaction coefficients) to study species interactions and coexistence. The model explicitly excludes abiotic variables, making the intercept interpretable as the intrinsic growth rate (r).
- This study: Focused on mapping the realised niche—the environmental conditions under which species achieve positive net growth. This requires including both biotic (population densities) and abiotic variables (temperature, nutrients) as predictors. Consequently, the intercept is no longer the intrinsic growth rate but rather a baseline that shifts with environmental context.

Following Lotka-Volterra principles, net (per capita) growth rates are functions of population densities (state variables). In our models, we incorporate lagged cyanobacterial abundance to account for density dependence, while other plankton densities capture biotic interactions (grazing, facilitation). This allows us to estimate density-independent coefficients that reveal how environmental conditions and species interactions jointly shape the realised niche.

We believe presenting both model formulations side-by-side would confuse rather than clarify our specific contribution. The Lotka-Volterra parameter estimation approach is already thoroughly described in Nguyen et al. (2025). Our focus here is fundamentally different: characterising how net growth responds to the full suite of biotic and abiotic conditions that define the realised niche in natural systems. However, if the reviewer and editor believe readers would benefit from an explicit comparison of these two modeling frameworks and their respective purposes, we are willing to add this discussion to the Methods section of the Supporting Information.

2) I understand how facilitation is concluded, but still do not see how this can be differentiated from co-occurrence, and/or inversed directionality where cyanobacteria facilitate other taxa? While I understand that the focus of the current study is not about the role of cyanobacteria on other plankton taxa, the authors do infer the direction from their findings. This needs to be more clearly emphasized.

While we understand the importance of the reviewer's question, we don't think that the directionality of effects requires further clarification in the manuscript. Our analytical framework is explicitly designed to infer causality and directional effects through two complementary approaches:

1. Convergent Cross Mapping (CCM) establishes causal directionality: As shown in **Fig. 1E-F** (and Extended Data **Fig. E2**) and described in our methods, CCM tests for causality by reconstructing the time series of one variable from the attractor of another. Critically, if X causally forces Y, then information about X will be embedded in Y's dynamics, allowing us to successfully predict X from Y's attractor (X causes Y is tested by cross-mapping from Y to X). This asymmetric property distinguishes true causal influences from simple correlations or co-occurrence. Our CCM analyses specifically tested and confirmed the direction of effects between other plankton taxa or abiotic variables and cyanobacterial nGRs.
2. S-map models formalise temporal precedence: Following the empirical dynamic modeling (EDM) framework and consistent with Lotka-Volterra formulations, our S-map models explicitly relate the future net growth rates at time t+1 to explanatory variables (both biotic and abiotic) at time t. This lagged structure ensures temporal precedence—a fundamental criterion for causality. The model quantifies how population densities and environmental conditions at t influence future cyanobacterial net growth at t+1, not the reverse.

Together, these approaches establish directionality: CCM identifies which species interactions are causal rather than coincidental, and S-maps quantify how those causal drivers influence cyanobacterial dynamics. The combination provides strong evidence for the directionality we report.

Regarding the reviewer's question about whether cyanobacteria might facilitate other taxa: while this is an interesting question for future research, it is outside the scope of our current study, which focuses specifically on the drivers of cyanobacterial net growth dynamics. Our analytical framework would allow testing reverse directionality (effects of cyanobacteria on other taxa), but that would constitute a separate analysis with different response variables—namely, the net growth rates of those other taxa. Such analyses would be a logical extension of this work but are not necessary to establish the conclusions we draw here about cyanobacterial dynamics.

We believe our methods section, results and figures adequately describe the EDM analytical approach. Adding further discussion of directionality would be redundant and risk obscuring the clarity of our current presentation.

3) The risk of cyanobacterial blooms is mainly linked to their biomass, as that often is well correlated with the toxin concentrations (which is the reason why they form a risk). This is not the same as positive growth rates, and one may even argue that at the onset of a bloom, growth may be high but the risk is still low. While at the peak of a bloom, the growth is low, but the risk is high. Specific outcomes, such as thresholds (L371) or monitoring (L395-401), seem too farfetched. There would be sufficient novelty when focusing on plankton dynamics, and I would strongly suggest to tone down those parts.

We thank the reviewer for this important clarification. We recognise that our use of 'threshold' was ambiguous and could be misinterpreted, and revised the paper accordingly.

When we refer to 'thresholds' (e.g. L371), we mean the conditions under which net growth transitions from negative to positive—that is, the boundary in environmental space where biomass accumulation begins (the zero net growth isocline). This is fundamentally different from the biomass-based thresholds used in risk assessment or management (e.g., cell density limits for drinking water or recreational use). We will revise this terminology throughout to avoid confusion, using phrases such as 'conditions for positive net growth' or 'boundaries' instead of 'thresholds.' These changes are implemented in the revised version of the manuscript.

The reviewer is also correct that bloom risk relates to achieved biomass levels, not growth rates per se. However, understanding when and where positive net growth occurs is a necessary precondition for predicting biomass accumulation patterns—even if it does not directly translate to risk assessment. We agree that we overstated the immediate management applications. We revised the relevant sections as follows:

Lines 371-75 (now L383–386): *'The empirical approach we developed to quantify how biotic interactions shape the conditions for positive net growth provides a framework that could be adapted to other freshwater ecosystems, recognising that specific species interactions will vary across systems.'*

Lines 395-401 (now L428–435): *'Our findings suggest potential directions for future monitoring and management. High-frequency plankton data could complement traditional water quality measurements by revealing dynamic biotic interactions that influence bloom development. Understanding how key planktonic taxa affect cyanobacterial net growth—such as the roles we identified for rotifers and chrysophytes—may eventually inform management strategies that extend beyond nutrient control alone. However, translating these mechanistic insights into operational management tools will require further validation across diverse systems and explicit testing of the hypotheses that emerged from our study.'*

This revised framing maintains the novelty and importance of our work while appropriately acknowledging that direct management applications remain prospective rather than immediately actionable.

Rather, I would like to see some words on how the current approach would look like when targeting other groups (at least in discussion) in relation to cyanobacteria, especially given that other groups are often more dominant in lake Greifensee; as more clearly shown in Nguyen et al. 2025).

We appreciate the reviewer's interest in how our approach could be extended to other taxonomic groups in Lake Greifensee. We share this enthusiasm—indeed, applying our framework to less-studied taxa such as chrysophytes could yield exciting ecological insights given how little is known about their realised niche, species interactions and dynamics in natural systems.

However, we are afraid that such an expansion is beyond the scope of the current manuscript for several reasons:

- The current study is already substantively complex, integrating novel data collection, empirical dynamic modelling, and niche mapping on four taxa. Over two rounds of revision, we have added significant methodological developments and validations (now documented across multiple Supporting Information sections) in response to reviewer feedback. The manuscript essentially contains the methodological rigour of multiple studies synthesised into a cohesive framework. We are at the word limit, and we don't honestly know how to accommodate additional discussion.
- Our focus on bloom-forming cyanobacteria provides a clear, ecologically and societally relevant case study that ties together methodological innovation with specific biological insights. Broadening the discussion risks diluting our findings without adding new results.
- We are actively pursuing follow-up studies applying this framework to other dominant taxa in Lake Greifensee, including the ecologically understudied chrysophytes mentioned earlier. These analyses merit dedicated treatment where we can provide the same analytical depth and ecological interpretation that we have given to cyanobacteria in the current study.

We believe the current manuscript makes a strong, focused contribution by establishing and validating the methodological framework while providing deep insights into cyanobacterial dynamics. The applicability to other taxa is implicit in the generality of the approach, and we are committed to demonstrating this in subsequent publications.

4) The term 'Toxic cyanobacteria' in the title and 'Toxic Microcystaceae' is misleading, as it would suggest the authors actually assessed toxins. Why not focus on Microcystis and Dolichospermum, and, as currently already done, show the other genera in the suppl.

We appreciate the reviewer's suggestion regarding terminology. However, we should maintain our current nomenclature for the following reasons:

- In the first round of review, **Reviewers 1 and 3** specifically requested that we use 'Toxic Microcystaceae' rather than focusing solely on the genus Microcystis, which is ambiguous both empirically from our data and phylogenetically. This change was made mostly to reflect the taxonomic resolution that our automated microscopy approach can afford: morphologically similar taxa within the Microcystaceae family, not exclusively Microcystis cells. Reverting to genus-level terminology would contradict this previous feedback and misrepresent the taxonomic resolution of our data.
- The designation 'toxic' is not speculative but based on direct measurements. As stated in the main text (before L111-112, now L111-113): *'Previous studies confirm the production of microcystins and numerous other bioactive metabolites from this group in the lake, including during our study period.'* Specifically, Wang et al. (2024, Environ. Sci. Technol.; reference 21) measured toxins from Lake Greifensee water samples, isolated cyanobacterial strains (including Toxic Microcystaceae), and confirmed their toxicity through laboratory testing during our study period. This work provides robust empirical support for the 'toxic' designation.

- The term 'toxic cyanobacteria' in the title accurately reflects both the taxonomic identity and the ecological/societal significance of the organisms we study. Removing this descriptor would diminish the applied context that makes this work relevant to water quality management and public health, without improving scientific accuracy.

We believe our current terminology appropriately balances taxonomic precision (Microcystaceae rather than Microcystis alone), empirical support (documented toxin production), and ecological relevance (toxic bloom risk). Changing this nomenclature at this stage would create inconsistency with previous revisions and potentially impair  the taxonomic scope and applied significance of our study.

**Reviewer #4**

I have reviewed the manuscript titled "Biotic interactions shape the realised niche of toxic cyanobacteria" by Ntetsika et al., submitted to Nature Ecology & Evolution. The authors analyzed unique ecological time series from Lake Greifensee in Switzerland to understand how the net growth rate (nGR) of potentially harmful cyanobacterial groups is affected by biotic and abiotic factors. The authors utilized specific time series analytical tools, called empirical dynamic modeling (EDM), to address the nonlinearity and high dimensionality of ecological time series. They found that the thresholds of physical variables (e.g., temperature and nutrients) enabling nGR > 0 (defined by the authors as the "realised niche") varied depending on the abundance of co-occurring plankton taxa, suggesting that biotic interactions can modify the response of cyanobacterial groups to physical parameters. My expertise lies in general community ecology and statistical ecology, and I have limited knowledge of the ecology and biology of specific freshwater plankton taxa. Therefore, my comments mostly on the logical flow and statistical analyses.

Overall, I am quite positive about this manuscript. Although the idea that the abundance of cyanobacteria is determined by both abiotic and biotic factors is not new (as also pointed out by the original Referee #2), the "quantitative" evaluation of how biotic factors alter the physical thresholds is novel (as far as I know). This is particularly challenging under field conditions, and the authors achieved this by utilizing recently developed nonlinear time series analysis methods. Their findings are ecologically meaningful and can contribute to more accurate predictions of harmful algal blooms.

I have also read the comments from the original referees and the authors' responses. I agree with most of the original referees' comments and believe that careful wording is necessary to communicate the ideas accurately. The authors have revised terms such as "response" and "fundamental" and have clearly defined the concept of "realised niche." Based on my review of the revised manuscript, the concept is now clear.

We sincerely thank the reviewer for their positive evaluation and for recognising the novelty of our quantitative approach to understanding how biotic interactions modify physical thresholds for cyanobacterial net growth. We particularly appreciate the reviewer's acknowledgement that achieving such quantification under natural field conditions is challenging, and that our application of empirical dynamic modelling methods represents a meaningful contribution to predicting harmful algal blooms. We are also grateful for the reviewer's expertise in empirical analysis methods, which has helped ensure the logical flow and analytical rigour of our manuscript.
***For clarity, please note that all line numbers referenced in our responses correspond to the Microsoft Word document with tracked changes and reviewer comments visible.**

Although I am generally positive and understand that the authors have already done a substantial amount of work, however, I still have several concerns about the data analysis. I believe these concerns should be addressed before the manuscript is accepted.

1. The authors smoothed the plankton densities using local polynomial regression to remove random noise from the original time series (L477 in the methods section). While smoothing is useful for capturing the general trend in time series, I wonder whether it is appropriate to apply it to a chaotic time series (as the authors stated in their response letter that they are "chaotic"). If the dynamics are governed by linear processes with some random errors, smoothing can effectively remove these errors. However, if the dynamics are governed by a nonlinear deterministic process, smoothing may

eliminate ecologically meaningful information from the time series. The authors can test whether smoothing influences the outcome of this analysis using at least two approaches: (i) analyzing the raw data and comparing the results to those from the smoothed time series, and (ii) generating artificial time series (for example, from simple differential equations, such as those used in Sugihara et al. 2012 Science), applying a smoothing function, and conducting EDM. The second approach is particularly effective for assessing the appropriateness of the smoothing method. I believe the authors should demonstrate that smoothing does not affect the final conclusions of this manuscript.

We thank the reviewer for this important and detailed comment, which we followed up with additional analyses. We agree that in chaotic or nonlinear time series, inappropriate smoothing could potentially remove ecologically meaningful dynamics. Our goal, however, is to remove **measurement noise (stochastic noise)**—which is distinct from process noise and can be present in both chaotic and non-chaotic systems—without affecting the underlying deterministic dynamics. This is particularly important for abundance time series because measurement noise can be amplified during growth-rate calculations and in the estimation of interaction coefficients via S-maps, which involve derivatives.

To ensure the appropriateness of our smoothing approach, we carefully selected the local polynomial regression (loess) span by examining autocorrelation in the residuals, following established practices in the literature. Nonetheless, in response to the reviewer's suggestion, we conducted additional analyses to explicitly test whether smoothing affects the results:

1. **Artificial time series simulation:** We generated in-silico time series using the three-species Hastings–Powell model (Hastings & Powell 1991), which exhibits chaotic dynamics. We added Gaussian noise to the time series at levels corresponding to the 25th, 50th, and 75th percentiles of the empirical variance observed in our plankton data (**Fig. R1**). After log-transforming the series, we applied the same loess smoothing (span = 0.03) as in our manuscript. We then fitted regularised S-map models to predict growth rates and inferred interaction coefficients under all combinations of noise addition and smoothing, resulting in 21 conditions per target variable.

redacted

**Fig. R1: Simulated Hastings–Powell time series with added measurement noise.** Simulated time series from the three-species Hastings–Powell food-chain model. Panels show the original noise-free time series ("Original") and the same series after the addition of Gaussian noise with mean 0 and variance corresponding to the 25th (var = 0.19), 50th (var = 0.32), and 75th (var = 0.50) percentiles of the empirical variance estimated from the observational plankton data. Species are indicated by colour: x (blue), y (black), and z (red). These time series illustrate the increasing noise levels used in the in-silico experiments before optional noise reduction via loess smoothing.

2. **Comparison of performance and coefficient estimation: Figs. R2-R5** summarise the results.

redacted

**Fig. R2: Model performance comparison.** Comparison of the top 5% of S-map models, assessed using RMSE (top panels) and Pearson correlation coefficient, rho (bottom panels). Model performance was evaluated using sequential leave-future-out cross-validation (as in the manuscript). The x-axis indicates the level of Gaussian noise added to the time series (mean = 0, variance = var0.19, var0.32, var0.5 corresponding to the 25th, 50th, and 75th percentiles of the empirical variance, respectively), while “original” represents performance using the raw, unmodified time series. Rows correspond to the three target variables in the Hastings–Powell model (x, y, z). Colours indicate whether noise reduction via loess smoothing was applied (red = smoothed; blue = unsmoothed).

redacted

**Fig. R3: Accuracy of inferred interaction coefficients for target variable X.** Correlation of inferred S-map coefficients for the target variable X from the Hastings–Powell model. Each panel shows results from the best-performing S-map model (lowest RMSE). The x-axis displays the S-map coefficient for a given predictor variable in the noisy time series (with Gaussian noise added, mean 0 and variance 0.19, 0.32, and 0.5), and the y-axis shows the corresponding coefficient from the original, noise-free time series. Colours indicate whether noise reduction via loess smoothing was applied (red = smoothed; blue = unsmoothed). Above each panel, the Spearman correlation coefficient between coefficients from the original time series and those from smoothed or unsmoothed noisy series is reported, indicating how well the smoothing preserves the true interactions. The results indicate that estimation of interaction coefficients is more accurate for smoothed time series compared to unsmoothed series, especially as noise increases. Coefficients from smoothed series remain close to the ground truth, whereas coefficients from noisy, unsmoothed series deviate progressively with higher noise levels. Coefficients are rescaled to the [0,1] range within each panel using min–max normalisation.

redacted

**Fig. R4: Accuracy of inferred interaction coefficients for target variable Y.** Correlation of inferred S-map coefficients for the target variable y from the Hastings–Powell model. Each panel shows results from the best-performing S-map model (lowest RMSE). The x-axis displays the S-map coefficient for a given predictor variable in the noisy time series (with Gaussian noise added, mean 0 and variance 0.19, 0.32, and 0.5), and the y-axis shows the corresponding coefficient from the original, noise-free time series. Colours indicate whether noise reduction via loess smoothing was applied (red = smoothed; blue = unsmoothed). Above each panel, the Spearman correlation coefficient between coefficients from the original time series and those from smoothed or unsmoothed noisy series is reported, indicating how well the smoothing preserves the true interactions. The results indicate that estimation of interaction coefficients is more accurate for smoothed time series compared to unsmoothed series, especially as noise increases. Coefficients from smoothed series remain close to the ground truth, whereas coefficients from noisy, unsmoothed series deviate progressively with higher noise levels. Coefficients are rescaled to the [0,1] range within each panel using min–max normalization.

redacted

**Fig. R5: Accuracy of inferred interaction coefficients for target variable z.** Correlation of inferred S-map coefficients for the target variables y from the Hastings–Powell model. Each panel shows results from the best-performing S-map model (lowest RMSE). The x-axis displays the S-map coefficient for a given predictor variable in the noisy time series (with Gaussian noise added, mean 0 and variance 0.19, 0.32, and 0.5), and the y-axis shows the corresponding coefficient from the original, noise-free time series. Colours indicate whether noise reduction via loess smoothing was applied (red = smoothed; blue = unsmoothed). Above each panel, the Spearman correlation coefficient between coefficients from the original time series and those from smoothed or unsmoothed noisy series is reported, indicating how well the smoothing preserves the true interactions. The results indicate that estimation of interaction coefficients is more accurate for smoothed time series compared to unsmoothed series, especially as noise increases. Coefficients from smoothed series remain close to the ground truth, whereas coefficients from noisy, unsmoothed series deviate progressively with higher noise levels. Coefficients are rescaled to the [0,1] range within each panel using min–max normalization.

These results demonstrate that smoothing effectively removes measurement noise without fundamentally altering the inferred ecological interactions. For variable X, the benefit of smoothing is particularly clear: interaction coefficients are more accurately recovered across all noise levels. For variables Y and Z, the patterns are more nuanced—particularly for Z, which exhibits naturally weak interaction coefficients—but the key finding remains consistent: smoothing preserves the relative strength and sign of interactions. Strong interactions remain strong, weak interactions remain weak, and the rank ordering of effect sizes is maintained. Importantly, at moderate to high noise levels

(corresponding to the 50th and 75th percentiles of variance in our empirical data), smoothing consistently improves coefficient estimation compared to analysing raw data, even for chaotic dynamics.

This analysis confirms that our smoothing procedure accomplishes its intended purpose: removing measurement noise that would otherwise be amplified in derivative-based calculations (net growth rates) and interaction coefficient estimation (S-maps), without eliminating the deterministic signal underlying plankton dynamics. The ecological conclusions of our manuscript—particularly the identification of key biotic interactions and their modification of physical thresholds—remain robust to this methodological choice. We thank the reviewer for prompting this validation. **Methodological details** of the artificial time series analysis have been added to the **Supplementary Information** to ensure transparency and reproducibility (see **Supplementary Figs. S21–S25** and **Supplementary Methods**), while the analysis is briefly mentioned in the **main Methods** (**Section 2.5 – Smoothing of daily data**).

2. Most of the physical parameters were predicted using a Random Forest (RF) approach. While I totally understand that daily measurements of these parameters are currently impractical, I am concerned that the choice of prediction method may affect the results. Is it possible to show how the choice of prediction methods influences the outcome of EDM? One suggestion for the authors is to use only the real measurement values (i.e., weekly data), conduct EDM, and compare it to the results of the daily time series analysis. Although analyzing weekly data may change the interpretation of the results (as the time scale of the dynamics changes from daily to weekly), this type of validation could demonstrate the robustness of the analysis. Also, the relevant topic of interpolating missing values was studied in Carpenter et al. (2025) in "Repairing Gaps in Ecological Time Series" (https://doi.org/10.1111/2041-210X.14491 just for your information).

We thank the reviewer for this thoughtful and technically important comment. We agree that the method used to predict daily values of physical and chemical parameters from weekly observations could, in principle, influence downstream empirical dynamic modelling (EDM) results, and we appreciate the opportunity to clarify and test this aspect of our workflow.

First, we would like to clarify that we did not interpolate daily nutrient concentrations directly from weekly observations. Instead, the Random Forest (RF) models were trained on weekly data to learn relationships between nutrient concentrations (targets) and contemporaneous physical, chemical, and meteorological variables (predictors). These trained models were then used to predict nutrient concentrations at a daily resolution using daily predictor data. Thus, the RF approach functions as a data-driven regression model rather than a temporal interpolation scheme.

We selected the RF algorithm because it is well-suited for this task: (i) it can accommodate a large number of explanatory variables, (ii) it is relatively robust to collinearity among predictors, which is common in abiotic variables with strong seasonal components, and (iii) it allows for nonlinear and nonparametric relationships without requiring strong distributional assumptions. Cross-validation and independent testing showed good predictive performance for all nutrients (**see Eyring et al. 2005, Supplementary Table S4 for details**), giving us confidence that daily nutrient dynamics are captured realistically.

The reviewer's suggestion to repeat the EDM analysis using only weekly observations is well taken. However, this would fundamentally change the temporal scale of the analysis and the interpretation of the results. Our study focuses on **daily net growth processes**, for which key mechanisms—such as

grazing losses and short-term biotic interactions—operate at daily timescales. Consistent with this, our CCM results show that biotic variables dominate causal effects at daily scales, whereas abiotic drivers exert stronger influence at seasonal scales. A weekly-scale analysis would therefore address a different ecological question and is unlikely to be directly comparable to the present results.

To directly assess whether our results are sensitive to the choice of dissolved nutrients' prediction method, we conducted an additional robustness analysis using an S-map-based approach, analogue to the RF, to predict daily nutrient concentrations from weekly observations. This analysis mirrors the RF workflow but replaces the prediction algorithm with S-maps. We then compared (i) the daily nutrient predictions generated by the RF and S-map models and (ii) their predictive performance.

**Methodological description: S-map-based nutrient prediction.** To assess the sensitivity of our results to the choice of nutrient prediction method, we conducted an analogous analysis using the S-map algorithm to predict daily nutrient concentrations.

The initial dataset consisted of weekly observations of the three target nutrients (ammonium, nitrate, and phosphate), along with weekly measurements of physical variables in the photic zone (temperature, conductivity, dissolved oxygen, pH, PAR light penetration depth, and chlorophyll *a*) and meteorological variables (minimum and maximum wind speed, air temperature, air pressure, and total precipitation) from Lake Greifensee. This dataset was identical to that used for training and testing the Random Forest models.

The weekly dataset was split into training (80%) and testing (20%) subsets. Within the training set, we performed 10-fold cross-validation to select optimal S-map parameters, using the same parameter ranges as in the main EDM analysis (**Methods Section 3.4**). For each nutrient, the parameter combination yielding the lowest RMSE was selected. Model performance was then evaluated by predicting the withheld test set.

Following validation, the S-map model with optimal parameters was fitted to the full weekly dataset and used to predict daily nutrient concentrations based on daily observations of physical and meteorological predictor variables. These daily predictions were compared with those generated by the Random Forest models in terms of correlation and predictive accuracy.

**Results:** As shown in **Figs. R6 and R7**, daily nutrient predictions from the two approaches are highly correlated, and their predictive performance is comparable across nutrients. These results indicate that our EDM findings are not driven by idiosyncrasies of the RF algorithm but are robust to the choice of prediction method. We therefore conclude that re-running the full EDM analysis using an alternative prediction approach is unlikely to alter the main conclusions of the study.

redacted

**Fig. R6: Agreement between daily nutrient predictions from Random Forest and S-map models.** Correlation between daily nutrient predictions generated by the Random Forest and S-map models for ammonium, phosphate, and nitrate. Daily predictions were produced using the best-performing Random Forest and S-map models, as selected by 10-fold cross-validation. Agreement between methods was quantified using Spearman's rank correlation coefficient. Correlations were high for all nutrients (nitrate: $\rho = 0.85$; phosphate: $\rho = 0.79$; ammonium: $\rho = 0.89$), indicating strong consistency between the two prediction approaches.

redacted

**Fig. R7: Comparison of predictive performance between Random Forest and S-map models.** Predictive performance of the Random Forest and S-map models for daily nutrient estimation. **The top panel** shows Pearson correlation coefficients (rho) between observed and predicted values for each target nutrient, evaluated using 10-fold cross-validation (CV) and an independent test set (20% of the data). Points indicate mean rho across CV folds, and error bars represent ±1 standard deviation; test-set performance is shown as a single point. **The bottom panel** shows corresponding root mean squared error (RMSE) values for CV and test data. Red symbols correspond to the Random Forest model and blue symbols to the S-map model.

We have conducted this comparative analysis to address the reviewer's concern about methodological robustness. However, we believe this material is most appropriate for the rebuttal letter rather than the manuscript itself for the following reasons:

- First, the high correlation between RF and S-map predictions and comparable predictive performance demonstrate that our choice of prediction method does not influence our results. This validates our approach but does not add new ecological insights relevant to the manuscript's scope and findings.
- Second, the manuscript already includes extensive methodological validation and sensitivity analyses in the Supporting Information (smoothing effects, embedding dimension sensitivity, exclusion radius effects, seasonal surrogate tests). Adding yet another methodological comparison would further increase the already substantial SI without enhancing interpretation of the ecological results.

We therefore believe this comparative analysis serves its purpose as a robustness check in response to the reviewer's specific concern, but inclusion in the manuscript would be redundant and would detract from the ecological focus of the study.

3. Technical Details of CCM. In the original CCM paper (Sugihara et al. 2012), two important criteria are used to assess causality. The first is rho (forecasting skill) at the largest library size. The second is "convergence," which measures the difference between rho at the largest library size and that at the smallest library size. This convergence checks whether the reconstructed trajectories ultimately reach a 1:1 mapping and helps determine if the observed cross-map skill is due to pseudo-correlation or not. The authors did not assess this "convergence" in the manuscript. I believe that the surrogate test of rho at the largest library size alone is insufficient to exclude the possibility of pseudo-correlation, and they should also check for convergence. Alternatively, the authors could utilize a more recently developed method called "unified-information theoretic causality" (https://doi.org/10.1101/2023.04.20.537743), which integrates transfer entropy and CCM and does not require testing for convergence.

We thank the reviewer for this helpful and technically important comment. We agree that, in the original CCM framework (Sugihara et al. 2012), causality is assessed not only by cross-map skill (rho) at large library sizes but also by convergence of rho with increasing library size, which helps distinguish true causal interactions from spurious correlations.

In the original version of the manuscript, our primary goal was to quantify the strength of coupling between variables rather than to make strong causal claims, given that planktonic communities are highly interconnected systems. Nevertheless, in response to the reviewer's suggestion, we have now explicitly implemented the CCM convergence test.

Specifically, we assessed convergence for both the original time series and the 100 seasonal surrogate series. For each pair of variables, we estimated the mean cross-map forecasting skill (rho) at a minimum library size (10% of the data) and a maximum library size (80% of the data), using 100 random subsamples at each library size. We selected 80% as the maximum library size to allow for a sufficient number of subsamples and to better quantify uncertainty. Convergence was evaluated by comparing rho at small versus large library sizes at Tp=-1 (forecast horizon, see **minor comment 10**).

All tested variable pairs—both original and seasonal surrogate time series—exhibited increasing cross-map skill with library size, satisfying the convergence criterion (**Fig. R8**). Combined with our existing surrogate tests assessing whether the original time series consistently achieve higher forecasting skill at large library sizes than the seasonal surrogates, these results meet both key CCM criteria and provide stronger evidence that the detected interactions reflect causal relationships rather than pseudo-correlation.

We extensively updated the CCM analysis (see also the reply to **minor comment 8** regarding embedding dimension) and our new results are consistent with those reported in the previous version of the manuscript, but we gained substantial robustness thanks to the reviewer's input. In particular, the strongest causal links affecting cyanobacterial growth rates at the daily scale are confirmed to be predominantly biotic variables, whereas abiotic variables exhibit the strongest seasonal effects.

We have updated the CCM methodology accordingly in **Section 3.1** of the Methods, **Fig. 1 E-F** and Extended Data **Fig. E2 C-D** showing the CCM results and included the convergence test results as a **new Supplementary Figure (Fig. S26) for embedding dimension 10** (see **minor comment 8** for the explanation).

redacted

**Fig. R8: CCM convergence test for cyanobacterial growth drivers.** Convergence of cross-mapping forecasting skill (rho) was assessed for all target cyanobacteria and all predictor variables included in the original S-map. CCM was performed using an embedding dimension $E = 15$ and prediction horizon $TP = -1$. For each variable pair, rho was estimated at a minimum library size (10% of the time series) and a maximum library size (80% of the time series) using 100 random subsamples. Convergence was evaluated using a one-sided t-test testing whether the mean rho at the maximum library size was greater than the mean rho at the minimum library size. P-values from the convergence test are shown in **orange** for the original time series and in **blue** for the 100 seasonal surrogate time series. The dashed horizontal line indicates the significance threshold ($p = 0.05$). All original time series and more than 96% of the seasonal surrogate time series exhibit p-values $< 0.05$, indicating significant convergence of cross-map skill with increasing library size.

4. The authors carefully discussed the limitations of their study in response to the comments from the original referees. While they have addressed several important topics, they may also comment on the inclusion of physical processes such as water circulation and waves. As Referee #3 pointed out, the

study is based on a single location, but monitoring multiple locations and utilizing spatiotemporal information would definitely improve prediction accuracy, especially in more open and dynamic marine systems where harmful algal blooms have also been observed. The authors briefly acknowledged this in L364, but they can explicitly state that integrating physical processes or spatial information is a promising direction for future research. Although EDM is a novel time series analysis approach, ecological statistics should ultimately be spatiotemporal. The integration of spatial information in EDM (spatial EDM) is an exciting direction for future research (e.g., https://doi.org/10.1111/2041-210X.13511).

We thank the reviewer for this constructive suggestion and fully agree that physical processes such as water circulation, wave action, and spatial heterogeneity can play an important role in shaping phytoplankton dynamics, particularly in open and highly dynamic aquatic systems.

In the present study, data were collected from Greifensee, a relatively small and shallow lake that is generally well mixed, which reduces—but does not eliminate—the influence of spatial heterogeneity and advective transport. Although we did not directly measure water circulation or wave dynamics, we partially accounted for episodic physical forcing by including **wind speed and thermocline depth** as explanatory variables, which serve as proxies for water movement and mixing. These variables were already discussed briefly in the Limitations section, and following the reviewer's suggestion, we have now made this point more explicit. We further agree that extending this framework to **multiple monitoring locations** and explicitly incorporating **spatiotemporal information** would likely improve predictive skill, especially in larger, more open, and more dynamic systems where harmful algal blooms also occur. While the present study focuses on a single site to isolate high-frequency biotic interactions, we now explicitly highlight the integration of physical processes and **spatial EDM approaches** as a promising and important direction for future research. We have revised the Limitations section accordingly and added a discussion of spatial EDM as an emerging extension of empirical dynamic modelling. The new paragraph (Lines 387–398) reads like this:

"Third, Greifensee is a relatively small and shallow lake that is typically well mixed; however, episodic physical processes such as wind-driven mixing and short-term water movement may still influence plankton dynamics. Although we did not directly measure water circulation or wave activity, we included wind speed and thermocline depth as explanatory variables to serve as proxies for physical forcing and mixing. Future studies that integrate direct measurements of physical processes, as well as data from multiple locations, would allow explicit evaluation of spatial heterogeneity and advective transport, which are likely to be particularly important in larger and more dynamic systems. More broadly, extending empirical dynamic modelling to explicitly incorporate spatiotemporal information represents a promising direction for future research. Recent developments in spatial EDM provide a framework for integrating spatial structure with nonlinear dynamical inference and may substantially improve prediction and mechanistic understanding of harmful algal blooms across heterogeneous ecosystems (Johnson et al. 2021)."

Minor points
[1] - Abstract: It is better to mention that the authors conducted "causal analysis," not "correlation analysis," based on nonlinear time series analysis.

Thank you for your suggestion, we changed the sentence to "Using five years of daily monitoring data from a eutrophic lake and causal inference via state-space reconstruction modelling, …" (Lines 23-26).

[2] - Line 95: The authors may clarify that they performed time-series-based causal detection analysis and used "predicted" daily physical parameters here.

Thanks for the suggestion. The new version of the mentioned text now reads: “Concurrently, we monitor all major physical and chemical parameters influencing cyanobacterial growth, including water temperature, nutrients, and meteorological conditions (Fig. 1 and Fig. S4), with all abiotic data quality-controlled and aggregated (or predicted where needed) to daily resolution for time-series-based causal analysis (see materials and methods).” (Lines 102-105).

[3] - Line 123: It is better to say " the natural logarithm" is taken instead of "Ln-transformed" for clarity.

We thank the reviewer for the suggestion and have revised the text to explicitly state that the natural logarithm of taxon densities is used.

[4]- Line 134: The authors may introduce the term "empirical dynamic modeling (EDM)" here.¨

We thank the reviewer for the suggestion. We have introduced the term now in L139-140.

[5]- Line 205: Figure 2 indicates that the benchmark model (persistence model) outperformed the MDR S-map models when the forecast horizon is from 0 to about 5 days (or sometimes more). Does this suggest that the MDR S-map did not provide meaningful predictions for the 0-5 day period? Also, is this related to the smoothing of the original time series (as the smoothing may strengthen the autocorrelation)? What would happen if EDM were applied to the original time series?

We thank the reviewer for this insightful observation. The persistence model outperforming S-maps at short forecast horizons (0–5 days) is neither surprising nor problematic—it is a well-documented feature in the ecological forecasting literature that we also reviewed and discussed in one of our previous papers (Merz et al. 2025, Proc. R. Soc. B). It was also addressed in our first-round response to Reviewer 3 (comment 8).

At forecast horizons of a few days, temporal autocorrelation in population dynamics is strong, making the assumption of "no change" (persistence model) highly accurate. Our S-map models do not explicitly include the lagged response variable Y(t-1) as a predictor—only density-dependent effects via target taxon abundance and other ecological covariates. Including Y(t-1) would likely improve short-term performance but may conflate ecological mechanisms with simple autocorrelation, which is not our goal.

The key result is that S-map predictions diverge significantly from persistence after 5–7 days (**Fig. 2**). This demonstrates that our models capture genuine ecological mechanisms—biotic interactions and environmental responses—that enable forecasting beyond the timescale of serial autocorrelation. This interpretation is further supported by our sensitivity analysis (before **Figs. S7–S8,** now Extended Data **E3-E4**), which shows that excluding temporal autocorrelation (via increased exclusion radius) reduces model skill only at short horizons but not beyond ~7 days, confirming that longer-term predictability arises from ecological processes rather than statistical memory.

Regarding smoothing effects, as demonstrated in our response to the reviewer's first comment (**Fig. R1-R5** in this document, and now documented in Supplementary Information), smoothing does not fundamentally alter the inference of ecological interactions or model performance. While smoothing may slightly strengthen autocorrelation, this does not compromise our ability to identify meaningful ecological mechanisms at relevant forecasting timescales.

In summary, the persistence model's superiority at very short horizons reflects inherent autocorrelation in ecological time series, not a deficiency in our models. The ecologically meaningful predictions occur at timescales where mechanistic understanding—not statistical inertia—drives forecast skill.

[6]- Line 357: The term "empirical dynamic modeling" should be introduced earlier.

We thank the reviewer for this suggestion. Following their earlier comment, we have now introduced the term empirical dynamic modelling earlier in the manuscript (L139–140).

[7]- Line 360: The term "embedding dimension" was not explained.

We thank the reviewer for pointing this out and have now added a brief explanation of the embedding dimension in the revised text to improve clarity (L566 –569).

[8]- Line 517: Why was the embedding dimension fixed at 15? It should be determined for each variable.

We thank the reviewer for this important comment and agree that, in principle, embedding dimension (E) should be determined for each target variable to optimise state reconstruction. We explored the optimal embedding dimension for each cyanobacterial taxon's nGR time series. We found that all targets displayed a similar profile (**Fig. R9,** included in the revised manuscript as **SI Figs. S27**). In particular, prediction skill (rho) stabilises after E = 2. It remains relatively stable up to E = 15 for all taxa, before slightly declining at higher dimensions. We initially selected **E = 15** instead of 2 for the CCM because of a **higher CCM forecasting skill** (**Figs. R10-11**). At E = 15, the absolute cross-mapping skill (rho) was higher than at lower dimensions, improving the detection of weak but meaningful interactions (**Figs. R12–13**, now also included in the revised manuscript as **SI Figs. S28 and S29**). In addition, we observed **a clearer separation between the original signal and the seasonal surrogates** (**Fig. R14**, now also included in the revised manuscript as Extended Data **Fig. E1**). A larger number of embedding dimensions allows a more reliable distinction between causal interactions and seasonally driven associations (**Figs. R12-14**).

However, to harmonise the CCM analysis with our multiview regularised S-map analysis (for which used E = 10), we have recomputed the CCM results using **E = 10** in the revised manuscript (**see Methods Section 3.1, Fig.1** and Extended Data **Fig. E2**). The results remain highly consistent with the previous analysis: the rank order of variables varies only slightly with increasing E (**Figs. R12-14**) and overall conclusions are unchanged, confirming that our findings are robust to the choice of embedding dimension.

redacted

**Fig. R9: Simplex projection results for all target cyanobacteria across embedding dimensions.** Simplex projection was used to evaluate prediction skill across a range of embedding dimensions (E = 1–20). Models were fitted using different initial library sizes (20%, 40%, 60%, and 80% of the time series) and were used to predict the subsequent 20% of the dataset. The y-axis shows forecasting skill (rho, Pearson correlation coefficient), and the x-axis shows the tested embedding dimensions. Colours indicate the initial library size used for model fitting. Across all target cyanobacterial taxa, prediction skill remains relatively stable across embedding dimensions, indicating limited sensitivity to the choice of E.

redacted

**Fig. R10: CCM forecasting skill across embedding dimensions (seasonal surrogate time series).** Mean cross-mapping skill (rho) at maximum library size (80%) across embedding dimensions (E) for each predictor variable. Panels correspond to individual cyanobacterial target taxa. The y-axis shows mean rho and the x-axis shows embedding dimension. Colours identify predictor variables included in the CCM analysis. Shaded bands represent ±1 s.e. across 100 seasonal surrogate time series. Across taxa and variables, mean rho generally increases with embedding dimension.

redacted

**Fig. R11: CCM forecasting skill across embedding dimensions (original time series).** Mean cross-mapping skill (rho) at maximum library size (80%) across embedding dimensions (E) for each predictor variable in the original time series. Panels correspond to individual cyanobacterial target taxa. The y-axis shows mean rho and the x-axis shows embedding dimension. Colours identify predictor variables tested in the CCM analysis. As in the seasonal surrogate analysis, mean rho generally increases with embedding dimension across taxa and predictors, indicating consistent scaling of cross-mapping skill with E.

redacted

**Fig. R12: Variable ranking across embedding dimensions using seasonal surrogate CCM.** Rank dynamics of predictor variables across embedding dimensions (E = 2, 7, 10, 15) based on cross-mapping skill (rho) at maximum library size (80%). Each panel corresponds to one cyanobacterial target taxon. Within each panel, variables are ranked (y-axis; 1–23) from highest (rank 1) to lowest rho at each embedding dimension (x-axis). Colours denote the ranking of variables in the seasonal surrogate CCM at E = 15 and are held constant across embedding dimensions and across figures (**Fig. R13**) to facilitate comparison. Solid lines represent biotic variables; dashed lines represent abiotic variables. White symbols indicate variables not identified as causal at the daily scale (original-series rho < seasonal-surrogate rho, **see Methods section 3.1 Convergent Cross-Mapping**), whereas coloured symbols indicate variables classified as causal. Top-ranked variables remain largely stable across embedding dimensions (typically shifting by ≤1–2 ranks). In the seasonal surrogate analysis, highest-ranked predictors are predominantly abiotic variables, consistent with strong seasonal forcing.

redacted

**Fig. R13: Variable ranking across embedding dimensions using original time series CCM.** Rank dynamics of predictor variables across embedding dimensions (E = 2, 7, 10, 15) based on cross-mapping skill (rho) at maximum library size (80%) for the original time series. Panels correspond to individual cyanobacterial target taxa. Variables are ranked (y-axis; 1–23) from highest to lowest ρ for each embedding dimension (x-axis). Colours correspond to the ranking derived from the seasonal surrogate CCM at E = 15, identical to **Fig. R12**, enabling direct comparison between seasonal and original analyses. Line types denote biotic (solid) and abiotic (dashed) variables. White symbols indicate non-causal variables at the daily scale, whereas coloured symbols denote variables meeting the causal criterion (original-series rho > seasonal-surrogate rho, **see Methods section 3.1 Convergent Cross-Mapping** ). Across embedding dimensions, the relative position of top-ranked variables remains stable. In contrast to the seasonal surrogate results—where abiotic drivers dominate the highest ranks—the original time series reveals a mixture of biotic and abiotic predictors among the top ranks (often predominantly biotic), indicating causal influences on daily cyanobacterial growth beyond seasonal structure. Separation between original and seasonal surrogate signals increases at higher embedding dimensions.

redacted

**Fig. R14: Cumulative contribution of abiotic predictors across ranked CCM variables, with increasing embedding dimensions.** Cumulative percentage of abiotic variables among predictors ranked by convergent cross-mapping (CCM) skill (rho) at maximum library size (80%) (**see Figs. R12-R13**). For each cyanobacterial target taxon (columns) and embedding dimension (rows), variables were ordered from highest to lowest rho. The x-axis represents the progressive inclusion of ranked variables, and the y-axis shows the cumulative percentage of abiotic variables among those included ranks. Line colours distinguish CCM results from the original time series and seasonal surrogate analyses. Across embedding dimensions, **seasonal surrogate** CCM results (blue) show a consistently higher proportion of abiotic variables among the highest ranks, reflecting strong seasonal forcing. In contrast, the **original time series** (red) shows a lower cumulative percentage of abiotic predictors at the top ranks, indicating that biotic interactions contribute substantially to daily cyanobacterial growth dynamics. This pattern remains consistent across embedding dimensions, showing the independence of our inference from the choice of embedding dimension.

[9]- Line 531: The term "EDM" was not defined before.

We thank the reviewer for this suggestion. Following their earlier comment, we have now introduced the term empirical dynamic modelling earlier in the manuscript (L139–140).

[10]- Line 532: CCM was conducted using TP=0, while the MDR S-map was conducted using TP=1. Why didn't the authors use TP=-1 for CCM?

We thank the reviewer for raising this important point. Our goal with the CCM analysis was to identify which variables directly influence cyanobacterial net growth rate (nGR). For this purpose, we

initially used Tp = 0 (prediction horizon) to test for direct links between predictor variables and contemporaneous nGR.

For the S-map analysis, the model is formulated following a growth model similar to a Lotka–Volterra framework (as in Nguyen et al., 2025, PNAS), where the change in growth rate from time (t) to (t+1) is predicted by the abundance of the target species and the abundances of interacting species at time (t) - see the explanation to reviewer #3 concern (1).

Because our dataset is daily and these biotic interactions occur at a daily scale, we do not expect substantial differences in cross-map skill between Tp = 0 and Tp = -1. Nevertheless, to verify this, we replicated the CCM analysis using Tp = -1 (**Figs. R15-R16**). The results were highly consistent with the original Tp = 0 analysis.

Thanks to the reviewer comment, to maintain methodological consistency with the S-map formulation, we have now updated **Figs. 1** and **E2** to display CCM results using **Tp = -1** (with an embedding dimension of 10 – see minor comment 8 for further explanation). This improves methodological consistency and confirms that the overall conclusions of the study remain unchanged.

redacted

**Figure R15: Comparison of CCM forecasting skill (rho) at TP = 0 and TP = -1.** Mean cross-mapping forecasting skill (rho) at maximum library size (80%) for each predictor variable (y-axis), across different cyanobacterial target taxa (columns). **The top panel** shows results for the original time series; error bars indicate ±1 standard deviation across subsample calculations. **Bottom panel** shows results for seasonal surrogate time series; error bars indicate the standard error of the mean across 100 surrogates for each variable pair. Colours indicate forecast horizon with blue representing Tp = 0 and red Tp= -1. Overall, cross-mapping skill is similar for all tested variables regardless of forecast horizon. CCM was performed with an embedding dimension of 15.

redacted

**Figure R16: Convergence test of CCM across forecast horizons.** P-values from a one-sided t-test evaluating convergence of cross-mapping forecasting skill (rho) between minimum (10%) and maximum (80%) library sizes for each predictor variable. **Columns** correspond to different target cyanobacterial taxa, and **rows** correspond to forecast horizons (Tp). Orange-filled dots show p-values for the original dataset, while blue open dots show p-values for the 100 seasonal surrogate time series. Across all forecast horizons, convergence is observed for all variables, indicating that cross-map skill increases with library size. CCM was performed with an embedding dimension of 15.

[11]- Figure 1A: It should be clarified on the y-axis that these are also "Ln-transformed" values.

We thank the reviewer for pointing this out. To clarify, the variables shown in Fig. 1A are scaled but not natural-logarithm transformed, as indicated in the figure legend, whereas panels B–D display natural-logarithm–transformed densities, which are already specified on the corresponding y-axes.

# Response to reviewer’s comments

We thank Reviewer #3 and Reviewer #4 for their careful evaluation of our manuscript throughout the review process.

We are especially grateful for the thoughtful and constructive comments provided during the different rounds of review. The suggestions greatly helped us strengthen the robustness, clarity, and methodological transparency of the study. We appreciate the reviewer’s positive assessment of the revised manuscript and are pleased that the concerns raised have been satisfactorily addressed.

We also thank the editor and all reviewers for their time, expertise, and constructive feedback throughout the evaluation process.
